# Binding site discovery from nucleic acid sequences by discriminative learning of hidden Markov models

**DOI:** 10.1093/nar/gku1083

**Published:** 2014-11-11

**Authors:** Jonas Maaskola, Nikolaus Rajewsky

**Affiliations:** Laboratory for Systems Biology of Gene Regulatory Elements, Max-Delbrück-Center for Molecular Medicine, Robert-Rössle-Strasse 10, Berlin-Buch 13125, Germany

## Abstract

We present a discriminative learning method for pattern discovery of binding sites in nucleic acid sequences based on hidden Markov models. Sets of positive and negative example sequences are mined for sequence motifs whose occurrence frequency varies between the sets. The method offers several objective functions, but we concentrate on mutual information of condition and motif occurrence. We perform a systematic comparison of our method and numerous published motif-finding tools. Our method achieves the highest motif discovery performance, while being faster than most published methods. We present case studies of data from various technologies, including ChIP-Seq, RIP-Chip and PAR-CLIP, of embryonic stem cell transcription factors and of RNA-binding proteins, demonstrating practicality and utility of the method. For the alternative splicing factor RBM10, our analysis finds motifs known to be splicing-relevant.

The motif discovery method is implemented in the free software package Discrover. It is applicable to genome- and transcriptome-scale data, makes use of available repeat experiments and aside from binary contrasts also more complex data configurations can be utilized.

## INTRODUCTION

Transcriptional and post-transcriptional regulation rely to a large extent on effective mechanisms that allow nucleic acid binding proteins to recognize specific sets of nucleic acids. Aside from structural cues, binding of regulators is guided by sequence information (motifs) present in cognate nucleic acids. Motif discovery (MD) is the problem of unraveling motifs recognized by a given nucleic acid binding protein from sequences known to harbor occurrences of the motif.

Classically, MD was marked by scarcity of data when only few sequences were available. The introduction of microarray-based technologies like ChIP-chip (1,2) and RIP-Chip (3,4) allowed to assay *in vivo* sequence binding specificity on genome- and transcriptome-scale. More recently, sequencing-based technologies, such as ChIP-Seq (5,6) and CLIP-Seq (7–9) further increased the amount of data yielded by single experiments and simultaneously improved the spatial resolution, reducing uncertainty about the exact location of *in vivo* binding sites. SELEX (10,11) and related sequencing-based technologies (12), and protein-binding microarrays (13,14) are targeted assays for the *in vitro* sequence binding specificity of nucleic acid binding proteins.

Due to the central importance of the MD problem in computational biology, many algorithms addressing it have been developed over the last two decades (15). These algorithms employ a variety of models for the sequence binding specificity of nucleic acid binding proteins, including discrete word-based models, as well as probabilistic models such as position weight matrices (PWMs) (16) and hidden Markov models (HMM) (17). Word-based approaches tend to be computationally efficient and allow fast global optimization, but may fail for motifs that include weak positions (15). PWMs can be motivated from biophysical principles (18–20). General inference methods for HMMs offer a unified framework for biological sequence modeling (21). HMMs model both binding sites and their surrounding sequence context, may account for interacting neighboring positions (illustrated in Supplementary Figure S4), and length variability of motifs can be idiomatically realized via insert and deletion states (22,23).

Because of historically smaller data sizes, many commonly used MD methods, such as MEME (24), are not designed for data sets as large as those produced by current experiments, aborting or running impractically long when applied to large data sets. Thus, even after more than two decades of computational analysis of biological sequences, there is continued interest in the development of new analysis methods that leverage the full potential of large data sets.

Here we describe a discriminative learning method based on HMMs, available as free software, to automatically discover binding-site sequence motifs of nucleic acid binding proteins from arbitrary contrasts, such as positive and negative example sequences. Not all of the positive examples need to contain motif occurrences and not all negative examples need to be devoid of them. The framework is applicable to a broad variety of contrasts, including the comparison of strongly bound versus weakly bound targets, or of signal sequences with shuffled sequences. It is also possible to discover context-dependent motifs, or to analyze data sets of different factors for mutually discriminative features. When available, information from repeat experiments is leveraged by the method.

We study MD performance of our and published methods in a controlled setting on synthetic data. The method is applied to real biological data sets, among them RIP-Chip and PAR-CLIP data of RNA-binding proteins (RBPs): the Pumilio and FBF (PUF) family of post-transcriptional regulators in diverse species (25), and the human alternative splicing regulator RBM10 (26). We also demonstrate the utility of the method for ChIP-Seq data of mouse transcription factors (TFs).

### Modeling only positive example sequences

The goal of MD is characterizing the properties of cognate motifs. Thus, positive example sequences containing the motifs are frequently collected, and the common pattern is extracted. One way of doing this is by finding a generative model of the data, i.e. a statistical model that simulates the data well. Maximum likelihood estimation is often used for this purpose because it has many beneficial properties (27), most notably consistency, asymptotic normality and efficiency.

For the purposes of this manuscript we will refer to MD techniques based only on positive examples as signal-only learning. Most classical MD methods, such as GibbsSampler (28), MEME (24), BioProspector (29) and MDscan (30), are generative learning methods modeling only signal sequences.

### Modeling multiple classes of sequences

High-throughput technologies led to a shift from small, well-curated sets of sequences toward large sets of sequences that also contain false-positive examples. Yet, even for true-positive example sequences the exact location of the cognate motif within the sequence is not known. A solution to these difficulties is offered by discriminative motif discovery (DMD) methods (31–48). Such methods leverage positive and negative example sequences (i) to help recognize false-positive sequences and (ii) to discern motif and non-motif positions within true-positive example sequences. In general they operate with multiple sets of sequences, and strive to identify motifs whose occurrence frequency differs between the sets. Formally, the data are then a set of pairs of classes *C* and sequences $\boldsymbol{X}$, $\left\lbrace \left\langle C, \boldsymbol{X}\right\rangle \right\rbrace$.

Generative learning approaches are also possible in this setting by optimizing the likelihood of classes and sequences, $P(C,\boldsymbol{X}|\boldsymbol{\theta })=P(C|\boldsymbol{X},\boldsymbol{\theta }) P(\boldsymbol{X}|\boldsymbol{\theta })$. As the likelihood is typically dominated by the contribution due to the sequences, $P(\boldsymbol{X}|\boldsymbol{\theta })$, discriminative learning approaches often aim to optimize the conditional class probability, $P(C|\boldsymbol{X}, \boldsymbol{\theta })$, which may yield better classifiers for the class *C* (49–51).

### Learning features based on sequence motifs

DMD, however, is not so much interested in classifying the sequences as belonging to a given class *C* but rather in learning certain sequence features, in particular the presence of motifs. As such features are generally not observed, but must be inferred, DMD may also be perceived as a (discriminative) feature discovery problem, in which a variety of objective functions can be chosen to elicit relevant features.

Reasoning that a sequence exhibiting a motif at least once might be sufficient for recognition of the sequence, we use as feature the question whether a sequence has *at least one occurrence* of a given motif. Alternatively, because multiple occurrences of a motif in a sequence might induce a stronger regulatory response, also the *number of occurrences per sequence* may be a relevant feature (not considered here).

### Setting up contrasts for DMD

**Figure 1.**
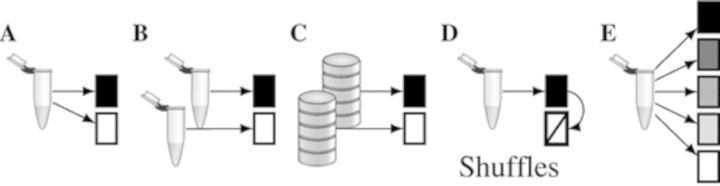
Contrasts for discriminative sequence analysis. Test tubes represent samples from different biological conditions, boxes are sets of sequences with blackness indicating the true positive rate in the set. (**A**) Binary constrast from a single biological sample, e.g. bound and not bound, or expressed and not expressed. (**B**) Binary contrast of different biological conditions, e.g. pull-down and mock, two different tissues, cell-types or treatments. (**C**) Binary contrast of sequences from two different database searches. (**D**) Binary contrast in which the data of the contrasting condition are synthesized from the signal data by shuffling. (**E**) Contrast from grading the signal strength.

The choice of control data naturally strongly affects the chances of successfully discovering the signal. A minimal contrast for DMD is that in which a single experiment yields evidence for binding to one set of sequences, and no evidence for another, see Figure 1A. Another suitable binary contrast may be to compare the sequences that have more binding evidence in the first of a pair of experiments with those that have more evidence in the second, or that come from different database queries, as depicted in Figure 1B and C. Examples of such situations are cross-comparisons of different, potentially interacting binding factors, or of one factor in different conditions. In case no suitable biological control is available, it is possible to synthesize a control set of sequences, as in Figure 1D, e.g. by shuffling signal sequences, keeping word frequencies up to some order, often dinucleotides. Another possibility is that an experiment gives rise to a binding evidence rank order of the sequences. Then data may be grouped by their ranks, as in Figure 1E, to study which rank-grouping is useful to elicit the signal. Combinations of the above are also possible, as in the case when contrasting strongly bound, weakly bound and synthetic control sequences.

### Leveraging repeat experiments

Frequently data from repeat experiments are available. While in such cases we may apply multiple independent analyses to see whether the results are consistent, it is also possible to analyze repeat experiments jointly so as to increase statistical power and sensitivity.

### Motif-finding objectives

MD methods use many different measures to quantify relevance of motifs. Generative, signal-only learning uses the likelihood of a probabilistic motif model for this. Discriminative learning employs one of various measures to quantify association of motif occurrence with conditions of a contrast. Here, we briefly describe those available in our framework and related published methods. Mathematical definitions are given in the supplementary text.

Many measures of association are based on occurrence statistics in contingency tables, but some depend in other ways on the data. Some objective functions are directional, i.e. motifs that maximize them are not only differential but in fact enriched in the signal sequences. For non-directional objective functions it is possible to filter differential motifs for enrichment in the desired sample.

We will first discuss contingency table based association measures. Table 1 illustrates how some of these quantify the association of motif occurrences with conditions in several small hypothetical data sets.

**Table 1.**
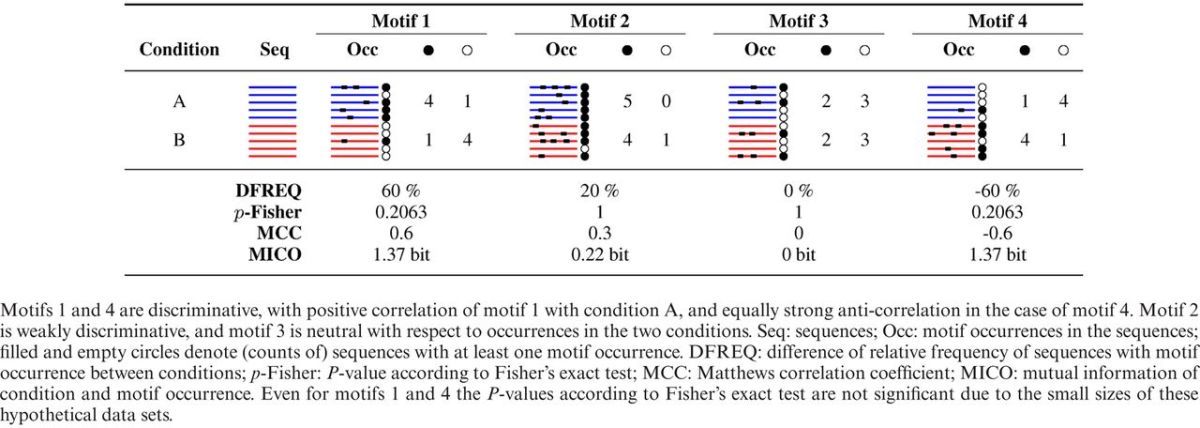
Comparison of discriminative performance of four sequence motifs in a contrast of two conditions.

#### Relative frequency difference

The simplest of these is the difference in relative occurrence frequency between signal and control (DFREQ). It may only be applied to binary contrasts. It is used by the MD tools DIPS (31) and DECOD (32), which both use motif occurrence per position as feature.

#### Normalized enrichment score

$z$-score based measures of association may be used when there is one signal sample and multiple control samples. These measure the deviation of the frequency in the signal sample from the mean of the frequency in the control samples in units of standard deviations of frequency in the control samples. Such scores are used by the discriminative motif finders YMF (33–35) and CMF (36), which also use motif occurrence per position as feature.

#### Classical association measures

The χ^2^ test is a classical measure of association in contingency tables not currently used as DMD objective. The motif finder of (37) and ALSE (38) build on the hypergeometric test for enrichment. The related Fisher's exact test is used by DREME (39) for MD.

#### Correlative measures

Matthews correlation coefficient (MCC) as DMD objective quantifies the correlation between the occurrence of a motif in a sequence and the conditions of binary contrasts. It has not previously been used for MD.

#### Mutual information of condition and motif occurrence

Mutual information of two random variables is a symmetric measure from information theory that quantifies how much uncertainty about the value of one variable is reduced by knowing that of the other (52–54). Used as discriminative objective function for MD, mutual information of condition and motif occurrence (MICO) identifies motifs whose presence or absence in a sequence of interest is most informative about which condition the sequence originated from. MICO has recently been used in discrete optimization based MD for nucleic and amino acid sequences, respectively by FIRE (40) and FIRE-pro (41), and for context-free grammars of RNA sequence-structure motifs by TEISER (42).

#### Maximum mutual information estimation

Mutual information has been used for discriminative learning with HMMs in a related but different manner for speech recognition problems in the maximum mutual information estimation (MMIE) framework (55,56), which maximizes the conditional class probability $P(C | \boldsymbol{X}, \boldsymbol{\theta })$ or, equivalently, minimizes the empirical error rate (57). Unlike the other discriminative objective functions mentioned so far, MMIE is not contingency table based. MD tools built on MMIE include DEME (43), MoAn (44) and Dispom (45).

#### Likelihood difference

Another discriminative objective function that is not based on contingency tables is the difference of log likelihoods of the signal and control data (DLOGL). Like DFREQ and MCC, it is only applicable to binary contrasts. DLOGL is used for DMD by DME (46–48).

## MATERIALS AND METHODS

We present a method based on discriminative learning to discover sequence motifs of protein binding-site patterns in nucleic acid sequences. It is implemented as a multi-threaded C++11 program Discrover (portmanteau of *discriminative* and *discover*), available at https://github.com/maaskola/discrover under the GNU General Public License v3. The package also provides facilities to generate sequence logos (58) and a module for usage in Galaxy (59), see Supplementary Figure S8.

### Overview

The method consists of three parts: seed finding, HMM optimization and significance filtering. It may be applied to RNA- or DNA-binding factors, by optionally also taking the reverse complementary strand into account. It offers the following selection of measures to identify relevant motifs:

- Log likelihood
- Log likelihood difference (DLOGL)
- Relative occurrence frequency difference (DFREQ)
- Matthews correlation coefficient (MCC)
- Maximum mutual information estimation (MMIE)
- Mutual information of condition and motif occurrence (MICO).

Measures DFREQ, MCC and MICO depend on statistics of counts of sequences that have at least one occurrence of the motif. Where during discrete optimization integer counts of sequences are used, optimization of HMMs involves expected counts of sequences. Log likelihood and its difference may only be used in HMM optimization, as they are not applicable to the non-probabilistic nature of the seed finding method. However, relative occurrence frequency of IUPAC regular expressions (REs) and its difference appear as suitable objectives for seeding HMMs to be optimized by likelihood or its difference. Similarly, the probabilistic nature of MMIE precludes its application in the discrete optimization during seed finding; in this case MICO is used for seed finding.

Discriminative objective functions minimally require binary contrasts in the form of a pair of signal and control sequence sets. Contrasts with more than two conditions can be utilized with MICO or MMIE as objective function. With the exception of MCC, all discriminative objective functions support the joint analysis of multiple contrasts.

To allow both manual experimentation and automation, seed finding has been integrated into the HMM preprocessing, but may also be run separately.

### Seed finding

Seed finding heuristically identifies motifs in the form of IUPAC REs that score high according to the chosen objective function. The seed finding procedure presented here is similar to DREME (39), but offers multiple, contingency table based objective functions to choose from, and uses a different heuristic to filter unpromising candidate motifs. We refer to this seed finding method as Plasma. Input to seed finding consists of sets of sequences among which discriminative motifs are suspected. Parameters include the choice of association measure and motif lengths to consider. The association measures comprise relative occurrence frequency, difference of relative occurrence frequency, MCC and MICO (see supplementary text for details). Optionally, differential motifs may be filtered for enrichment in specific samples.

#### Algorithm

For each occurring word $w$ of a given length the number of sequences that contain $w$ is determined for each of the sets of sequences. From these counts, and the number of sequences in each set, the objective function is evaluated for each word, and the words are sorted according to it. Then only the top *n* words are retained, where *n* is a parameter whose default value is 100.

Each retained word $w$ is generalized by generating all IUPAC generalization of $w$ that differ from $w$ by allowing one additional nucleotide at any position. For example, the word ACG may be generalized to [AC]CG, [AG]CG, [AT]CG, A[AC]G, A[CG]G, A[CT]G, AC[AG], AC[CG], AC[GT]. By scanning over the sets of sequences, occurrence statistics are procured for each of the generalizations. Subsequently the objective function is computed from the statistics of the generalizations. Generalizations with a score less than any of their generating specializations are dropped, and the resulting top *n* generalizations are kept for further rounds of adding degeneracy, scoring and retaining the top.

This scheme is iteratively continued until no further generalizations are available either because all have been dropped or because the maximal degeneracy has been reached. The user may also limit the maximally allowed degeneracy with an absolute or relative limit.

#### Multiple seeds

When multiple seeds are desired, the most relevant one is identified according to the algorithm described above. Subsequently, all occurrences of this motif are masked from the sequences, and further seeds may be sought. Alternatively, instead of masking just occurrences, the sequences containing occurrences may be discarded for the identification of further seeds.

### Binding-site HMM

Next, we describe the details of the binding-site HMM, whose topology is illustrated in Supplementary Figure S5. It comprises a background state and a chain of states for each motif. The background state may transition to itself or to the first state of each chain. The last state of each chain may transition to the background state or the first state of any motif chain. To increase flexibility of the binding-site models, the user may manually allow single (not self-transiting) insert positions between any two adjacent motif chain states. For technical reasons, the topology includes a special start and end state.

#### Initialization

First an HMM consisting only of a state for the background is trained using the Baum–Welch algorithm (17,60). The user may specify which of the sequence sets should be used for this.

Seeds may be specified in the form of IUPAC REs as provided by the seed-finding program. Then, for each of the seeds, a motif chain is added, and wired up with the background state. The transition probability *P* from the background state to the motif chain is initialized to $P=\frac{1}{l-w+1}$, where $w$ is the length of the seed and *l* is the average sequence length, such that one such transition is expected per sequence unless the emission probabilities are also taken into account.

The initial emission probabilities of the motif chains are centered on the IUPAC seed sequence: each nucleotide that is not allowed in the seed at a given position is assigned an emission probability of *α*, so that the *m* nucleotides that are allowed at that position each have an emission probability of $\frac{1-\alpha (4-m)}{m}$. With the default value *α* = 0.03 and for the IUPAC code A this results in a probability distribution of (0.91/0.03/0.03/0.03), and the IUPAC code W = [AT] yields (0.47/0.03/0.03/0.47).

#### Posterior probability

As mentioned above, the relevant statistic used in this method is that of sequences that have at least one motif occurrence. The corresponding probabilistic notion for an HMM with parameters $\boldsymbol{\theta }$ is the posterior probability of a sequence $\boldsymbol{X}$ having at least one motif occurrence, $P(k>0|\boldsymbol{X},\boldsymbol{\theta })$, where *k* is the number of motif occurrences. The supplementary text explains the details of the computation of posterior probabilities. The posterior probability of all sequences in a sequence set is evaluated and summed to yield the expected number of sequences in the set that have at least one motif occurrence. These probabilistic counts are then used to compute discriminative statistics.

#### Strandedness

Our method has two modes of operation: a single-stranded mode for RBP analysis and a double-stranded mode for the analysis of DNA-binding proteins (DBPs). The single-stranded mode only considers motif occurrences on the forward strand. The double-stranded mode concatenates to sequences their reverse complements separated by a special symbol, and then proceeds to discover motifs on the forward strand of so-extended sequences.

### Gradient-based learning

Our method performs discriminative learning by gradient optimization, a local search technique. In each iteration of gradient optimization, the gradient of the chosen objective function is computed and used to improve the current parameter estimate. For this, a line search is performed in the direction of the gradient, using the Moré–Thuente algorithm (61) to ensure sufficient increase and proximity to the local maximum along the search direction.

Mathematical details of the gradient calculations are given in the supplementary text, where we provide efficient and numerically robust expressions to calculate the gradients of the objective functions. As the discriminative objective functions depend on the likelihood either directly or indirectly via the posterior motif occurrence probability, we also give expressions for the gradient of likelihood and posterior probability.

We note that the gradient of HMM likelihoods is efficiently computed (62), in the sense of having a runtime complexity linear in the length *T* of the data, *O*(*TE* + *NM*), where *N* is the number of HMM states, *M* the number of emissions, *E* is the number of edges in the HMM topology with *N* ≤ *E* ≤ *N*^2^. In terms of the length of data *T*, this is the same complexity as that of the forward–backward algorithm of *O*(*TE*) (17). In order to simplify the optimization numerically, we avoid the renormalization during gradient optimization by using expressions of slightly larger runtime, *O*(*TE* + *EN* + *NM*^2^), which is however still linear in the size of the data. The remaining calculations to determine the gradient of the objective functions from the likelihood gradient do not increase the asymptotic runtime complexity. Supplementary Table T3 summarizes the runtime complexities of the different steps of the gradient calculations.

Because HMM calculations can be done in parallel as contributions from different sequences are independent of each other, Discrover uses the OpenMP library to make use of multiple CPU cores present in most modern workstations.

### Learning scheme

#### Signal and context parameters

Binding-site HMMs are composite models of the cognate motifs as well as the surrounding sequence context. Some parameters of binding-site HMMs, in particular the motif chain state emission probabilities, pertain to signal features, and we refer to these as *signal parameters*. The other parameters are referred to as *context parameters* and comprise the emission probabilities of the background and all transition probabilities, including the prior occurrence probabilities of the motifs, realized as transition probabilities from other states to the beginning of the respective chain of motif states.

We assume that only the presence of signal features differs between signal and control sequences, while the surrounding sequence context is shared. Therefore, we propose to employ discriminative learning principles to learn signal parameters by contrasting signal and control sequences. However, to leverage HMM learning methods, we must specify a complete set of HMM parameters, including the context parameters. When context parameters are learned uninformed of signal features, they may erroneously incorporate properties of the signal features. There is thus a mutual dependence of the learning problems of signal and context parameters. In order to resolve it we propose the following procedure.

#### Hybrid learning scheme

We associate objective functions to the signal and context parameters. The signal parameters will use a discriminative objective, and the context parameters a generative one. We then employ a hybrid learning scheme which aims to jointly optimize both objectives over their respective parameters. The scheme consists of alternatingly updating the signal and context parameter classes, until termination criteria for both parameter classes are simultaneously fulfilled.

The natural choice for the generative objective function is the likelihood. Thus, updates for the context parameters are performed using iterations of the Baum–Welch algorithm. Signal parameters may be optimized for any of the implemented discriminative objective functions by performing iterations of gradient search.

#### Choice of learning scheme and alternatives

This hybrid learning scheme in which only the motif emissions are optimized by discriminative objectives and all other parameters are optimized by the Baum-Welch algorithm is used by default in Discrover. It is not guaranteed to terminate in the general case of arbitrary data and arbitrary choices of generative and discriminative objective functions. Yet, in our experience such problems are rare. In any case, to practically address the termination problem, the user may specify a maximal number of iterations to perform.

Aside from the hybrid learning scheme, Discrover also allows the user to train all parameters by one objective function, or only the motif emissions and leave other parameters unmodified. Should the hybrid learning scheme fail for some data, these alternative, single-objective learning schemes are expected to optimize more robustly.

#### Sequence sets for learning context parameters

By definition, the occurrence frequency of discriminative motifs differs between sets of sequences of suitable contrasts. Thus, it matters which set of sequences the occurrence prior is learned from. By default all sequence sets are used to train the context parameters, but the user may specify a subset of the sequence sets to train the context parameters on. For example, for contrasts of signal and scrambled sequences, context parameters may be learned from the signal data only.

#### Learning MMIE parameters

Differently from the other objectives, the optimization of the MMIE objective requires learning of class priors *P*(*C*) and conditional motif occurrence probabilities in the classes *P*(*M*|*C*) in addition to the HMM parameters. It has been suggested to optimize these separately from the other parameters (62). Accordingly, Discrover separately re-estimates them during each iteration of learning, after the HMM parameters have been updated.

### Significance of association, multiple testing correction and significance filtering

In this work we make use of the following connection between mutual information and the likelihood ratio test which allows to compute *P*-values for mutual information in contingency tables. The value of mutual information *I* is related to the log likelihood ratio log Λ of the hypothesis that the counts in rows and columns of a contingency table are distributed independently by log Λ = −*I* × *n* × log 2, where *n* is the total number of cases in the table. Wilks’ theorem (63) relates the log likelihood ratio to the χ^2^ test. Specifically, for *k* × 2 contingency tables and for increasing sample sizes, −2log Λ is asymptotically distributed like χ^2^ with *k* − 1 degrees of freedom.

In MD frequently the problem arises to compare the performance of models with differing numbers of parameters. If two models are optimal for their respective motif spaces, and when additionally the larger of the two motif spaces comprises the smaller one, then discriminability must be greater or equal for the motif which is optimal over the larger motif space. We determine *P*-values in both cases as described above. By correcting *P*-values for motif space size, we propose to make comparable the discriminability of motifs with different numbers of parameters. To this end we correct *P*-values in a Bonferroni-style by multiplying with the motif space size. This counteracts usage of overly long motifs, for which the search space is large, by favoring short words, with a correspondingly smaller search space. The supplementary material explains how we determine motif space size.

Rejecting models whose multiple testing corrected *P*-values are not significant reduces the number of falsely predicted models. Thus, Discrover accepts or rejects the final, optimized parameterization, depending on whether the corrected MICO-based *P*-value meets a given threshold. This discriminative significance filtering based on MICO is applied regardless of the objective function chosen for seeding and optimization.

### Finding multiple motifs

In MD applications it is frequently useful to discover more than the single best-scoring motif. In particular, when the cognate motif of a factor is less enriched than other more recognizable motifs, it may be necessary to consider suboptimally scoring motifs. Also, e.g. ChIP-Seq data often contain motifs of associated co-factors.

For this purpose our framework offers a MICO-based procedure designed to yield a non-redundant set of motifs with maximal discrimination between the conditions. It first finds seeds and independently optimizes HMMs for each, selecting the best according to MICO-based *P*-value (Supplementary Figure S6). Then, progressively more motifs are added that (a) have sufficient residual discriminatory contribution after accounting for previously accepted motifs and (b) are not redundant with previously accepted motifs (Supplementary Figure S7). This is ensured by filtering based on conditional mutual information (cMI) in two ways.

We determine (i) cMI of conditions of the contrast and occurrences of the newly added motif given occurrences of previously accepted motifs (cMICO) and (ii) cMI between occurrences of new and previous motifs given the conditions of the contrast (motif pair cMI) (definitions in the supplementary material). cMICO quantifies the discriminatory contribution of the new motif after accounting for previous ones, while motif pair cMI quantifies association between occurrences of the new and previous motifs.

To respectively ensure (a) and (b), motifs are discarded if their cMICO-based *P*-value is not significant, or if their ratio of cMICO over motif pair cMI does not meet a threshold. Importantly, these two criteria are enforced pairwise against each previously accepted motif, and jointly against all previously accepted motifs together.

After filtering, the HMM whose new motif achieves the best cMICO-based *P*-value is selected and re-trained to optimize MICO for the feature of sequences having at least one occurrence of any of its motifs. If the MICO-based *P*-value improves over the previously accepted one's, this HMM is accepted, and further motifs may be added. Otherwise, or if all candidate motifs have been discarded, the last accepted HMM is reported.

### Materials

#### Synthetic data

Synthetic data were generated as follows for three sets of experiments that we respectively refer to as basic, 3′UTR and decoy experiments. The parameters varied in the experiments are summarized in Table 2, and include length and number of sequences, information content (IC) (58) and implantation frequency of signal (and decoy) motifs.

**Table 2.** Parameters for the generation of synthetic sequence data.

	Basic	3′UTR	Decoy
Sequence background	Uniform, zeroth-order MC	Human 3′UTR	Uniform, zeroth-order MC
Sequence length [nt]	20, 50, 100, 200, 500, 1000	20, 50, 100, 200, 500, 1000	100
Sequence number	100, 1000, 10 000	100, 1000, 10 000	10 000
Motif length [nt]	8	8	8
Implanted signal motifs per sequence	0 or 1	0 or 1	0 or 1
Signal motif implantation probability [%]	1, 2, 5, 10, 20, 50, 100	1, 2, 5, 10, 20, 50, 100	10
Signal motif IC [bit]	0, 2, 4, 6, 8, 10, 12, 14, 16	0, 2, 4, 6, 8, 10, 12, 14, 16	0, 2, 4, 6, 8, 10, 12, 14, 16
Implanted decoy motifs per sequence	0	0	0 or 1
Decoy motif implantation probability [%]	0	0	1, 2, 5, 10, 20, 50, 100
Decoy motif IC [bit]	NA	NA	0, 2, 4, 6, 8, 10, 12, 14, 16
Strandedness	Single-stranded	Single-stranded	Single-stranded
Total experiments	1134	1134	567

MC = Markov chain; IC = information content.

For each data-generation parameter setting a pair of signal and control sequence sets is generated. A signal motif with a specific IC is generated by choosing a random PWM and polarizing (exponentiating component-wise and renormalizing) so as to achieve the desired IC. Each sequence is generated according to the background model of the set of experiments it is part of. Then, motifs are implanted into the signal sequences. Signal sequences are selected with a given implantation probability, and for each selected sequence one signal motif occurrence is generated from the PWM and inserted into the sequence at a random position.

All motifs are inserted on the sense strand, simulating an RNA MD experiment. The three sets of experiments differ by the choice of the sequence context into which motifs are inserted. The basic and decoy experiments use a uniform, zeroth-order Markov chain to generate synthetic sequences, while the 3′UTR experiments use sequences sampled from human 3′UTRs. In the decoy experiments, before implanting the signal motifs, occurrences of decoy motifs are implanted both into signal and control sequences. The data sets are available from the Rajewsky lab web page.

How well motifs can be discovered depends on the difficulty of recognizing the motif when it is already known. As a reference we evaluate motif recognizability, which we define as the predictive performance of the true model. As an approximation to the true model we use HMMs comprising a background state and a motif chain with emission probabilities equal to the implanted PWM. The transition probability from the background state to the motif state is chosen such that the expected number of motifs per sequence is equal to the implantation frequency of the experiment, i.e. if implantation frequency is 10% and if the sequence length is 100 then the per-position probability of transiting from the background state to the motif chain is set to 0.1 × 0.01 = 0.001. The background emission probabilities are set to uniform distributions for the basic and decoy experiments, but for the 3′UTR experiments are fit to the data with the Baum–Welch algorithm prior to evaluation.

#### Motif analysis for synthetic data

We performed MD with a selection of tools, including eight published methods and all six objective functions currently implemented in Discrover. Among the published ones are two signal-only MD methods, BioProspector and MDscan, as well as six discriminative ones, CMF, DECOD, DME, DREME, FIRE and MoAn. In all cases, we used the tools for each data set to discover the most discriminative motif of length 8 nt and report its occurrences. The method of (37), YMF and ALSE were excluded respectively because the source code is not publicly available, could not be retrieved or failed to compile. We tried to evaluate the performance of Dispom, DEME and DIPS, but found them to run prohibitively slow for application to the larger sequence data sets, see Supplementary Table T4. The default number of iterations MoAn uses made it infeasible to evaluate performance on the decoy data set. We thus reduced the number of iterations to a tenth of the default value. Bugs were found and fixed in the source code of CMF and MoAn, see supplementary text and Supplementary Figure S10.

#### Data of PUF RBP family

We retrieved various data sets for the PUF family of RBPs in different species, including *Saccharomyces cerevisia*, *Caenorhabditis elegans*, *Drosophila melanogaster* and *Homo sapiens*. DNA microarray quantifications of co-IPed mRNA (RIP-Chip) was used to define targets of Puf1, Puf2, Puf3, Puf4 and Puf5 in yeast (64), of the worm homolog FBF-1 (65), of Pumilio in adult fly ovaries (66), and of human PUM1 (67,68) and PUM2 (67) from HeLa S3 cells. Additionally, we analyze PAR-CLIP data of human PUM2 (9) from HEK293 cells.

Due to the lack of finer spatial resolution entire 3′UTR sequences were used for the array-based data. When probe sets mapped to multiple transcripts with 3′UTR sequences, the longest one was used. Yeast 3′UTRs were based on (69). Worm 3′UTRs were retrieved from WormMart in version WS220. Fly 3′UTRs were retrieved from FlyBase in version 5.48. Human 3′UTRs were retrieved from Ensembl release 70 (GRCh37.p10) and from RefSeq NCBI36.1/hg18. Human 3′UTRs for the array data were selected by Ensembl transcripts IDs and RefSeq transcript ID (67), or only by RefSeq transcript ID (68).

For the yeast analysis, binary contrasts were considered, where the 3′UTR sequences of each Puf protein's target genes served as signal set, and the controls comprised all yeast 3′UTRs not part of the signal set.

For the worm homolog FBF-1 we retrieved a table of bound target genes from the supplementary material of (65) and mapped WormBase gene IDs to transcript IDs. To set up contrasts for the FBF-1 data, we follow the analysis of (65) who split the data into 15 approximately equally sized rank groups. Thus, we split up the 3294 3′UTR sequences of target genes by rank into 14 groups of size 220 and one group of size 214.

Target genes of the fly RBP Pumilio are tabulated in the supplementary material of (66). We translated the FlyBase gene IDs of the target genes in this table to transcript IDs. 3′UTR sequence of non-target genes were used as control.

The supplementary material of (68) provides a table with log of odds (LOD) scores for binding of PUM1 to genes. Differently from the other array results that we use, this table also includes genes for which there is no evidence of binding. Thus, following (68) we used as signal data the 3′UTR sequences of genes with an LOD greater than 0, and the remainder as control.

The supplementary materials of (67) provide tables for targets bound by PUM1 and PUM2. We procured control data by taking the set of Ensembl 3′UTR sequences complementary to the bound targets tables.

As signal sequences for the PUM2 PAR-CLIP data of (9) we used read covered regions, as available from the Dorina database (70). Dinucleotide distribution conserving shuffles of signal sequences served as controls.

#### Motif analysis for PUF RBP family

Using Plasma, for each data set the most discriminative IUPAC word according to MICO was determined for each length of 7–12 nt. Using Discrover, HMMs were seeded on each of these and parameters optimized, maximizing MICO. For each RBP, we selected the motif yielding the best corrected *P*-value.

#### Data of RBP RBM10

We retrieved the GRCh37/hg19 coordinates of binding sites of two RBM10 PAR-CLIP data sets of (26) via Gene Expression Omnibus (GSM1095142 and GSM1095143). These are defined as the positions with the highest number of PAR-CLIP cross-linking-induced nucleotide conversion events (PAR-CLIP conversions) within each of the clusters. We consider all binding sites that have at least 10 PAR-CLIP conversions. For each binding site we retrieved sequences of 41 nt by adding 20 nt flanks on each side. The sequences are split into two groups: those whose central position lies in exons, and among the rest those whose central position lies in introns. Exons and introns of RefSeq protein coding genes were considered.

#### Motif analysis for RBM10

For RBM10 we performed DMD by jointly maximizing MICO across the contrasts given by the two data sets and their respective shuffles. We independently analyzed the exonic and intronic sequences. Using Plasma, we identified the three most discriminative IUPAC words for each length of 5–10 nt. We ran Discrover in multiple MD mode, using the seeds reported by Plasma, as well as 1-nt-shifted variants of each. In total, 6 × 3 × 3 = 54 seeds were considered for each analysis.

#### ChIP-Seq data

We applied our method to ChIP-Seq data of two studies of mouse embryonic stem cell (ESC) TFs (71,72). The first study performed ChIP-Seq experiments for 13 sequence-specific TFs in mouse ESC E14 (71). These include Nanog, Oct4, Sox2, Esrrb and Zfx, involved in ESC self-renewal, Klf4, c-Myc and n-Myc, which contribute to reprogramming of somatic cells to a pluripotent state (73,74), the cell cycle regulator E2f1 (75), as well as Ctcf, which insulates transcriptional domains (76), and Tcfcp2l1, which is preferentially upregulated in ESCs (77). In addition, two factors downstream of signalling pathways are included: BMP1-induced Smad1 (78) and LIF-induced Stat3 (79). The second study (72) produced additional ChIP-Seq data from mouse ESC V6.5 for Oct4, Sox2, Nanog and Tcf3, a repressor of key pluripotency gene expression (80–82). We retrieved from mm8 sequences of 101 nt centered on the midpoints of the ChIP-Seq bound regions as reported by (71,72).

#### ChIP-Seq motif analysis

The ChIP-Seq data sets were individually contrasted to dinucleotide frequency preserving shuffles of the signal sequences. For each data set we discovered multiple motifs as described in the Materials and Methods section. We performed DMD for lengths of 5–16 nt, using MICO as objective function considering the three most discriminative IUPAC words of each length and 1-nt-shifted variants of each as seeds for HMM optimization. Thus, for each data set we considered 12 × 3 × 3 = 108 seeds.

## RESULTS

### Synthetic data

The construction of synthetic data sets defines true binding sites in the sequences, and allows to classify predicted binding sites as true or false positives, see Supplementary Figure S9. Similarly, implanted binding sites that are not predicted are false negatives. Following (44,83) we quantify prediction performance both on the nucleotide and binding-site level. On nucleotide level we use the nucleotide-level MCC (nCC). On binding-site level we use the average site performance sAP, defined as the arithmetic mean of site sensitivity sSn and site positive predictive value sPPV. Note that like (44) we require 50% overlap to define a match on the site level. See the supplementary text, in particular equations (**89**)–(**92**), for definitions of nCC, sSn, sPPV and sAP.

We refer to site prediction performance when knowing the true implanted PWM as recognizability. In general, recognizability limits motif discoverability. Consequently, recognizability may serve as a reference for MD performance. Thus, we created HMMs of the implanted PWMs as described in the materials section, and used them to predict binding sites to estimate recognizability.

### High-level MD performance

**Figure 2.**
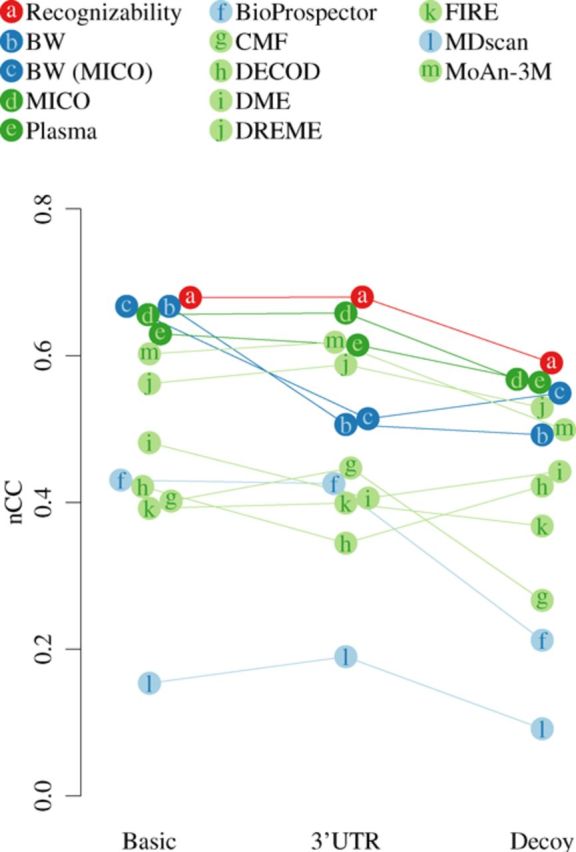
Summarized motif-finding performance of various methods on three synthetic data sets measured by the nucleotide-level MCC (nCC). Recognizability (red) serves as reference. Blue denotes signal-only motif learning methods, while green denotes discriminative MD methods. Dark letters and light background denote published motif-finding methods, light letters and dark background denote motif finding with objective functions implemented in Discrover. BW: Baum–Welch training of HMMs seeded with the most frequent IUPAC motifs of degeneracy maximally 2, BW (MICO): Baum–Welch training of HMMs seeded with IUPAC motifs maximizing MICO. Plasma: IUPAC RE motif-based seeding method of Discrover, optimizing MICO as objective function. MoAn-3M: MoAn run with 3 × 10^6^ iterations.

By summarizing across data sets the true and false site predictions, as well as true and false non-predictions, we computed high-level performance summaries. Figure 2 gives the summarized nCC for the different MD tools in the three sets of experiments, as well as the motif recognizability. Supplementary Table T5 shows the corresponding numbers, both absolute and relative to motif recognizability. Supplementary Figure S11 presents additional performance metrics, including sSn, sPPV and sAP.

Recognizability nCC indeed is higher than all methods’ nCC in all three sets of experiments. Using MICO as objective function, our HMM-based method Discrover achieves in all three sets of experiments MD performance at ≥96% of motif recognizability. Signal-only learning with the Baum–Welch algorithm (BW) achieves MD performance close to recognizability only in the basic set of experiments.

We evaluated MD performance of our seeding method Plasma in isolation, finding IUPAC RE based motifs by heuristically optimizing MICO. At 90–96% of motif recognizability, its MD performance is lower than that with subsequent HMM optimization yet still appreciably higher than that of most other methods.

To separate the influence of objective function choice in seeding from that in HMM parameter optimization, we used negative example sequences to determine seeds by MICO that were used for signal-only, generative learning of HMM parameters with the Baum-Welch algorithm (‘BW (MICO)’ in Figure 2). This alleviated some of the problems that BW has on the decoy experiments, but did not remedy those on the 3′UTR experiments.

Most of the discriminative objective functions implemented in Discrover yield comparable MD performance, with only DFREQ performing substantially worse than the others, see Supplementary Figure S12.DREME and MoAn were the best-performing published DMD methods and respectively achieved nCC of 83–90% and 85–91% relative to recognizability.

The large default number of 3 × 10^7^ iterations used by MoAn made it infeasible for us to evaluate performance on the decoy data set. Instead, we only used 3 × 10^6^ iterations, reducing the runtime by a factor of 10. Figure 2 includes the MD performance of MoAn with the reduced number of iterations. As Supplementary Figure S12 shows, MoAn's MD performance further increases on the basic and 3′UTR data sets when 3 × 10^7^ iterations are used.

With nCC of 36–75% relative to motif recognizability, the other published MD methods achieved substantially lower performance. The low performance of CMF seems to be caused by overly eagerly accepting binding sites. This is evidenced by CMF achieving the highest sSn and simultaneously the lowest sPPV over the three sets of experiments (Supplementary Figure S11). Perhaps stringent filtering of results might help solve this problem.

It should be noted that CMF, DME and MDscan only support double-stranded DNA mode, and might yield higher MD performance on these experiments by accounting for the larger RNA motif space. Initially, we evaluated the MD performance of an earlier DREME version that did not support single-strand MD to analyze RBP data. The now-current version (shown in Figure 2) supports RBP motif analysis. DREME's MD performance was unchanged by this update (Supplementary Figure S12).

Prompted by referee comments we sought to consider alternative seeding strategies for our own method. For this purpose we used DREME to discover seeds that are then further optimized with MICO by Discrover because DREME is both relatively fast (see next paragraph) and has a good MD performance. This yields performance comparable to using our seeding method Plasma but superior to that of DREME without subsequent HMM optimization (Supplementary Figure S12).

### Runtime of MD methods

We analyzed the runtime of the MD methods on the three sets of experiments (see supplementary text and Supplementary Figure S16) and found considerable variation. Our seeding method Plasma is the fastest of the considered methods, followed by DME, DREME and Discrover. Compared respectively to Plasma and Discrover, FIRE ran at least 59 and 10 times as long. CMF and DECOD ran at least 138 times as long as Plasma, and at least 24 times as long as Discrover. In spite of the reduced number of iterations, MoAn-3M still took 227–932 times as long as Plasma.

### Signal-only and discriminative learning

To illustrate the benefits of using negative examples in MD, we analyze MD performance of BW and MICO as a function of the variables controlled in the experiments, and compare to motif recognizability. For both learning approaches, models are filtered for discriminative significance based on MICO.

**Figure 3.**
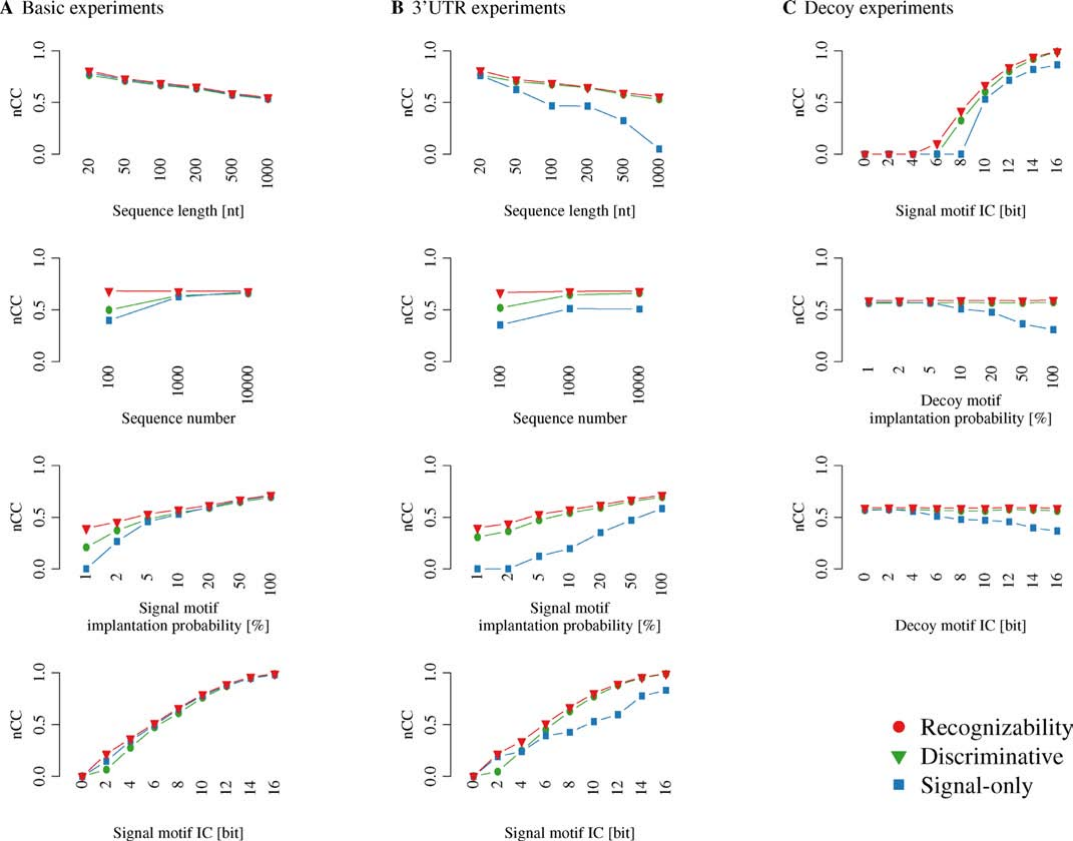
Motif recognizability and discovery performance on synthetic data in the (**A**) basic, (**B**) 3′UTR and (**C**) decoy experiments. Recognizability and discovery performance are measured by nucleotide-level MCC (nCC) as a function of different variates, summarized over the remaining variates. Recognizability (red) serves as reference. Signal-only learning (blue, BW in Figure 2) was performed with the Baum–Welch algorithm on the signal data only, and used as seeds the 8mers of degeneracy at most 2 that are most frequent in the signal data. Discriminative learning (green, MICO in Figure 2) used MICO as objective function for seed finding and HMM parameter optimization. Supplementary Figures S13–S15 give the measures sAP, sSn and sPPV.

Figure 3 displays the nCC broken down by variates, computed by summing over the other variates (sSn, sPPV and sAP in Supplementary Figures S13–S15). In the basic experiments (Figure 3A), recognizability decreases with increasing sequence context size, with decreasing implantation frequency, and with decreasing IC. Throughout most combinations of the varied parameters, the MD performance of both signal-only and discriminative learning is very close to motif recognizability. While the differences in MD performance are generally small between signal-only and discriminative learning on the basic experiments, overall they are slightly in favor of signal-only learning (Figure 2). Reductions relative to motif recognizability are seen when data are limited to 100 sequences. Increasing sequence numbers to 1000 yields a MD nCC close to motif recognizability, and MD nCC increases further when 10 000 sequences are available. Some deficits relative to motif recognizability are also seen for motifs of very low implantation frequency or very low IC.

Motif recognizability and MD performance of discriminative learning in the experiments based on real human 3′UTR (Figure 3B) are virtually identical to those in the basic experiments. The MD performance of signal-only learning is, however, negatively impacted by the higher complexity sequence background in nearly all variate combinations. Even 1000 or more sequences are not sufficient to yield nCC close to motif recognizability for signal-only learning. That nCC does not further approximate motif recognizability when 10 000 sequences are available demonstrates that generative signal-only learning is genuinely confused by the characteristics of real 3′UTR sequences.

In the decoy experiments (Figure 3C), (signal) motif recognizability varies in response to signal motif IC but is unaffected by variation of decoy motif implantation frequency or decoy motif IC. MD performance of discriminative learning is also unaffected by increasing implantation frequency or IC of decoy motifs, and generally close to motif recognizability. MD performance of signal-only learning is deteriorating in response to the increasing potential likelihood contribution of decoy motifs. As implantation frequency of the signal motif was fixed to 10% in these experiments, a phase transition is visible at decoy motif implantation frequency 10% between little or no, and strong negative influence of the decoy motif on the discovery performance of signal-only learning. Similarly, decoy motifs of low IC do not strongly affect signal-only learning's MD performance, while higher IC decoy motifs lead to reduced MD performance.

When results are not filtered using discriminative significance based on MICO, the MD performance of signal-only learning deteriorates substantially, while for discriminative learning it decreases only slightly (Supplementary Figure S17).

### Discriminative motifs of the PUF RBP family

After benchmarking our method on synthetic data, we sought to confirm its utility for real biological data. We thus applied DMD with MICO as objective function on the PUF RBP family data sets. The results are summarized in Table 3, and details are presented in supplementary table T6A.

**Table 3.**
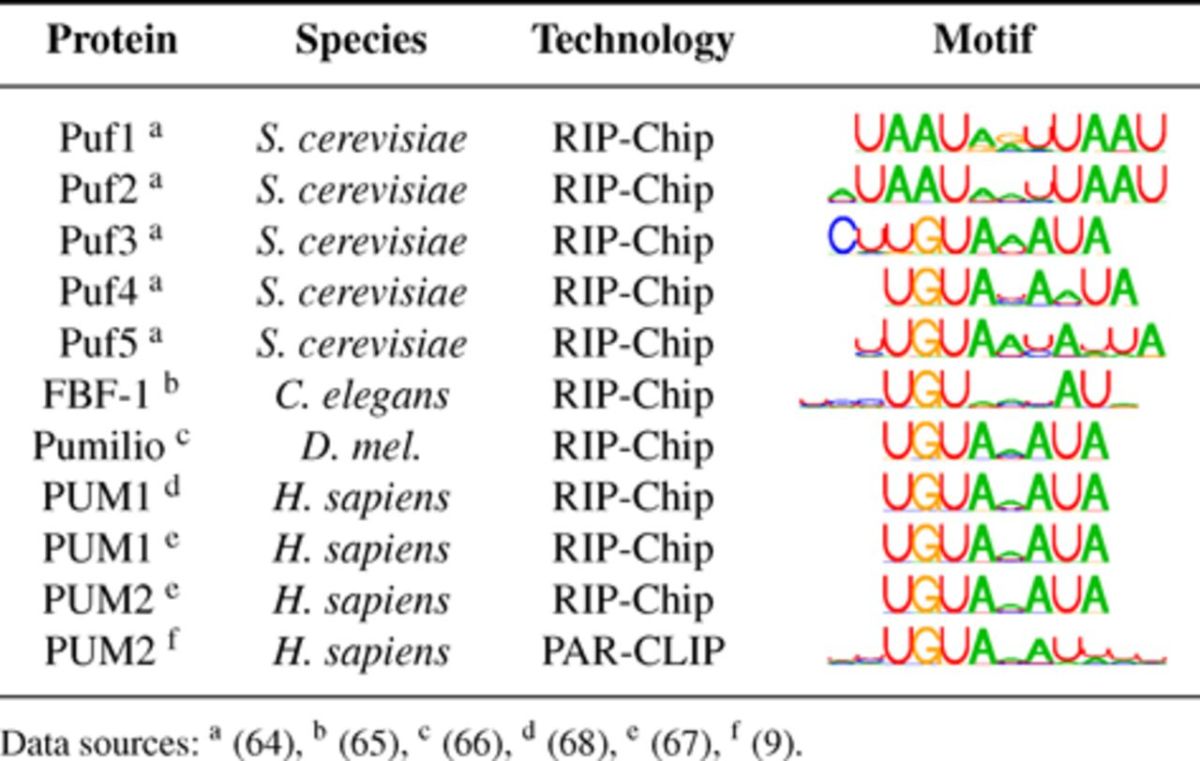
Discriminative motif analysis of the PUF family RBPs with MICO as objective function. Motifs of 7–12 nt selected by *P*-value.

All identified motifs resemble the respective published motifs and are, except for Puf1 and Puf2, similar to the PUF recognition element (PRE) with IUPAC motif representation UGUAHAUA. Most conserved is the specificity of the first four positions, but variability is seen in the second part, and in the context. Puf1 and Puf2 have a motif very unlike that of the other family members. Puf3 shows preference for a C two positions upstream. Puf4 appears to favor a 9-nt variant, and Puf5 a 10-nt variant. Also, FBF-1 appears to favor a 9-nt variant of the motif.

Except for Puf1, all motifs are significantly discriminative according to the corrected *P*-value (Supplementary Table T6A). While for Puf1 the previously reported motif is found with considerable relative enrichment of 40.8% signal over 0.7% control sequences that have at least one motif occurrence, the observed enrichment is insufficient to meet the significance threshold as there are only 32 signal sequences in this data set.

Discriminative analysis of the data sets of the fly Pumilio and of human PUM1 and PUM2 array data uniformly yield UGUAHAUA as most discriminative motif, while analysis of the PUM2 PAR-CLIP data yields a more diffuse affinity toward A/U on the second motif half. We investigated this disparity between our analyses of RIP-Chip and PAR-CLIP data (see supplementary text and Supplementary Figures S18–S26), and concluded that it is due to differences between the technologies, with PAR-CLIP's higher spatial resolution allowing a more fine-grained analysis of the spectrum of recognized words, including low-affinity variants.

### Discriminative motifs of RBM10

While earlier results allowed to validate the PUF motifs, we next applied our method to data not previously analyzed. For this we chose PAR-CLIP data of the alternative splicing factor RBM10. This revealed three motifs for the exonic sequences, and two for the intronic ones, see Supplementary Table T7. The motif 
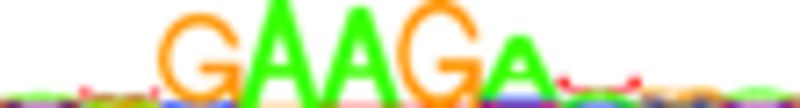
, a known exonic splicing enhancer (ESE) signal (84,85), is the most differential one within the exonic sequences. Occurrence of the motif is positively correlated with the number of PAR-CLIP conversions in the sequence (Supplementary Figure S27), with the motif occurring in ≥30% of the sequences with most conversions. The most differential motif in the intronic sequences, 
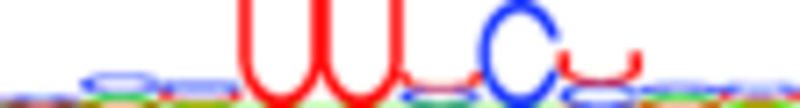
, resembles the signal of the polypyrimidine tract. Unlike the ESE motif in the exonic sequences, however, this motif is negatively correlated with PAR-CLIP conversions (Supplementary Figure S28). An infrequently occurring third motif discovered in the exonic sequences is reverse-complementary to the ESE motif, polypyrimidine-rich like the one found in the intronic sequences, and also negatively correlated with PAR-CLIP conversions. The second motif discovered in the intronic sequences is a previously undescribed, palindromic 9mer, whose occurrence is positively correlated with PAR-CLIP conversions.

We considered 94 5mers reportedly enriched in RBM10 CLIP-Seq data (86) and determined their frequency in the PAR-CLIP data (Supplementary Tables T8 and T9, summarized in Supplementary Table T10). About half of them are less or equally frequent in the PAR-CLIP sequences compared to shuffled controls. Only <15% are significantly enriched in the PAR-CLIP data, and these are consistent with the motifs our MD reported.RBM10 has four RNA-binding domains, among them a RanBP2-type zinc finger domain that binds *in vitro* to single-stranded RNA with the sequence AGGUAA (87,88), which is almost identical to the conserved consensus sequence of metazoan 5′ splice sites (89,90). Two exons affected by RBM10 knock-down carry such 5′ splice sites (91). We counted the number of sequences in the PAR-CLIP data that have at least one occurrence of these words (Supplementary Table T11). The resulting numbers are extremely low (<1% of sequences), in spite of our respecting all word occurrences and not just those that overlap 5′ splice sites. We found the 5′ splice site-like motifs not to be enriched in the PAR-CLIP sequences compared to shuffled sequences.

### Discriminative motifs in mouse ESC ChIP-Seq data

Having considered synthetic and RBP data, we conclude our case studies by applying Discrover to ChIP-Seq data of mouse ESC and related TFs published by (71,72). By contrasting to shuffled sequences, we discovered for each data set one or more motifs (Table 4, Supplementary Table T12). In most cases the assayed TF's motif is the top motif and the spatial distribution of occurrences of the top motif is highly enriched around the sequence midpoints (Supplementary Table T13). Generally, where motifs are identified in multiple data sets, they are highly consistent. Notably, almost all of the discovered motifs are identifiable as previously known motifs (TOMOTM q-value ≤ 5% (92)). Frequently, motifs of one of the other assayed TFs are discovered as secondary motifs. Co-discovery of the motifs of the ESC TFs Klf, Oct4, Sox2 and Zic is particularly striking. Consistent with earlier reports (39,71,93), Discrover does not find previously described cognate motifs for E2f1 and Smad1 in the respective ChIP-Seq data.

**Table 4.**
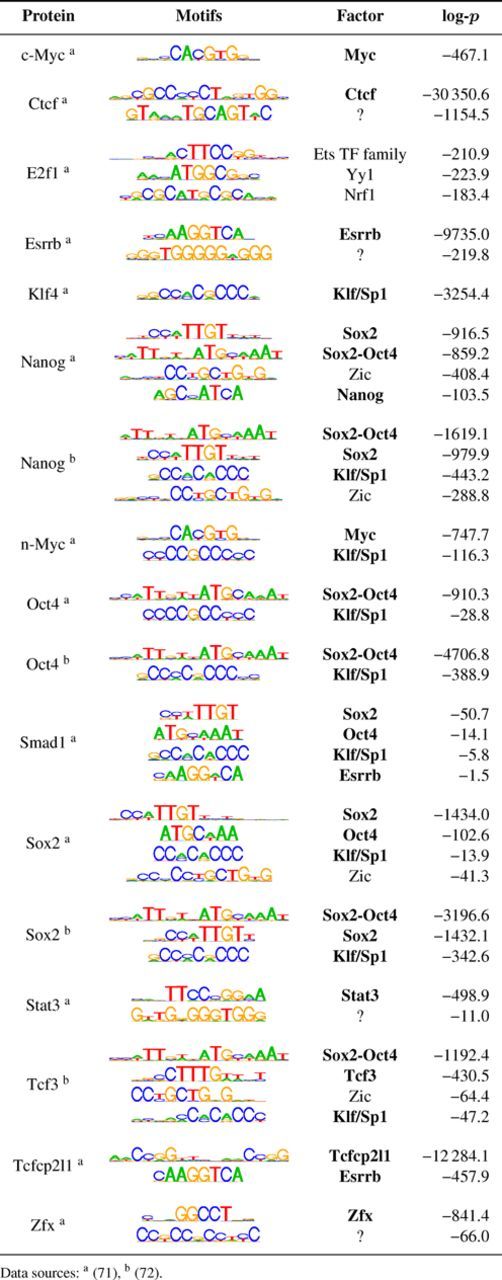
Discriminative motif analysis of mouse ChIP-Seq data. Protein: ChIP'd protein. Motifs: one or more motifs discovered in the ChIP'd protein's sequences. Factor: TF (family) known to bind the discovered motif (TOMTOM *q*-value ≤ 0.05), bold if one of the ChIP'd proteins. log-*p*: MICO log-*p* value.

For Nanog and Tcf3 the most discriminative motifs against shuffled sequences are not the cognate motifs but those of Sox2 and Oct4. Extending earlier analyses (39), we contrasted the Nanog and Tcf3 sequences to other factors’ ChIP-Seq sequences highly enriched for the Sox2 and Oct4 motifs. These analyses yield in 15/16 cases the cognate motifs of Nanog and Tcf3 (Supplementary Table T14). Only Nanog (72) versus Oct4 (71) yields another motif: the Sox2 monomer motif is more discriminative across this contrast than the cognate motif.

The DREME publication (39) analyzed 13 of the 17 data sets studied here and reported more motifs than discovered with our method. Exemplarily, we investigated the differences of Discrover and DREME analyses of the Oct4 (71) data, also including FIRE into this comparison. We generated two additional sets of shuffled sequences, and applied the methods on the three contrasts. Supplementary Table T15 lists the results. Discrover consistently reports the full-length Sox2-Oct4 heterodimer motif and the Klf/Sp1 motif. DREME finds 18–21 IUPAC RE motifs and FIRE 13–16, of which 8–10 are enriched in the signal sequences. Both DREME and FIRE are designed for the discovery of short motifs, and respectively 8–10 and 3–6 of their motifs are variants of partially overlapping segments of the Sox2-Oct4 heterodimer pattern. DREME also consistently finds the Esrrb and Myc motifs, while FIRE finds the Esrrb motif in two of three analyses. Other motifs found by DREME and FIRE are not identifiable as known motifs, or are not reproduced for different sets of shuffled controls.

## DISCUSSION

Generating synthetic data allows to evaluate performance in a supervised manner, as true and false motif occurrences are trivially defined in the data-generation process. In addition, control over the parameters determining the synthetic data-generating process allows for controlled experiments of the sensitivity of the method with respect to (w.r.t.) important variables.

By varying the number of sequences available for learning we studied how much data is necessary to saturate MD performance. For all methods we found substantially increased MD performance when going from 100 to 1000 sequences. With 10 000 sequences MD performance further approached the limit of motif recognizability.

By varying sequence context size and implantation frequency we studied the sensitivity of MD w.r.t. signal preponderance. We found MD performance to react approximately linearly to logarithmic changes of preponderance. This observation is in line with theoretical expectations based on a simple log-odds based inference scheme in which the negative logarithm of the (position-wise) occurrence frequency acts as cutoff on a PWM score.

Control of IC allows to determine the signal/noise ratio of binding-site predictions. Among the parameters we varied, IC of the true motif is found to be the most important determinant of motif recognizability, and thus of the limit of MD performance. Motif-finding performance shows a non-linear response to variation of IC, with a sigmoidal contribution due to the response of sensitivity (Supplementary Figure S14).

Observations regarding the sensitivity of MD performance w.r.t. to one parameter are based on averaging over the values of other parameters, and the additional gains from increased sequence numbers are larger in more difficult learning problems. Thus, e.g. saturation of MD performance with increasing number of sequences occurs earlier for frequent, high-IC motifs, and later for infrequent, low-IC motifs.

The observations also hinge on the choices for constant parameters of data generation. For example, motifs of 8-nt length model typical RBP binding sites. However, as exemplified by the analyzed ChIP-Seq data, many DBPs recognize longer motifs. Also, the choice of PWM to simulate motif occurrences merits discussion. While being much used in the field of sequence analysis, PWMs disregard the possibility of dependent emission probabilities at different positions, and thus need not be good approximations to real binding-site patterns (94).

### 

#### Signal-only and discriminative MD

When the true model is used—as in the basic synthetic data experiments—generative, signal-only learning was optimal, and in these cases discriminative learning was nearly as good. But in situations involving slight model mis-specification—as in the 3′UTR and decoy experiments, and likely the general case—discriminative learning discovered motifs more robustly than generative, signal-only learning.

### Comparison to published discriminative motif finders

Our HMM-based method Discrover achieved the highest MD performance of all considered methods in the synthetic data experiments. The second-best MD performance was found for our seeding method Plasma. The best published DMD tools were DREME and MoAn with reduced number of iterations. At the default number of iterations, MoAn achieved an even higher MD performance on the basic and 3′UTR data sets, only surpassed by Discrover. DREME is an RE-based MD method and performed consistently better than CMF, DECOD and DME, which are all based on PWMs. This shows that RE-based sequence specificity models are not necessarily inferior to probabilistic ones when different objective functions and optimization procedures are used. Conversely, while FIRE uses the same objective function as Discrover, its MD performance is much lower, demonstrating that aside from the objective function also other properties of MD tools are important. The low MD performance of the signal-only methods, BioProspector and MDscan, underlined the utility of using negative examples for MD.

#### Runtime of MD methods

Despite the reduced number of iterations, MoAn was the slowest of the considered methods, running at least 40 times as long as Discrover. Among the well-performing, published methods only DREME had a runtime lower than Discrover, while our seeding method Plasma was the fastest method overall.

We intended to evaluate several further discriminative tools, including DEME, DIPS and Dispom. However, these had runtimes higher than any of the MD methods considered here, making it impractical for us to evaluate their performance.

### Analysis of PUF RBP family data

Our framework reproduced previous findings regarding the sequence specificity of the well-studied PUF RBP family, using data from various species and different technologies. This application also showcased the usage of multiple kinds of contrasts, including comparison of bound genes versus unbound ones, of bound genes versus the genomic complement, of multiple groups of genes ranked by binding evidence, as well as the comparison of signal to shuffled sequences. Furthermore, our analyses revealed the relevance of low-affinity variants not conforming to the PRE UGUAHAUA. This was possible due to two factors: the finer spatial resolution of PUM2 PAR-CLIP data and the inclusion of lower-ranking sequences into our analysis.

### Analysis of RBM10 data

Our analysis of PAR-CLIP data for the alternative splicing regulator RBM10 yielded motifs implicated in splicing regulation. The most differential motif in the exonic PAR-CLIP clusters is a known ESE signal, while the intronic one resembles the signal of the polypyrimidine tract.

The ESE motif has been reported to be bound by SFRS1 (8,95–97) and by eIF4AIII (98). RBM5, a splicing factor related to RBM10, is known to compete for binding to the polypyrimidine tract with U2AF65 (99). Intriguingly, the polypyrimidine tract binding protein, PTB, has been reported to bind to the double-stranded region of a secondary structure motif in the form of a hairpin whose one arm consists of pyrimidine-rich sequence, while the other consists of purine-rich sequence (100). It is conceivable that similar secondary structure might also be of importance to the regulation exerted by RBM10, which could either favor or disfavor the formation of such hairpins and influence splicing through this mechanism.

#### Relation to previous findings

Analysis of the PAR-CLIP data did not corroborate RBM10's previously reported specificity either for motifs similar to 5′ splice sites, or for most motifs reported to be enriched in CLIP-Seq data. RBM10 has four RNA-binding domains: two RRM domains, and RanBP2- and C2H2-type zinc finger domains. Thus, RBM10 may possess complex RNA-binding properties, and our negative results for most previously reported motifs need not be in conflict with earlier analyses.

Although both the purine-rich and the pyrimidine-rich motifs found by Discrover in the PAR-CLIP data are included in the motifs reported by Bechara *et al.*, it seems our findings are first in underlining their central importance for RBM10 binding, as most of the 94 words reported by Bechara *et al.* are not enriched in PAR-CLIP data. Bechara *et al.* do not draw particular attention to the motifs highlighted here, and instead follow up on other motifs that lack—as shown here—statistical evidence for enrichment in PAR-CLIP data (86).

#### Correlation with PAR-CLIP conversions

The number of PAR-CLIP conversions in a given cluster results primarily from presence and affinity of contained binding sites, but also further effects, including transcript abundance and sequence composition (9,101,102). Thus, the positive correlation with PAR-CLIP conversions suggests that RBM10 binds with high affinity to the purine-rich motif. Conversely, the negative correlation with PAR-CLIP conversions of the pyrimidine-rich motif could indicate that the pyrimidine-rich motif is bound with lesser affinity, perhaps by a different domain of RBM10; or it could be indirectly bound by RBM10 due to involvement of RNA secondary structures or interacting proteins.

#### Interpretation

In summary, based on our motif analyses, two mechanisms might be responsible for the reported exon-skipping mediated by RBM10 (26,86): (i) competition of RBM10 with splicing enhancers for the ESE motif and (ii) competition of RBM10 with U2AF65 for binding to the polypyrimidine tract—either through RNA secondary structure or via co-factors.

### Analysis of ChIP-Seq data

By discriminative learning using MICO as objective function we successfully rediscovered previously reported sequence motifs for the analyzed ChIP-Seq data. Using contrasting information obviated the need to apply repeat masking or other kinds of filtering to preprocess the data, and DMD was applied directly to the ChIP-Seq bound regions, leveraging the full size of these data sets of up to 39 609 signal sequences. The Discrover analyses of ChIP-Seq data appear to be stringent and robust, as indicated by (i) the similarity of multiply discovered motifs, (ii) the high proportion of previously described motifs recovered, (iii) the high proportion of known co-factor motifs among the previously described motifs and (iv) the consistent results when applied to multiple sets of shuffled sequences.

Our comparison of Discrover, DREME and FIRE DMD results on Oct4 data showed that DREME and FIRE yield more motifs than Discrover, and that these motifs may include presumed-true co-factor motifs that are not identified by Discrover. However, the motifs yielded by the RE-based methods are short, redundant and contain motifs that are either not known to be bound by stem cell co-factors, or that are not reproduced in multiple runs with different sets of shuffled controls. Thus, while potentially missing some true motifs, Discrover consistently and robustly identifies a non-redundant set of full-length motifs with a higher true-positive rate.

## CONCLUSION

We presented a novel MD method that integrates different signal-only and discriminative objective functions. The engineering aspects of the software allow analysis of genome- and transcriptome-scale data. Using synthetic data we compared the merits of different objective function choices within our framework and across published MD tools. Our IUPAC RE-based seeding method Plasma achieved higher MD performance than most published methods, while being faster than all other methods. Seeding HMMs with Plasma motifs and optimizing with Discrover yielded the highest observed MD performance, while still being faster than the best-performing published methods except for DREME. Application to TF and RBP data proved our method's utility to analyze real biological data for the study of transcriptional, post-transcriptional and splicing regulation, and provided new insights for the sequence-binding specificity of the alternative splicing regulator RBM10.

Mutual information was introduced to quantify the capacity of noisy signal transmission channels in communications theory (52). The application to MD in the form of MICO suggests to conceive of strands of nucleic acids as information transmitting channels. In essence, enhancer and promoter regions mediate inherited control information to specialized receptors: sequence-specific DNA-binding TFs. Similarly, stretches of RNA molecules transmit control information to RBPs. Nucleic acid binding proteins sample their respective channels, and, upon discovering their cognate signals in the nucleic acid patterns, these regulatory proteins bind and thereby initiate the execution of their regulatory purpose.

## SUPPLEMENTARY DATA

Supplementary Data are available at NAR Online.

SUPPLEMENTARY DATA
