# Supplementary material for: Binding site discovery from nucleic acid sequences by discriminative learning of hidden Markov models
Source: Nucleic Acids Res. 2014 Nov 11;42(21):12995–3011. doi: 10.1093/nar/gku1083 (PMC4245949; doi:10.1093/nar/gku1083)
Supplement: SUPPLEMENTARY DATA [file supp_42_21_12995__index.html]

Binding site discovery from nucleic acid sequences by discriminative learning of hidden Markov models — SUPPLEMENTARY DATA 

# Binding site discovery from nucleic acid sequences by discriminative learning of hidden Markov models

## SUPPLEMENTARY DATA

**Files in this Data Supplement:**

- SUPPLEMENTARY DATA
