# Supplementary material for: Binding site discovery from nucleic acid sequences by discriminative learning of hidden Markov models
Source: Nucleic Acids Res. 2014 Nov 11;42(21):12995–3011. doi: 10.1093/nar/gku1083 (PMC4245949; doi:10.1093/nar/gku1083)
Supplement: SUPPLEMENTARY DATA [file supp_gku1083_supplement.pdf]

Published online November 11, 2014 *doi: 10.1093/nar/gku1083* **1–56**

Supplementary material: Binding site discovery from nucleic acid sequences by discriminative learning of hidden Markov models

Jonas Maaskola* **and Nikolaus Rajewsky**

Laboratory for Systems Biology of Gene Regulatory Elements, Max-Delbrück-Center for Molecular Medicine, Robert-Rössle-Strasse 10, Berlin-Buch 13125, Germany

ABSTRACT

This supplementary text provides details of the method presented in the main manuscript, as well as supplementary results. It discusses mathematical details of measures of association to elicit sequence motifs with discriminative learning. The gradients of those objective functions are given that are used in the gradient optimization of the HMM parameters. Furthermore, in addition to the mathematical formalism of HMMs, in particular of binding site HMMs, we describe how the gradient of the HMM likelihood with respect to transition and emission parameters are computed. Multiple testing correction in motif analysis is also discussed. We explain the multiple motif discovery mode of Discrover, and illustrate the Discrover module for the bioinformatics web framework Galaxy.

Supplementary results presented here include detailed motif discovery performance metrics on synthetic data for all considered motif discovery results. For data of the PUF family of RBP this supplement includes a dilution analysis, as well as word-based analyses. For the ChIP-Seq data positional distributions of the motif occurrences in the ChIP-Seq regions are displayed. Also, tables with detailed numbers of occurrences of motifs in signal and control data are included for both RBP data and for ChIP-Seq data.

MEASURES OF ASSOCIATION

Here we collect various discriminative objective functions, some of which are implemented in the accompanying software, while others are used by related methods. Among the objective functions are measures of association in contingency tables, as well as measures not based on contingency tables. Some of these objective functions are directional, i.e. motifs that maximize them are not only differential but in fact enriched in the signal sequences. By considering motifs that minimize directional objective functions one can identify differential motifs that are depleted in the signal sequences. For non-directional objective functions it is possible to filter differential motifs for enrichment in the desired sample.

Supplementary table T1. 2×2 contingency table of number of sequences in datasets for two conditions that have or do not have at least one occurrence of a motif. TP and FP stand for true and false positives, TN and FN for true and true negatives.

Condition	Motif present	Motif absent
Signal	TP	FN
Control	FP	TN

2×2 CONTINGENCY TABLES BASED MEASURES

First we will discuss measures that are based on 2×2 contingency tables, as exemplified in supplementary table T1. Such contingency tables are applicable in binary classification problems on data representing contrasts involving a pair of positive and negative example sets.

Difference of relative frequency - DFREQ

The simplest directional measure of association is the *difference of relative frequency* of motif prevalence in the signal and control data (DFREQ),

$$\text{DFREQ} = \Delta F = \frac{TP}{TP+FN} - \frac{FP}{FP+TN}. \quad (1)$$

One may argue that a possible drawback of this measure is the failure to assign higher relevance to qualitative differences between signal and control samples. As an example, consider the following two contingency tables,

$$T_1 = \begin{pmatrix} 1000 & 0 \\ 500 & 500 \end{pmatrix} \quad \text{and} \quad T_2 = \begin{pmatrix} 950 & 50 \\ 450 & 550 \end{pmatrix}.$$

For both T_1 and T_2 the relative frequency difference $\Delta F = \frac{1}{2}$, but there is a qualitative difference in that the data of T_1 are

*To whom correspondence should be addressed. Email: jonas@maaskola.de

© The Author(s) 2014. Published by Oxford University Press on behalf of Nucleic Acids Research.
This is an Open Access article distributed under the terms of the Creative Commons Attribution License (http://creativecommons.org/licenses/by/4.0/), which permits unrestricted reuse, distribution, and reproduction in any medium, provided the original work is properly cited.

Supplementary table T2. $k\times 2$ contingency table of number of sequences with and without a feature in datasets of k conditions. m_i gives the number of sequences with motif in the regulatory sequences of condition i. n_i is the number of sequences in condition i.

Condition	Motif present	Motif absent
1	m_1	n_1-m_1
2	m_2	n_2-m_2
⋮	⋮	⋮
k	m_k	n_k-m_k

consistent with the hypothesis $FN=0$, while there is evidence contradicting this hypothesis for T_2.

Matthews correlation coefficient - MCC

Another directional measure of association is *Matthews correlation coefficient* (1) (MCC), defined as

$$\text{MCC}=\frac{TP\cdot TN-FP\cdot FN}{\sqrt{(TP+FP)(TP+FN)(FN+TN)(FP+TN)}}. \quad \textbf{(2)}$$

As the name indicates, the MCC is a proper measure of correlation, i.e. it takes on values between -1 and 1, where a value of 1 indicates perfect correlation, a value of -1 an inverse perfect correlation, and a value of 0 statistical independence.

The MCC of the matrices T_1 and T_2 is 0.577 and 0.546, respectively, demonstrating that the MCC assigns higher relevance to the association observed in the case of T_1.

Fisher's exact test

Another widely used measure of association on 2×2 contingency tables is *Fisher's exact test* (2). It is based on the tail probabilities of the hypergeometric distribution. Fisher's exact test amounts to summing the probabilities of all contingency tables that are more extreme than the observed contingency table. It thus gives the probability of observing a contingency table as extreme or more so than the observed one based on a null model of independence of rows and columns under fixed marginals.

For T_1 and T_2 Fisher's exact test gives an odds ratio of ∞ and 23.17, respectively, which both correspond to p-values less than the smallest representable positive floating point number. When adding one pseudo-count before computing Fisher's exact test, the resulting odds ratios are 984.59 and 22.73, respectively. There exist generalizations of Fisher's exact test to contingency tables larger than 2×2 (3).

$K\times 2$ CONTINGENCY TABLES BASED MEASURES

In case multiple positive or negative example sets are available, or in case a signal grading contrast is used, contingency tables of motif occurrence in the sequence data of n conditions take the form depicted in supplementary table T2.

Note that for probabilistic motif models $\boldsymbol{\theta}$ we will use contingency tables that hold expected counts $m_i(\boldsymbol{\theta})$ of sequences with motif occurrence in condition i.

Normalized enrichment scores

The first $k\times 2$ contingency table based measure that we want to discuss here are *normalized enrichment scores*. These are usable when the contrast provides one set of positive example sequences and multiple sets of control sequences. They are related to the difference of relative frequency discussed above. Normalized enrichment scores divide the difference of relative frequency in the signal data and the mean of relative frequencies in the control by a standard deviation computed from the relative frequencies in the control datasets. For this we define the relative frequency of sequences with motif occurrences in condition i to be $f_i=\frac{m_i}{n_i}$. Assume that condition 1 is the signal dataset, and conditions 2 to $l+1$ are control datasets. The mean relative frequency of sequences with motif occurrences in the control data is labeled $\mu=\frac{1}{l}\sum_{i=2}^{l+1}f_i$, and the standard deviation of relative frequencies in the control datasets, σ, is given by $\sigma^2=\frac{1}{l-1}\sum_{i=2}^{l+1}(f_i-\mu)^2$. Then the normalized enrichment score z is given by

$$z=\frac{f_1-\mu}{\sigma}. \quad \textbf{(3)}$$

The following measures of association are applicable to general $n\times k$ contingency tables.

Pearson's χ^2 test for independence

Perhaps the most well known such measure is given by *Pearson's χ^2 test for independence* (4). Given a contingency table with counts O_{ij} in the cell in row i and column j, and defining the row sums $R_i=\sum_{j=1}^{k}O_{ij}$ and column sums $C_j=\sum_{i=1}^{n}O_{ij}$, as well as the expected counts under the independence hypothesis $E_{ij}=\frac{R_iC_j}{N}$, where $N=\sum_{i=1}^{n}R_i=\sum_{j=1}^{k}C_j$, then the X^2 statistic is given by

$$X^2=\sum_{i=1}^{n}\sum_{j=1}^{k}\frac{(O_{ij}-E_{ij})^2}{E_{ij}}. \quad \textbf{(4)}$$

This statistic X^2 is asymptotically distributed like χ^2 with $(n-1)\cdot(k-1)$ degrees of freedom, and thus p-values are available for it.

Mutual information of condition and motif occurrence - MICO

Assume we are given a contingency table for the number of sequences with or without at least one occurrences of a motif $\mathcal{M}$ across the conditions of a contrast C. *Mutual information* of condition C and motif occurrence $\mathcal{M}$ (MICO) is an non-directional measure of association from information theory (5–7), which measures in bits the expected log odds ratio of the observed contingency table to an independence model

that assumes no association between conditions and motif presence.

$$\mathrm{MICO} = I(C;\mathcal{M}) = \sum_{\substack{c\in C\\ m\in\mathcal{M}}} P(c,m)\log_2 \frac{P(c,m)}{P(c)P(m)}, \tag{5}$$

where $P(c,m)$ are joint relative frequencies of contingency tables like depicted in supplementary table T2, and $P(c) = \sum_{m\in\mathcal{M}} P(c,m)$, and $P(m) = \sum_{c\in C} P(c,m)$ are their row and column marginal relative frequencies, respectively. In terms of the variables given in supplementary table T2 this is

$$\begin{aligned} I(C;\mathcal{M}) = \frac{1}{\sum_j n_j} \Bigg(& \sum_{i=1}^{k} m_i(\boldsymbol{\theta}) \log_2 \frac{m_i(\boldsymbol{\theta})}{n_i \sum_j m_j(\boldsymbol{\theta})} \\ & + \sum_{i=1}^{k} (n_i - m_i(\boldsymbol{\theta})) \log_2 \frac{n_i - m_i(\boldsymbol{\theta})}{n_i \sum_j \left(n_j - m_j(\boldsymbol{\theta})\right)} \Bigg) \\ & + \log_2 \sum_j n_j. \end{aligned} \tag{6}$$

Mutual information is closely related to the likelihood ratio statistic Λ (8) and the G-test statistic (9),

$$G = -2\log\Lambda = 2\log 2 \cdot I(C;\mathcal{M}) \cdot \sum_j M_j. \tag{7}$$

Due to Wilks' theorem (10), the G-test statistic for $k\times 2$ contingency tables is distributed like χ^2 with $k-1$ degrees of freedom, where k is the number of objects in the contrast. This connection allows the calculation of p-values for mutual information.

MEASURES NOT BASED ON CONTINGENCY TABLES

Difference of log likelihood

A directional discriminative objective function that is not based on contingency tables is the *difference of log likelihood* between signal and control (DLOGL),

$$\begin{aligned} \mathrm{DLOGL} = \Delta\log\mathcal{L} &= \log P\left(\boldsymbol{X}_{\text{signal}}|\boldsymbol{\theta}\right) - \log P(\boldsymbol{X}_{\text{control}}|\boldsymbol{\theta}) \\ &= \sum_{i\in\text{signal}} \log P(\boldsymbol{X}_i|\boldsymbol{\theta}) - \sum_{i\in\text{control}} \log P(\boldsymbol{X}_i|\boldsymbol{\theta}), \end{aligned} \tag{8}$$

where $\boldsymbol{X}_{\text{signal}}$ and $\boldsymbol{X}_{\text{control}}$ are the signal and control data, respectively, and $\boldsymbol{\theta}$ are the parameters of a probabilistic model. In words, this objective functions identifies models for which the signal data appear as typical examples but simultaneously the control data appear as unlikely examples. Thus, data

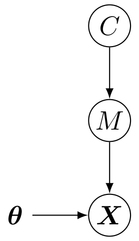

Supplementary figure S1. The graphical model of MMIE. C represents the class, or dataset, M the motif presence, and $\boldsymbol{X}$ the observed sequence. For an explanation of the graphical model notation see e.g. (11).

yielding high likelihood for a model selected by $\Delta\log\mathcal{L}$ tends to indicate signal data.

The choice of $\Delta\log\mathcal{L}$ as objective function for learning necessitates balancing of the sizes of signal and control data. In case the control dataset is considerably larger, any gain in likelihood for the signal data may be outweighed by a loss of likelihood for the control data. Dominating control data sizes may thus lead to parameters that are determined primarily by being bad generative models for the control data. Conversely, in case the control dataset is considerably smaller, this objective function is dominated by the signal likelihood and loses it discriminative character.

Difference of entropy rates

A related approach, that we did not pursue further, but that might obviate the need to balance signal and control dataset sizes, is to scale the log likelihoods by the data size, effectively computing entropy rates, and then to consider the difference of entropy rates of signal and control.

Classification probability - MMIE

We now turn to consider a probabilistic model of classes. For this we assume that the data are given in form of paired sets of sequences $\boldsymbol{X} = (\boldsymbol{X}_i)$ with corresponding classes $\boldsymbol{c} = (c_i)$. one may consider the *probability of correctly classifying all samples* (MMIE),

$$P(\boldsymbol{C} = \boldsymbol{c}|\boldsymbol{X},\boldsymbol{\theta}) = \prod_i P(C_i = c_i|\boldsymbol{X}_i,\boldsymbol{\theta}), \tag{9}$$

or its logarithm,

$$\log P(\boldsymbol{C} = \boldsymbol{c}|\boldsymbol{X},\boldsymbol{\theta}) = \sum_i \log P(C_i = c_i|\boldsymbol{X}_i,\boldsymbol{\theta}). \tag{10}$$

It may appear tempting to identify the classes with motif presence or absence, but this turns out to be problematic when the data includes mislabeled samples, in particular false positives. As this situation appears to be common in real biological data, it makes sense to consider alternatives. One possibility is to add a mixture model as follows.

The model comprises three random variables, $\boldsymbol{X}$ for the sequence, a binary variable M for the motif presence in a sequence, and a discrete variable C for the class of the sequence. Additionally, there are parameters $\boldsymbol{\theta}$ for a

probabilistic (sub-) model that determines $P(\boldsymbol{X}|\boldsymbol{\theta})$. The structure of the model is as depicted in supplementary figure S1, which corresponds to the following factorization,

$$P(C,M,\boldsymbol{X}|\boldsymbol{\theta}) = P(\boldsymbol{X}|M,\boldsymbol{\theta})P(M|C)P(C). \tag{11}$$

The conditional probabilities $P(M|C)$, the prior $P(C)$, as well as the HMM parameters $\boldsymbol{\theta}$ are parameters of the model. $P(C)$ is a probability distribution over $k=|C|$ classes, and thus represents $k-1$ free parameters. $P(M|C)$ is a table of conditional probabilities with $k\times 2$ entries, and k free parameters. Given an expression for the likelihood $P(\boldsymbol{X}|\boldsymbol{\theta})$ and for posterior probability of a feature occurrence $P(M|\boldsymbol{X},\boldsymbol{\theta})$, we then have

$$\begin{aligned} P(C,M,\boldsymbol{X}|\boldsymbol{\theta}) &= \frac{P(\boldsymbol{X},M|\boldsymbol{\theta})}{P(M)}P(M|C)P(C) \\ &= \frac{P(M|\boldsymbol{X},\boldsymbol{\theta})P(\boldsymbol{X}|\boldsymbol{\theta})}{P(M)}P(M|C)P(C), \end{aligned} \tag{12}$$

where $P(M)=\sum_{c\in C}P(M,c)=\sum_{c\in C}P(M|c)P(c)$. The likelihood of motif presence and class, $P(C,M|\boldsymbol{X},\boldsymbol{\theta})$, is then given by

$$\begin{aligned} P(C,M|\boldsymbol{X},\boldsymbol{\theta}) &= \frac{P(M|\boldsymbol{X},\boldsymbol{\theta})P(\boldsymbol{X}|\boldsymbol{\theta})}{P(M)P(\boldsymbol{X}|\boldsymbol{\theta})}P(M|C)P(C) \\ &= \frac{P(M|\boldsymbol{X},\boldsymbol{\theta})}{P(M)}P(M|C)P(C). \end{aligned} \tag{13}$$

By summing over $M\in\{m,\neg m\}$ we can express the posterior probability of classifying data $\boldsymbol{X}$ as class C given the HMM parameters $\boldsymbol{\theta}$, $P(C|\boldsymbol{X},\boldsymbol{\theta})$, as follows,

$$P(C|\boldsymbol{X},\boldsymbol{\theta}) = P(C,m|\boldsymbol{X},\boldsymbol{\theta}) + P(C,\neg m|\boldsymbol{X},\boldsymbol{\theta}) \tag{14}$$

$$= P(C)\left(\frac{P(m|\boldsymbol{X},\boldsymbol{\theta})}{P(m)}P(m|C) + \frac{P(\neg m|\boldsymbol{X},\boldsymbol{\theta})}{P(\neg m)}P(\neg m|C)\right) \tag{15}$$

$$= P(C)\left(\frac{P(m|\boldsymbol{X},\boldsymbol{\theta})}{P(m)}P(m|C) + \frac{1-P(m|\boldsymbol{X},\boldsymbol{\theta})}{1-P(m)}(1-P(m|C))\right) \tag{16}$$

$$= P(C)\left(\frac{P(m|\boldsymbol{X},\boldsymbol{\theta})}{P(m)}P(m|C) - \frac{P(m|\boldsymbol{X},\boldsymbol{\theta})}{1-P(m)}(1-P(m|C)) + \frac{1-P(m|C)}{1-P(m)}\right) \tag{17}$$

$$= P(C)\left(P(m|\boldsymbol{X},\boldsymbol{\theta})\left(\frac{P(m|C)}{P(m)} - \frac{1-P(m|C)}{1-P(m)}\right) + \frac{1-P(m|C)}{1-P(m)}\right) \tag{18}$$

$$= \frac{P(C)}{1-P(m)}\left(P(m|\boldsymbol{X},\boldsymbol{\theta})\left(\frac{P(m|C)}{P(m)} - 1\right) + 1 - P(m|C)\right). \tag{19}$$

In (**15**) we use (**13**). Step (**16**) uses the fact that M is a binary variable, and thus m and $\neg m$ are complementary events, from which we have $P(m)=1-P(\neg m)$, and similarly $P(m|C)=1-P(\neg m|C)$, and $P(m|\boldsymbol{X},\boldsymbol{\theta})=1-P(\neg m|\boldsymbol{X},\boldsymbol{\theta})$. The steps (**17**), (**18**), and (**19**), are just rearranging and cancelling terms.

GRADIENTS OF DISCRIMINATIVE OBJECTIVE FUNCTIONS

Next, in order to allow for gradient optimization, we give expressions for the gradients of these objective functions.

Throughout this section, we generally assume a probabilistic model with parameters $\boldsymbol{\theta}$ for a motif $\mathcal{M}$, and will occasionally denote the presence of $\mathcal{M}$ with $\mathcal{M}(\boldsymbol{\theta})$ to indicate that it depends on the probabilistic model $\boldsymbol{\theta}$. Similarly, $m_i(\boldsymbol{\theta})$ is used to stress that the number of sequences in condition i with occurrences of motif $\mathcal{M}$ depends on $\boldsymbol{\theta}$. The gradient expression presented below generally depend either directly on the log likelihood gradient, $\nabla \log P(\boldsymbol{X}|\boldsymbol{\theta})$, or indirectly via the gradient of the expected counts of sequences with motif occurrences, $\nabla m_i(\boldsymbol{\theta})$. Later in this supplement we will give expressions for both of these gradients.

Below, we first give gradient expressions for the likelihood difference before turning to the contingency table based measures. We will first consider 2×2 contingency tables, and give gradients for the difference of relative occurrence frequency, for the MCC, and for mutual information. We exclude Fisher's exact test due to the absence of simple expressions for its gradient. While expressions for the gradient of Pearson's X^2 statistic are available, they are not as simple as those presented below.

Likelihood difference

The gradient of the difference of the log likelihood is simply the difference of log likelihood gradients

$$\nabla \Delta \log \mathcal{L} = \sum_{i\in\text{signal}} \nabla \log P(\boldsymbol{X}_i|\boldsymbol{\theta}) - \sum_{i\in\text{control}} \nabla \log P(\boldsymbol{X}_i|\boldsymbol{\theta}) \qquad \textbf{(20)}$$

For the case of hidden Markov models, we give expressions for the log likelihood gradient $\nabla \log P(\boldsymbol{X}|\boldsymbol{\theta})$ in a later section.

Difference of relative frequency of occurrence

Adopting the notation of supplementary table T2 and assuming that conditions 1 and 2 represent signal and control respectively, the gradient of the difference of relative frequencies of sequences with motifs between the signal and control is given by

$$\nabla \Delta F = \frac{\nabla m_1(\boldsymbol{\theta})}{n_1} - \frac{\nabla m_2(\boldsymbol{\theta})}{n_2}. \qquad \textbf{(21)}$$

Mutual information

To derive an expression for the gradient of the mutual information of condition and motif presence we consider the gradient of (**6**),

$$\nabla I(C;\mathcal{M}(\boldsymbol{\theta})) = \frac{1}{\sum_i n_i} \left(\sum_i (\nabla m_i(\boldsymbol{\theta})) \log_2 \frac{m_i(\boldsymbol{\theta})}{n_i - m_i(\boldsymbol{\theta})} - \left(\sum_i \nabla m_i(\boldsymbol{\theta}) \right) \log_2 \frac{\sum_i m_i(\boldsymbol{\theta})}{\sum_i n_i - m_i(\boldsymbol{\theta})} \right). \qquad \textbf{(22)}$$

Matthews correlation coefficient

To find an expression for the gradient of the MCC we first adopt the notation of supplementary table T2,

$$\text{MCC} = \frac{1}{\sqrt{n_1 n_2}} \cdot \frac{n_2 m_1(\boldsymbol{\theta}) - n_1 m_2(\boldsymbol{\theta})}{\sqrt{(m_1(\boldsymbol{\theta})+m_2(\boldsymbol{\theta}))(N - m_1(\boldsymbol{\theta}) - m_2(\boldsymbol{\theta}))}}, \qquad \textbf{(23)}$$

where $N = n_1 + n_2$. We now consider first the gradient of the numerator of the second term of (**23**),

$$\nabla (n_2 m_1(\boldsymbol{\theta}) - n_1 m_2(\boldsymbol{\theta})) = n_2 \nabla m_1(\boldsymbol{\theta}) - n_1 \nabla m_2(\boldsymbol{\theta}). \qquad \textbf{(24)}$$

The gradient of the denominator of the second term of (**23**) is

$$\nabla \sqrt{(m_1(\boldsymbol{\theta})+m_2(\boldsymbol{\theta}))(N - m_1(\boldsymbol{\theta}) - m_2(\boldsymbol{\theta}))} = \frac{\left(\frac{N}{2} - m_1(\boldsymbol{\theta}) - m_2(\boldsymbol{\theta})\right) \cdot (\nabla m_1(\boldsymbol{\theta}) + \nabla m_2(\boldsymbol{\theta}))}{\sqrt{(m_1(\boldsymbol{\theta})+m_2(\boldsymbol{\theta}))(N - m_1(\boldsymbol{\theta}) - m_2(\boldsymbol{\theta}))}}. \qquad \textbf{(25)}$$

Using these two expressions and the quotient rule for differentiation, and canceling a few terms we have the following somewhat lengthy expression for the gradient of the MCC,

$$\nabla \text{MCC} = \frac{1}{\sqrt{n_1 n_2}} \cdot \frac{1}{\sqrt{(m_1(\boldsymbol{\theta})+m_2(\boldsymbol{\theta}))(N - m_1(\boldsymbol{\theta}) - m_2(\boldsymbol{\theta}))}} \cdot \left(n_2 \nabla m_1(\boldsymbol{\theta}) - n_1 \nabla m_2(\boldsymbol{\theta}) + (n_2 m_1(\boldsymbol{\theta}) - n_1 m_2(\boldsymbol{\theta})) \left(\frac{N}{2} - m_1(\boldsymbol{\theta}) - m_2(\boldsymbol{\theta}) \right) \cdot \frac{\nabla m_1(\boldsymbol{\theta}) + \nabla m_2(\boldsymbol{\theta})}{(m_1(\boldsymbol{\theta})+m_2(\boldsymbol{\theta}))(N - m_1(\boldsymbol{\theta}) - m_2(\boldsymbol{\theta}))} \right). \qquad \textbf{(26)}$$

Classification probability - MMIE

Our MMIE learning routine uses gradient optimization for the HMM parameters $\boldsymbol{\theta} = (\theta_i)_i$. The other MMIE parameters, i.e. the class prior $P(C)$ and the conditional motif occurrence priors $P(M|C)$, are simply reestimated by our MMIE learning routine. Thus, for MMIE, we only give the gradient of the classification probability $\nabla P(C|\boldsymbol{X},\boldsymbol{\theta})$ with respect to the HMM parameters $\boldsymbol{\theta}$, $\nabla = \left(\frac{\partial}{\partial \theta_i}\right)_i$,

$$\nabla P(C|\boldsymbol{X},\boldsymbol{\theta}) = \frac{P(C)}{1 - P(m)} \left(\frac{P(m|C)}{P(m)} - 1 \right) \cdot \nabla P(m|\boldsymbol{X},\boldsymbol{\theta}). \qquad \textbf{(27)}$$

As the global MMIE objective (**10**) is given by the sum of log probabilities of correct classification of the individual

sequences, we finally consider

$$\nabla \log P(C|\boldsymbol{X},\boldsymbol{\theta}) = \frac{\nabla P(C|\boldsymbol{X},\boldsymbol{\theta})}{P(C|\boldsymbol{X},\boldsymbol{\theta})} = \frac{P(C)}{1-P(m)}\left(\frac{P(m|C)}{P(m)}-1\right)\frac{\nabla P(m|\boldsymbol{X},\boldsymbol{\theta})}{P(C|\boldsymbol{X},\boldsymbol{\theta})}. \quad \mathbf{(28)}$$

MEASURES FOR CONDITIONAL ASSOCIATION

Our method makes use of conditional mutual information (cMI) (6), as mentioned in the multiple motif mode section of the methods part of the main manuscript. cMI of a variable X and a variable Y given a variable Z, $I(X;Y|Z)$, is defined as,

$$\begin{aligned} I(X;Y|Z) &= \sum_{\substack{x\in X\\ y\in Y\\ z\in Z}} P(x,y,z)\log_2\frac{P(x,y|z)}{P(x|z)P(y|z)} \\ &= \sum_{\substack{x\in X\\ y\in Y\\ z\in Z}} P(x,y,z)\log_2\frac{P(x,y,z)P(z)}{P(x,z)P(y,z)}. \end{aligned} \quad \textbf{(29)}$$

In particular, we use it to determine cMI of conditions of a contrast $\mathcal{C}$ and occurrence of motif $\mathcal{A}$ given occurrences of motif $\mathcal{B}$ (cMICO),

$$\text{cMICO}(\mathcal{C};\mathcal{A}|\mathcal{B}) = I(\mathcal{C};\mathcal{A}|\mathcal{B}), \quad \textbf{(30)}$$

as well as to define motif pair cMI of occurrences of two motifs $\mathcal{A}$ and $\mathcal{B}$ given conditions of a contrast $\mathcal{C}$,

$$\text{motif pair cMI}(\mathcal{A};\mathcal{B}|\mathcal{C}) = I(\mathcal{A};\mathcal{B}|\mathcal{C}). \quad \textbf{(31)}$$

cMICO of a contrast $\mathcal{C}$ and a motif $\mathcal{A}$ given a motif $\mathcal{B}$ measures the discriminatory contribution of $\mathcal{A}$ across the contrast $\mathcal{C}$ after accounting for the discriminatory contribution of $\mathcal{B}$. Motif pair cMI of $\mathcal{A}$ and $\mathcal{B}$ across $\mathcal{C}$ quantifies how strongly occurrences of $\mathcal{A}$ and $\mathcal{B}$ are associated throughout the contrast.

Motif pair MI and motif pair cMI

Our usage of cMICO and motif pair cMI for filtering (see page 16 of this supplement) is motivated by FIRE (12). Unlike our criterion, however, FIRE uses the (non-conditional) motif pair MI in place of the motif pair cMI. In our opinion motif pair cMI improves over motif pair MI, as illustrated by the two cases in supplementary figures S2 and S3. In the first case, two motifs that independently occur within each condition are found as associated by MI, but not by cMI. Conversely, in the second case two motifs that are dependently occurring within each condition are only found as associated according to cMI, but not according to MI.

In other words, usage of (non-conditional) motif-pair MI may lead to the conclusion that independently occurring motifs are occurring dependently, and conversely that dependent motifs are occurring independently, while cMI does not have this problem. Theses cases are of course instances of Simpson's paradox (13).

A Condition 1

	A	$\neg A$
B	1	9
$\neg B$	9	81

B Condition 2

	A	$\neg A$
B	81	9
$\neg B$	9	1

C Marginal (1+2)

	A	$\neg A$
B	82	18
$\neg B$	18	82

Supplementary figure S2. Two motifs occur independently in condition 1, and independently in condition 2, but their marginal distribution appears dependent. In this case motif pair cMI yields 0.44 bit, while motif pair MI yields 61 bit (calculations done after adding a pseudo-count of 1).

A Condition 1

	A	$\neg A$
B	40	0
$\neg B$	0	60

B Condition 2

	A	$\neg A$
B	0	60
$\neg B$	40	0

C Marginal (1+2)

	A	$\neg A$
B	40	60
$\neg B$	40	60

Supplementary figure S3. Two motifs are dependently occurring in condition 1, and dependently in condition 2, but their marginal distribution appears independent. In this case motif pair cMI yields 167 bit, while motif pair MI yields 0 bit (calculations done after adding a pseudo-count of 1).

HMMS FOR MOTIF DISCOVERY

INFERENCE WITH HMM

This section first introduces notation and then gives brief but concise definitions of important algorithms for inference using HMMs.

Relevant literature on HMM includes a famous review by (14), as well as textbooks on speech recognition applications (15), biological sequence applications (16), and more theoretical aspects (17). Also, general machine learning textbooks may serve as introduction to the theory of HMM (7, 18).

Hidden Markov modeling involves two spaces, one that represents the observable entities, and another one that represents underlying states. Both observation and state space may be continuous or discrete. Here we will only consider discrete observation and state spaces.

Formal definition of hidden Markov models

The notation and definitions here follow (19) and (14).

Hidden Markov model Let $A=(a_{ij})$ be an $N \times N$ stochastic matrix, i.e. $\sum_{j=1}^{N} a_{ij}=1$ for all $1 \leq i \leq N$. Let $a=(a_i), 1 \leq i \leq N$ be a probability distribution, i.e. $\sum_{i=1}^{N} a_i = 1$. For each $1 \leq i \leq N$ let $b_i(y)$ be a probability density: $\int b_i(y)dy=1$. Let $\boldsymbol{\theta}$ be the triple $A,a,b=\{b_i\}$. We define a stochastic process $\boldsymbol{X}=\{X_t\}_{t=1}^{T}$ with density

$$P(\boldsymbol{X}|\boldsymbol{\theta}) = P(X_1=x_1, X_2=x_2, \ldots, X_T=x_T|\boldsymbol{\theta}) = \sum_{i_0,i_1,\ldots,i_T=1}^{N} a_{i_0} a_{i_0 i_1} b_{i_1}(x_1) a_{i_1 i_2} b_{i_2}(x_2) \cdots a_{i_{T-1} i_T} b_{i_T}(x_T). \quad \textbf{(32)}$$

Then $\boldsymbol{\theta}$ is a hidden Markov model for $\{X_t\}$.

Hidden Markov model with start and end state As above, let $A=(a_{ij})$ be an $N \times N$ stochastic matrix, and for each $1 \leq i \leq N$ let $b_i(y)$ be a probability density. Let $\boldsymbol{\theta}$ be the pair $A,b=\{b_i\}$. We define a stochastic process $\boldsymbol{X}=\{X_t\}$ with density

$$P(\boldsymbol{X}|\boldsymbol{\theta}) = P(X_1=x_1, X_2=x_2, \ldots, X_T=x_T|\boldsymbol{\theta}) = \sum_{i_1,\ldots,i_T=1}^{N} a_{1 i_1} b_{i_1}(x_1) a_{i_1 i_2} b_{i_2}(x_2) \cdots a_{i_{T-1} i_T} b_{i_T}(x_T) a_{i_T 1}. \quad \textbf{(33)}$$

Then $\boldsymbol{\theta}$ is a hidden Markov model with start and end state S_1 for $\{X_t\}$. Below we, will exclusively use HMMs with start and end state S_1.

The start and end state S_1 is the only state to emit a specific symbol, ϵ, the so called empty symbol that is never occurring in the observations and which is not emitted by any other state. It is assumed that the observation sequence $\boldsymbol{X}=X_1 X_2 \ldots X_T$ is pre- and postfixed by the empty symbol ϵ, i.e. that $X_0 = X_{T+1} = \epsilon$, thus forcing any valid state path to begin and end in that state.

Inference with HMMs

HMMs are useful probabilistic models of sequence data because they can give answers to questions like which state the system was in at a given point in the observation.

Important tools to perform efficient inference with HMMs are the forward and backward algorithms that compute two matrices $\boldsymbol{\alpha}$ and $\boldsymbol{\beta}$ of likelihoods of partial observations, such that

$$\alpha_t(i) := P(X_1 X_2 \ldots X_t, q_t = S_i|\boldsymbol{\theta}) \quad \textbf{(34)}$$

is the conditional joint probability of the partial observation sequence $X_1 X_2 \ldots X_t$ and being in state S_i at time t given the model $\boldsymbol{\theta}$. The matrix $\boldsymbol{\beta}$ is defined by

$$\beta_t(i) := P(X_{t+1} X_{t+2} \ldots X_T | q_t = S_i, \boldsymbol{\theta}) \quad \textbf{(35)}$$

and gives the conditional probability of the partial observation sequence $X_{t+1} X_{t+2} \ldots X_T$ given the model $\boldsymbol{\theta}$ and given that the state at time t is S_i.

Algorithm to compute the forward matrix

The following algorithm computes the forward matrix.

1. Initialization ($t=0$):

$$\alpha_0(j) = \begin{cases} 1 & \text{for } j=1 \\ 0 & \text{for } 1<j \leq N \end{cases} \quad \textbf{(36)}$$

2. Recursion ($t=1,\ldots,T+1$):

$$\alpha_t(j) = b_j(X_t) \sum_{i=1}^{N} \alpha_{t-1}(i) a_{ij} \quad \text{for } 1 \leq j \leq N \quad \textbf{(37)}$$

Regarding the initialization step in the algorithm, note that we use an HMM with start and end state, assuming that it is initialized in state $q_0 = S_1$.

Algorithm to compute the backward matrix

The following algorithm computes the backward matrix.

1. Initialization ($t=T+1$):

$$\beta_{T+1}(j) = \begin{cases} 1 & \text{for } j=1 \\ 0 & \text{for } 1<j \leq N \end{cases} \quad \textbf{(38)}$$

2. Recursion ($t=T,\ldots,0$):

$$\beta_t(i) = \sum_{j=1}^{N} a_{ij} b_j(X_{t+1}) \beta_{t+1}(j) \quad \text{for } 1 \leq i \leq N \quad \textbf{(39)}$$

Regarding the initialization step in the algorithm, note again that we use an HMM with start and end state, assuming that it has to end in state $q_{T+1} = S_1$.

Computing the posterior probability of being in a state

Using the forward and backward matrices we find that the joint probability of being in state S_i at time t and an observation sequence $\boldsymbol{X}=X_1X_2\ldots X_T$ given the model $\boldsymbol{\theta}$ is given by

$$P(\boldsymbol{X},q_t=S_i|\boldsymbol{\theta})=\alpha_t(i)\beta_t(i). \tag{40}$$

And, using the Bayesian theorem, from this we see that the posterior probability of being in state S_i at time t given an observation sequence $\boldsymbol{X}=X_1X_2\ldots X_T$ is given by

$$P(q_t=S_i|\boldsymbol{X},\boldsymbol{\theta})=\frac{P(\boldsymbol{X},q_t=S_i|\boldsymbol{\theta})}{P(\boldsymbol{X}|\boldsymbol{\theta})}=\frac{\alpha_t(i)\beta_t(i)}{P(\boldsymbol{X}|\boldsymbol{\theta})}. \tag{41}$$

Similarly, if we want to compute the posterior probability of a transition from state S_i to state S_j at time t we may use

$$\begin{aligned}P(q_t=S_i,q_{t+1}=S_j|\boldsymbol{X},\boldsymbol{\theta})&=\frac{P(\boldsymbol{X},q_t=S_i,q_{t+1}=S_j|\boldsymbol{\theta})}{P(\boldsymbol{X}|\boldsymbol{\theta})}\\&=\frac{\alpha_t(i)a_{ij}b_j(X_{t+1})\beta_{t+1}(j)}{P(\boldsymbol{X}|\boldsymbol{\theta})}.\end{aligned} \tag{42}$$

Computing the likelihood

By marginalizing (**40**) over all states at any time t we can compute the likelihood of an observation sequence $\boldsymbol{X}=X_1X_2\ldots X_T$ given the model $\boldsymbol{\theta}$,

$$P(\boldsymbol{X}|\boldsymbol{\theta})=\sum_{i=1}^{N}P(\boldsymbol{X},q_t=S_i|\boldsymbol{\theta})=\sum_{i=1}^{N}\alpha_t(i)\beta_t(i). \tag{43}$$

For HMMs with start and end state S_1 another way of determining the likelihood from the forward matrix alone is

$$P(\boldsymbol{X}|\boldsymbol{\theta})=P(\boldsymbol{X},q_{T+1}=S_1|\boldsymbol{\theta})=\alpha_{T+1}(1), \tag{44}$$

and similarly, using only the backward matrix:

$$P(\boldsymbol{X}|\boldsymbol{\theta})=P(\boldsymbol{X},q_0=S_1|\boldsymbol{\theta})=\beta_0(1). \tag{45}$$

Scaled forward-backward matrices

With increasing length of the observation the numbers in the matrices $\boldsymbol{\alpha}$ and $\boldsymbol{\beta}$ quickly become smaller than what can be represented by floating point numbers. To alleviate this problem a scaling method can be used, which is detailed below.

The scaled forward-backward algorithm determines two matrices $\tilde{\boldsymbol{\alpha}}$ and $\tilde{\boldsymbol{\beta}}$, as well as a scaling vector $\boldsymbol{s}$ such that

$$\alpha_t(i)=\tilde{\alpha}_t(i)\prod_{k=0}^{t}s_k=\tilde{\alpha}_t(i)\prod_{k=1}^{t}s_k, \tag{46}$$

and

$$\beta_t(i)=\tilde{\beta}_t(i)\prod_{k=t}^{T+1}s_k. \tag{47}$$

In equation (**46**) the latter identity is due to $s_0=1$.

Algorithm to compute the scaled forward matrix

The following algorithm computes the scaled forward matrix $\tilde{\boldsymbol{\alpha}}$ and the scaling vector $\boldsymbol{s}$.

1. Initialization ($t=0$):

$$\tilde{\alpha}_0(j)=\begin{cases}1 & \text{for } j=1\\ 0 & \text{for } 1<j\leq N\end{cases} \tag{48}$$

$$s_0=1 \tag{49}$$

2. Recursion ($t=1,\ldots,T+1$):

$$\hat{\alpha}_t(j)=b_j(X_t)\sum_{i=1}^{N}\tilde{\alpha}_{t-1}(i)a_{ij} \qquad \text{for } 1\leq j\leq N \tag{50}$$

$$s_t=\sum_{i=1}^{N}\hat{\alpha}_t(i) \tag{51}$$

$$\tilde{\alpha}_t(j)=\frac{\hat{\alpha}_t(j)}{s_t} \qquad \text{for } 1\leq j\leq N \tag{52}$$

Note that the algorithm to compute the scaled forward matrix $\tilde{\boldsymbol{\alpha}}$ differs from the algorithm for the unscaled forward matrix $\boldsymbol{\alpha}$ in that first an intermediate value $\hat{\alpha}_t$ is computed, which is subsequently summed over to yield s_t. This sum is then used to scale the values in the matrix $\hat{\boldsymbol{\alpha}}$ for time t, yielding $\tilde{\boldsymbol{\alpha}}$.

Algorithm to compute the scaled backward matrix

The following algorithm computes the backward matrix $\tilde{\boldsymbol{\beta}}$ using the scaling vector $\boldsymbol{s}$.

1. Initialization ($t=T+1$):

$$\tilde{\beta}_{T+1}(j)=\begin{cases}\frac{1}{s_{T+1}} & \text{for } j=1\\ 0 & \text{for } 1<j\leq N\end{cases} \tag{53}$$

2. Recursion ($t=T,\ldots,0$):

$$\hat{\beta}_t(i)=\sum_{j=1}^{N}a_{ij}b_j(X_{t+1})\tilde{\beta}_{t+1}(j) \qquad \text{for } 1\leq i\leq N \tag{54}$$

$$\tilde{\beta}_t(i)=\frac{\hat{\beta}_t(i)}{s_t} \tag{55}$$

Note that in the algorithm for the scaled backward matrix the same s_t values are used that were determined in the calculation of the scaled forward matrix.

Computing the likelihood

Building on (**44**), and using the scaling coefficient vector $\boldsymbol{s}$, the likelihood of the observation sequence $\boldsymbol{X}=X_1X_2\ldots X_T$ given the model $\boldsymbol{\theta}$ may be computed by

$$P(\boldsymbol{X}|\boldsymbol{\theta})=\alpha_{T+1}(1)=\tilde{\alpha}_{T+1}(1)\prod_{t=0}^{T+1}s_t=\prod_{t=1}^{T+1}s_t, \quad (\mathbf{56})$$

as $\tilde{\alpha}_{T+1}(1)=1$, and $s_0=1$. Similarly, and numerically preferably, the log-likelihood may be determined by

$$\log P(\boldsymbol{X}|\boldsymbol{\theta})=\sum_{t=1}^{T+1}\log s_t. \quad (\mathbf{57})$$

Computing the posterior probability of being in a state

An expression based on the scaled variants of the forward and backward matrices, $\tilde{\boldsymbol{\alpha}}$ and $\tilde{\boldsymbol{\beta}}$ corresponding to (**41**) for the posterior probability of being in a state S_i at time t given the observation sequence $\boldsymbol{X}=X_1X_2\ldots X_T$ and the model $\boldsymbol{\theta}$ is

$$\begin{aligned}P(q_t=S_i|\boldsymbol{X},\boldsymbol{\theta})&=\frac{\alpha_t(i)\beta_t(i)}{P(\boldsymbol{X}|\boldsymbol{\theta})}\\&=\frac{\tilde{\alpha}_t(i)\prod_{k=0}^{t}s_k\tilde{\beta}_t(i)\prod_{k=t}^{T+1}s_k}{\prod_{t=0}^{T+1}s_t}\\&=\tilde{\alpha}_t(i)\tilde{\beta}_t(i)s_t.\end{aligned} \quad (\mathbf{58})$$

Similarly, (**42**) may be computed as

$$\begin{aligned}P(q_t=S_i,q_{t+1}=S_j|\boldsymbol{X},\boldsymbol{\theta})&=\frac{\alpha_t(i)a_{ij}b_j(X_{t+1})\beta_{t+1}(j)}{P(\boldsymbol{X}|\boldsymbol{\theta})}\\=\frac{\tilde{\alpha}_t(i)\prod_{k=0}^{t}s_k a_{ij}b_j(X_{t+1})\tilde{\beta}_t(i)\prod_{k=t+1}^{T+1}s_k}{\prod_{t=0}^{T+1}s_t}&\\&=\tilde{\alpha}_t(i)a_{ij}b_j(X_{t+1})\tilde{\beta}_{t+1}(j).\end{aligned} \quad (\mathbf{59})$$

Expected number of transitions

By summing over (**42**) or over (**59**), we compute for each observation sequence $\boldsymbol{X}^m$ the expected number of transitions $\mathcal{A}_{ij}^m$ from state S_i to state S_j,

$$\begin{aligned}\mathcal{A}_{ij}^m&=\sum_{t=0}^{T}P(q_t=S_i,q_{t+1}=S_j|\boldsymbol{X}^m,\boldsymbol{\theta})\\&=\sum_{t=0}^{T}\frac{\alpha_t^m(i)a_{ij}b_j(X_{t+1}^m)\beta_{t+1}^m(j)}{P(\boldsymbol{X}^m|\boldsymbol{\theta})}\\&=\sum_{t=0}^{T}\tilde{\alpha}_t^m(i)a_{ij}b_j(X_{t+1}^m)\tilde{\beta}_{t+1}^m(j),\end{aligned} \quad (\mathbf{60})$$

By subsequently summing over all M observation sequences, we determine the total expected number of transitions $\mathcal{A}_{ij}$ from state S_i to state S_j,

$$\mathcal{A}_{ij}=\sum_{m=1}^{M}\mathcal{A}_{ij}^m. \quad (\mathbf{61})$$

Expected number of observations

The expected number of times that observation x occurs in state S_i in observation sequence $\boldsymbol{X}^m$ is given by summing (**41**) or (**58**) over those times t at which x is observed,

$$\begin{aligned}\mathcal{B}_i^m(x)&=\sum_{\{t|X_t^m=x\}}P(q_t=S_i|\boldsymbol{X}^m,\boldsymbol{\theta})\\&=\sum_{\{t|X_t^m=x\}}\frac{\alpha_t^m(i)\beta_t^m(i)}{P(\boldsymbol{X}^m|\boldsymbol{\theta})}\\&=\sum_{\{t|X_t^m=x\}}\tilde{\alpha}_t^m(i)\tilde{\beta}_t^m(i)s_t.\end{aligned} \quad (\mathbf{62})$$

Again, by summing over all M observation sequences, the total expected number of emissions of kind x in state S_i are determined,

$$\mathcal{B}_i(x)=\sum_{m=1}^{M}\mathcal{B}_i^m(x). \quad (\mathbf{63})$$

HIGHER-ORDER HMMS

As mentioned in the introduction of the main manuscript, HMMs can account for interacting neighboring positions. There are several different ways in which this can be achieved via higher-order dependencies (illustrated in supplementary figure S4). In the simplest case, both emission and state transition probabilities depend simply on the current state (supplementary figure S4A). However, state transition probabilities can be modeled to additionally depend on more than just the immediately preceding state (supplementary figures S4B and C). Similarly, also emission probabilities can depend both on the current state as well as on preceding emissions (supplementary figures S4D and E). Alternatively, aside from the current state, emission probabilities can also depend on preceding states (supplementary figures S4F and G) Finally, also combinations of the afore-mentioned cases are possible (not shown).

Note that in this manuscript we only consider HMMs with first-order transition and first-order emission probabilities (supplementary figure S4A). For higher-order models, the equations presented in this section become slightly more complicated, but retain the essential property that inference and learning can be done in time linear in the length of the data.

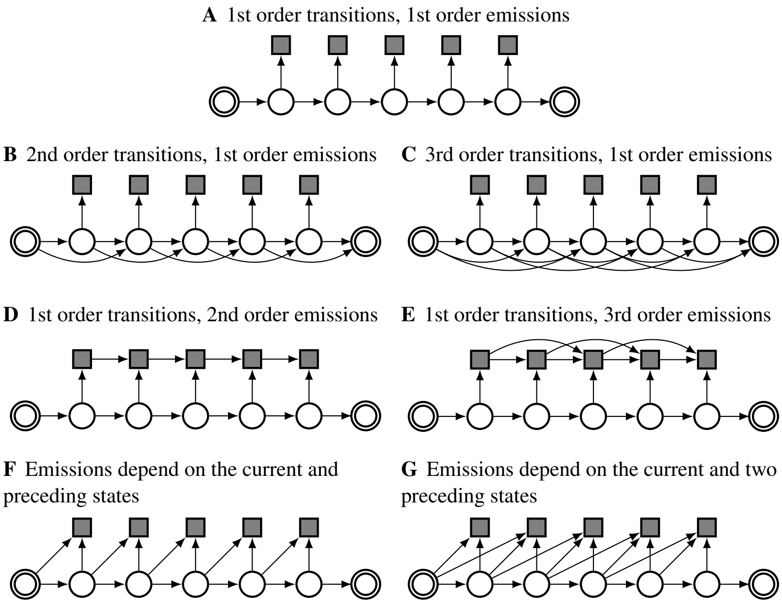

Supplementary figure S4. Graphical model notation for HMMs of different transition and emission orders. Circles correspond to state variables, rectangles to emission variables. Doubly-circled states are non-emitting start and stop states. Filled nodes are observed. **(A)** Standard HMM for a sequence of length 5. **(B)** Transition probabilities depend on the preceding two states. **(C)** Transition probabilities depend on the preceding three states. **(D)** Emission probabilities depend on the current state and the preceding emission. **(E)** Emission probabilities depend on the current state and the two preceding emissions. **(F)** Emission probabilities depend on the current and the preceding state. **(G)** Emission probabilities depend on the current state and the two preceding states.

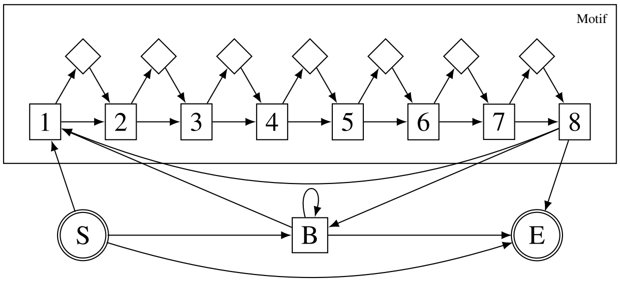

Supplementary figure S5. State transition graph of a binding site HMM with a motif of 8 nucleotides length and a background state B. The model includes a start state S, and an end state E. The numbered states represent motif chain states, the diamond shaped states are (optional) insert states. The box around the motif is an instance of plate notation from probabilistic graphical models (see (11)) and indicates that there may be multiple motifs, which may have different lengths. Note that the implementation in Discrover uses a combined start/end state, rather than two separate states as depicted.

BINDING SITE HMM

Supplementary figure S5 illustrates the default topology of binding site HMMs as used by Discrover.

We now describe how to calculate the posterior probability of at least one motif occurrence, and then give expressions for the gradient thereof.

Given an HMM $\boldsymbol{\theta}$ that models binding sites for motif $\mathcal{M}$, let M be the set of states of $\mathcal{M}$ in $\boldsymbol{\theta}$. The likelihood of no occurrence of $\mathcal{M}$ is the joint probability given $\boldsymbol{\theta}$ of data and all state paths that avoid transitions through M. This is the sum of probabilities of all state paths $\boldsymbol{q}$ that do not transit through M,

$$P(\neg\mathcal{M},\boldsymbol{X}|\boldsymbol{\theta}) = \sum_{\boldsymbol{q}:q_i\notin M} P(\boldsymbol{q},\boldsymbol{X}|\boldsymbol{\theta}). \qquad (\mathbf{64})$$

With this we can compute the posterior probability of no occurrence of $\mathcal{M}$,

$$P(\neg\mathcal{M}|\boldsymbol{X},\boldsymbol{\theta}) = \frac{P(\neg\mathcal{M},\boldsymbol{X}|\boldsymbol{\theta})}{P(\boldsymbol{X}|\boldsymbol{\theta})}. \qquad (\mathbf{65})$$

By considering the complementary element, we have the posterior probability of at least one occurrence of $\mathcal{M}$,

$$P(\mathcal{M}|\boldsymbol{X},\boldsymbol{\theta}) = 1 - P(\neg\mathcal{M}|\boldsymbol{X},\boldsymbol{\theta}). \qquad (\mathbf{66})$$

However, it would be fairly inefficient to enumerate all paths $\boldsymbol{q}: q_i \notin M$ in order to compute $P(\neg\mathcal{M},\boldsymbol{X}|\boldsymbol{\theta})$ according to (**64**). Instead, we may consider modified parameters $\boldsymbol{\theta}'$ which are identical to $\boldsymbol{\theta}$ except for having the transition probabilities to all states $q\in M$ set to zero. It is important not to renormalise after setting these zero. Specifically, when $\boldsymbol{\theta} = (\theta_i)_{i=1,\ldots,n}$ and $\boldsymbol{\theta}' = \left(\theta'_i\right)_{i=1,\ldots,n}$ then $\theta'_i = 0$ for all i for which θ_i represents a transition probability to a state $q \in M$, and $\theta'_i = \theta_i$ otherwise. We then have

$$P(\neg\mathcal{M},\boldsymbol{X}|\boldsymbol{\theta}) = \tilde{P}\left(\boldsymbol{X}|\boldsymbol{\theta}'\right), \qquad (\mathbf{67})$$

where we use $\tilde{P}$ in $\tilde{P}(\boldsymbol{X}|\boldsymbol{\theta}')$ to indicate that this not a normalized probability, i.e. $\sum_{\boldsymbol{X}} \tilde{P}(\boldsymbol{X}|\boldsymbol{\theta}') \le 1$ in general.

However, $\tilde{P}(\boldsymbol{X}|\boldsymbol{\theta}')$ may still be computed with the forward-backward algorithm applied to $\boldsymbol{X}$ and $\boldsymbol{\theta}'$.

The gradient of the posterior occurrence probability is

$$\begin{aligned}\nabla P(\mathcal{M}|\boldsymbol{X},\boldsymbol{\theta}) &= \frac{\tilde{P}(\boldsymbol{X}|\boldsymbol{\theta}')\nabla P(\boldsymbol{X}|\boldsymbol{\theta}) - P(\boldsymbol{X}|\boldsymbol{\theta})\nabla \tilde{P}(\boldsymbol{X}|\boldsymbol{\theta}')}{P(\boldsymbol{X}|\boldsymbol{\theta})^2} \\ &= \frac{\tilde{P}(\boldsymbol{X}|\boldsymbol{\theta}')}{P(\boldsymbol{X}|\boldsymbol{\theta})}\left(\frac{\nabla P(\boldsymbol{X}|\boldsymbol{\theta})}{P(\boldsymbol{X}|\boldsymbol{\theta})} - \frac{\nabla \tilde{P}(\boldsymbol{X}|\boldsymbol{\theta}')}{\tilde{P}(\boldsymbol{X}|\boldsymbol{\theta}')}\right) \\ &= P(\neg\mathcal{M}|\boldsymbol{X},\boldsymbol{\theta})\left(\nabla \log P(\boldsymbol{X}|\boldsymbol{\theta}) - \nabla \log \tilde{P}(\boldsymbol{X}|\boldsymbol{\theta}')\right).\end{aligned} \tag{68}$$

This expression is in terms of the gradient of the log likelihood which is given in the next section.

HMM GRADIENT CALCULUS

Gradient learning is an iterative, local learning method. The parameter estimates $\boldsymbol{\theta}_k$ are improved in iteration k by computing the direction of steepest increase of the likelihood $\nabla P(\boldsymbol{X}|\boldsymbol{\theta})$ and taking small steps into that direction.

$$\boldsymbol{\theta}_{k+1} = \boldsymbol{\theta}_k + \eta_k \nabla P(\boldsymbol{X}|\boldsymbol{\theta}) \tag{69}$$

The step size η_k may be a sufficiently small constant ($\eta_k = \eta$), decrease with the iterations (e.g. $\eta_k = \frac{1}{k}$), or be chosen (approximately) optimal for each iteration k using line searching. Our method makes use of the Moré-Thuente line searching algorithm (20).

Likelihood gradient

We first consider expressions for the likelihood gradient of a single sequence $\boldsymbol{X}^m$.

Partial derivatives w.r.t. transition probabilities Expression (**70**) for the partial derivatives of the likelihood with respect to the transition probabilities was given by Leonard Baum (21),

$$\frac{\partial P(\boldsymbol{X}^m|\boldsymbol{\theta})}{\partial a_{ij}} = \sum_{t=0}^{T} \alpha_t^m(i) b_j(X_{t+1}^m) \beta_{t+1}^m(j) \tag{70}$$

$$= \frac{\mathcal{A}_{ij}^m}{a_{ij}} P(\boldsymbol{X}^m|\boldsymbol{\theta}). \tag{71}$$

Expression (**71**) uses the definition of the expected number of transitions from state S_i to S_j, $\mathcal{A}_{ij}^m$, as given in (**60**). From this we have the partial derivative of the log-likelihood with respect to the transition probabilities,

$$\frac{\partial \log P(\boldsymbol{X}^m|\boldsymbol{\theta})}{\partial a_{ij}} = \frac{1}{P(\boldsymbol{X}^m|\boldsymbol{\theta})} \frac{\partial P(\boldsymbol{X}^m|\boldsymbol{\theta})}{\partial a_{ij}} = \frac{\mathcal{A}_{ij}^m}{a_{ij}} \tag{72}$$

Partial derivatives w.r.t. emission probabilities An expression for the partial derivative of the likelihood with respect to the emission probabilities corresponding to the one of Baum is

$$\frac{\partial P(\boldsymbol{X}^m|\boldsymbol{\theta})}{\partial b_j(k)} = \sum_{\{t|X_t^m = k\}} \sum_{i=1}^{N} \alpha_{t-1}^m(i) a_{ij} \beta_t^m(j) \tag{73}$$

$$= \frac{1}{b_j(k)} \sum_{\{t|X_t^m = k\}} \alpha_t^m(j) \beta_t^m(j) \tag{74}$$

$$= \frac{\mathcal{B}_j^m(k)}{b_j(k)} P(\boldsymbol{X}^m|\boldsymbol{\theta}). \tag{75}$$

Expression (**74**) uses the definition of $\alpha_t^m(j)$, in (**37**), expression (**75**) uses the definition of the expected number of emissions of kind k in state S_j, $\mathcal{B}_j^m(k)$, in (**62**). From this we get the partial derivative of the log-likelihood with respect to the emission probabilities,

$$\frac{\partial \log P(\boldsymbol{X}^m|\boldsymbol{\theta})}{\partial b_j(k)} = \frac{\mathcal{B}_j^m(k)}{b_j(k)}. \tag{76}$$

Another derivation of (**72**) and (**76**) can be found in (22).

Transformed probabilities

In order to avoid boundary issues during gradient optimization Mao and Hu (23) suggest to consider quantities g_{ij} and h_{il}, which are defined to transform into the corresponding transition and emission probabilities as

$$a_{ij} = \frac{\exp(g_{ij})}{\sum_{k=1}^{N} \exp(g_{ik})} \tag{77}$$

and

$$b_i(l) = \frac{\exp(h_{il})}{\sum_{k=1}^{M} \exp(h_{ik})}. \tag{78}$$

Partial derivatives w.r.t. transformed transition probabilities Using that $\frac{\partial a_{ij}}{\partial g_{kl}} = \delta_{ik}(\delta_{jl} - a_{ij}) a_{il}$ Mao and Hu (23) give (**79**) for the partial derivative of the likelihood with respect to the transformed transition probabilities g_{ij},

$$\frac{\partial P(\boldsymbol{X}^m|\boldsymbol{\theta})}{\partial g_{ij}} = \sum_{k=1}^{N} \sum_{t=0}^{T} \alpha_t^m(i) b_k(X_{t+1}^m) \beta_{t+1}^m(k) (\delta_{kj} - a_{ij}) a_{ik} \tag{79}$$

$$= \sum_{k=1}^{N} (\delta_{kj} - a_{ij}) \sum_{t=0}^{T} \alpha_t^m(i) a_{ik} b_k(X_{t+1}^m) \beta_{t+1}^m(k) \tag{80}$$

$$= P(\boldsymbol{X}^m|\boldsymbol{\theta}) \sum_{k=1}^{N} \mathcal{A}_{ik}^m (\delta_{kj} - a_{ij}). \tag{81}$$

While (**80**) is just a reordering of (**79**), (**81**) uses the definition of $\mathcal{A}^m_{ik}$ in (**60**). From this we have the partial derivative of the log-likelihood with respect to the transformed transition probabilities

$$\frac{\partial \log P(\boldsymbol{X}^m|\boldsymbol{\theta})}{\partial g_{ij}} = \sum_{k=1}^{N} \mathcal{A}^m_{ik} \left(\delta_{kj} - a_{ij}\right). \quad \textbf{(82)}$$

Partial derivatives w.r.t. transformed emission probabilities Similarly, with $\frac{\partial b_i(l)}{\partial h_{jk}} = \delta_{ij}(\delta_{lk} - b_i(l))b_i(k)$ Mao and Hu (23) give an expression for the partial derivative of the likelihood with respect to the transformed emission probabilities h_{jk} that we can further simplify with an expression from (**62**) for the expected number of observations,

$$\frac{\partial P(\boldsymbol{X}^m|\boldsymbol{\theta})}{\partial h_{jk}} = \sum_{l=1}^{|\mathcal{A}|} \left(\sum_{\{t|X^m_t = l\}} \sum_{i=1}^{N} \alpha^m_{t-1}(i) a_{ij} \beta^m_t(j) \right) \cdot \left(\delta_{kl} - b_j(k)\right) b_j(l) \quad \textbf{(83)}$$

$$= \sum_{l=1}^{|\mathcal{A}|} \frac{1}{b_j(l)} \left(\sum_{\{t|X^m_t = l\}} \alpha^m_t(j) \beta^m_t(j) \right) \left(\delta_{kl} - b_j(k)\right) b_j(l)$$

$$= P(\boldsymbol{X}^m|\boldsymbol{\theta}) \sum_{l=1}^{|\mathcal{A}|} \mathcal{B}^m_j(l) \left(\delta_{kl} - b_j(k)\right), \quad \textbf{(84)}$$

where $|\mathcal{A}|$ denotes the size of the alphabet. From this we have the partial derivative of the log-likelihood with respect to the transformed emission probabilities

$$\frac{\partial \log P(\boldsymbol{X}^m|\boldsymbol{\theta})}{\partial h_{jk}} = \sum_{l=1}^{|\mathcal{A}|} \mathcal{B}^m_j(l) \left(\delta_{kl} - b_j(k)\right), \quad \textbf{(85)}$$

Note on gradients for non-normalized models

A comment is due regarding the calculation of the partial derivative of the (ordinary or log) likelihood with respect to the transformed transition probabilities for the non-normalized models, $\frac{\partial \tilde{P}(\boldsymbol{X}^m|\boldsymbol{\theta}')}{\partial g_{ij}}$.

Let us consider a state i that has a transition to a state $j \in M$, where M is the set of states corresponding to a motif $\mathcal{M}$. In the full model parameters $\boldsymbol{\theta}$ we have $a_{ij} > 0$ with $\sum_k a_{ik} = 1$, and in the reduced, non-normalized model parameters $\boldsymbol{\theta}'$ $a'_{ij} = 0$ with $\sum_k a'_{ik} < 1$ (see section on binding site HMM, page 11). Correspondingly, for the transformed transition probabilities and $j \in M$ we have $g_{ij} > -\infty$ and $g'_{ij} = -\infty$ for the full and non-normalized parameters $\boldsymbol{\theta}$ and $\boldsymbol{\theta}'$, respectively, while for $k \notin M$ we have $g'_{ik} = g_{ik}$.

In this case it is important to define the term in the denominator of (**77**) based on the transition probabilities of full model parameters,

$$a'_{ij} = \frac{\exp\left(g'_{ij}\right)}{\sum_{k=1}^{N} \exp(g_{ik})}. \quad \textbf{(86)}$$

Note here that only the terms in the denominator lack apostrophes to indicate that only they are referring to the full model parameters. Otherwise, the resulting gradient would erroneously point out of the probability simplex.

As described in the main manuscript, our method by default uses hybrid learning. In this mode, only the emission probabilities of the motif states are optimized by maximizing discriminative objectives through gradient learning. The transition probabilities (and the non-motif state emission probabilities) on the other hand are optimized by the Baum-Welch algorithm. Thus, in the hybrid learning mode, the note above is actually not of relevance. Yet, we feel it necessary to include it for the sake of correctness for the general case.

RUNTIME OF HMM ALGORITHMS

Supplementary table T3 gives an overview of the runtime complexities of the calculations necessary to compute the gradient. As is visible, the total runtime to compute the gradient due to Baum is $O(TE+NM)$. The formulation due to Krogh are simpler than those of Baum but depend on calculation of the expected statistics, and thus have the same cumulative runtime of $O(TE+NM)$. When transformed probabilities are used, the formulation due to Mao and Hu is $O(TEN+TN^2M)$. Our formulation reduces this to $O(TE+EN+NM^2)$.

Supplementary table T3. Runtime complexity and inter-dependence of inference algorithms. T is the length of the observation sequence, N the number of states, E the number of edges of the HMM (the number of non-zero transition probabilities). M is the size of the alphabet.

#	Algorithm	Complexity	Depends on #	Equations
1	Forward, unscaled / scaled	$O(TE)$		(**36**), (**37**) / (**48**), (**50**)
2	Backward, unscaled / scaled	$O(TE)$		(**38**), (**39**) / (**53**), (**54**)
3	Expected transitions	$O(TE)$	1, 2	(**60**)
4	Expected emissions	$O(TN)$	1, 2	(**62**)
5	$\mathcal{L}$ gradient due to Baum (21)	$O(TE+NM)$	1, 2	(**70**), (**73**)
6	$\mathcal{L}$ gradient due to Krogh (22)	$O(E+NM)$	3, 4	(**72**), (**76**)
7	$\mathcal{L}$ gradient, transformed statistics due to Mao and Hu (23)	$O(TEN+TN^2M)$	1, 2	(**79**), (**83**)
8	$\mathcal{L}$ gradient, transformed statistics	$O(EN+NM^2)$	3, 4	(**82**), (**85**)

MULTIPLE TESTING CORRECTION FOR MOTIF FINDING PROBLEMS

Any motif finding method aims to find optimal sets of parameters according to some objective function. During this (exact, approximate or heuristic) optimization many parameter values are tested and the best one is reported. Clearly, the larger the allowed motif space the higher the maximally achievable objective function is. It is thus desirable to account for the difference in number of parameters when comparing the values of the objective function on the same data for two parameter sets with differing numbers of parameters. There is no generally applicable way to account for difference in number of parameters for arbitrary objective functions. However, whenever the objective function represents a p-value P, we may, in a Bonferroni style, multiply the p-value with the size N of the motif space, to yield a corrected p-value $P_{\text{corrected}}$. In log-space we have then

$$\log P_{\text{corrected}} = \min(0, \log P + \log N). \qquad \textbf{(87)}$$

Continuous motif space sizes

Next, we outline how we determine the motif space size for continuous matrix based motif representations.

Here we propose to base the effective number of parameter in a continuous matrix on rank statistics. Considering individual positions, we assume that one to four nucleotide may be allowed. If one nucleotide is allowed, there are four possibilities of choosing this one nucleotide. When two nucleotides are allowed, e.g. nucleotides x and y then we may have $x<y$, $x=y$, $x>y$, indicating that nucleotide x is respectively less frequent, as frequent, or more frequent than nucleotide y. Thus, for two nucleotides, one can choose two of the four nucleotides and can have the two nucleotides in three relations, resulting in $\binom{4}{2}\cdot 3 = 18$ possibilities. Following this logic, we have the following formula for the total number of rankings of up to 4 elements selected from the nucleic acid alphabet,

$$J = \sum_{i=1}^{4} \binom{4}{i} \cdot K(i) = 4\cdot 1 + 6\cdot 3 + 4\cdot 13 + 1\cdot 75 = 149, \qquad \textbf{(88)}$$

where $K(i)$ is the number of total preorders of i elements (24). Then, by multiplying this number across the n positions of a motif, we have a motif space size of $N = J^n$.

DISCOVERING MULTIPLE MOTIFS

Supplementary figure S6 illustrates the first part of the multiple motif discovery mode of Discrover. First, seeds are discovered using Plasma. For each seed an HMM is initialized and independently optimized by Discrover. The HMM achieving the best MICO based p-value is accepted. In a second part further motifs are then added to this HMM as described below and illustrated in supplementary figure S7.

In turn, the single HMM motifs are added to the accepted HMM, forming candidate HMMs. The candidate HMMs are then filtered, ensuring that newly added motifs provide sufficient additional discrimination and are not redundant with previously accepted motifs. This is done by comparing in each candidate HMM the new motif first pairwise against each previously accepted motif, and then jointly against all previously accepted motifs. Candidate HMMs and corresponding single-motif HMMs are discarded when the filtering criteria—outlined below—are not met for the newly added motif, whether in any of the pairwise comparisons or in the joint comparison.

Filtering is based on conditional mutual information (cMI), calculated in two ways: (I) cMI of conditions of the contrast and occurrences of the newly added motif given occurrences of previously accepted motifs (cMICO), and (II) cMI between occurrences of newly added and previously accepted motifs given the conditions of the contrast (motif pair cMI). cMICO quantifies the discriminatory contribution of the new motif after accounting for previous ones, while motif pair cMI quantifies association between occurrences of the newly added and previously accepted motifs. See page 7 for definitions of cMICO and motif pair cMI. In order to concentrate on motifs with a large residual explanatory contribution relative to their association with previous motifs, HMMs are discarded if at least one of two criteria is fulfilled: (a) the ratio of cMICO over motif pair cMI does not meet a threshold[1], or (b) the cMICO based p-value is not significant.

As mentioned above, these criteria are first checked pairwise for the newly added motif and each previously accepted motif, and subsequently for the new motif and jointly all previously accepted motifs. In the joint comparison, an occurrence for the previously accepted motif is counted whenever any of the previously accepted motifs occurs.

Among the candidate HMMs that pass the filtering steps, we select the one whose newly added motif achieves the best cMICO based p-value. This HMM is then re-trained to optimize MICO for the feature of sequences having at least one occurrence of any of its motifs. If, after retraining, the MICO based p-value improves over the previously accepted one's, it is accepted, and further motifs may be added. Otherwise, or if all candidate motifs have been discarded, the last accepted HMM is reported.

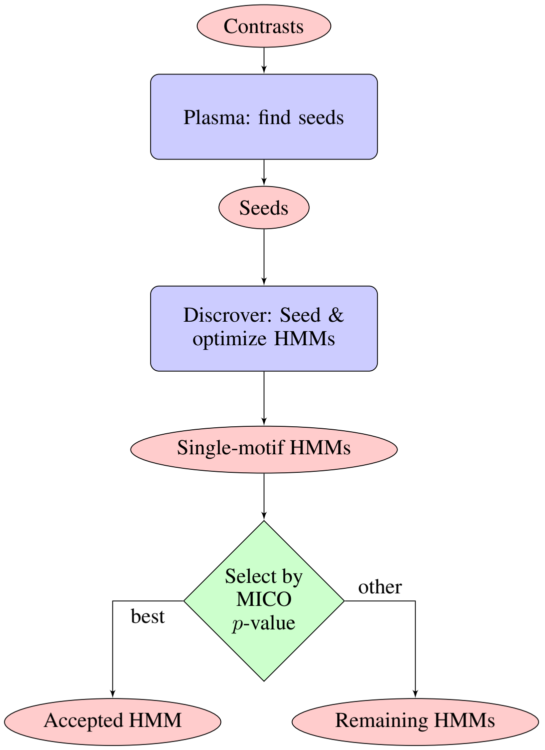

Supplementary figure S6. Flow chart of the first part of multiple motif discovery. The resulting accepted HMM and the set of remaining single-motif HMMs are the inputs for the second part, depicted in supplementary figure S7. See text for description.

Note on filtering

Note that usage of the ratio of cMICO over motif pair cMI is quite related to the usage of cMICO over motif pair MI as is done by FIRE (12). However, as already mentioned on page 7 in this supplement, unlike FIRE we use the motif pair cMI instead of motif pair MI. The intention behind this choice is to avoid pitfalls as illustrated by the two cases in supplementary figures S2 and S3. While the illustrated cases may be said to be extreme, we found the underlying issue to be real. As motif pair cMI is used in the denominator of the ratio, the filtering step is rather sensitive to misjudgement of motif pair association. It is, in our view, therefore crucial to avoid quantitatively misjudging motif pair association as may frequently happen when using motif pair MI.

[1] We use the same threshold value of 5.0 as FIRE (12). As noted by Elemento et al., the user may want to experiment with this value, as it serves as a redundancy trade-off parameter. High values yield fewer motifs, low values yield more redundant motifs.

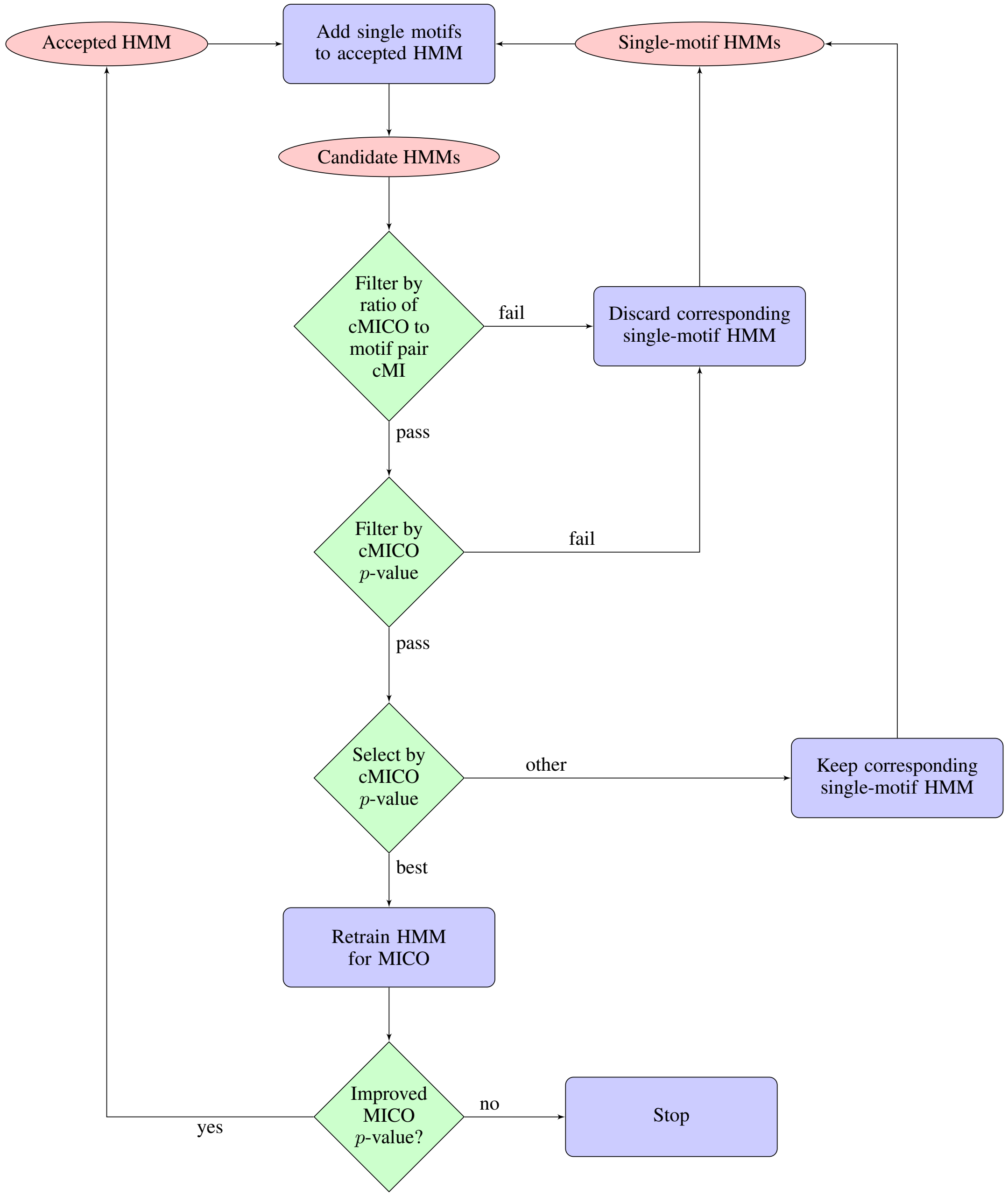

Supplementary figure S7. Flow chart of the second part of multiple motif discovery. Inputs are the accepted HMM, and the set of remaining single-motif HMMs resulting from the first part, depicted in supplementary figure S6. This part is executed until all single-motif HMMs have been accepted or discarded, or until the MICO p-value is not improved after retraining. See text for description.

GALAXY MODULE OF DISCROVER

A Simple interface **B** Advanced interface

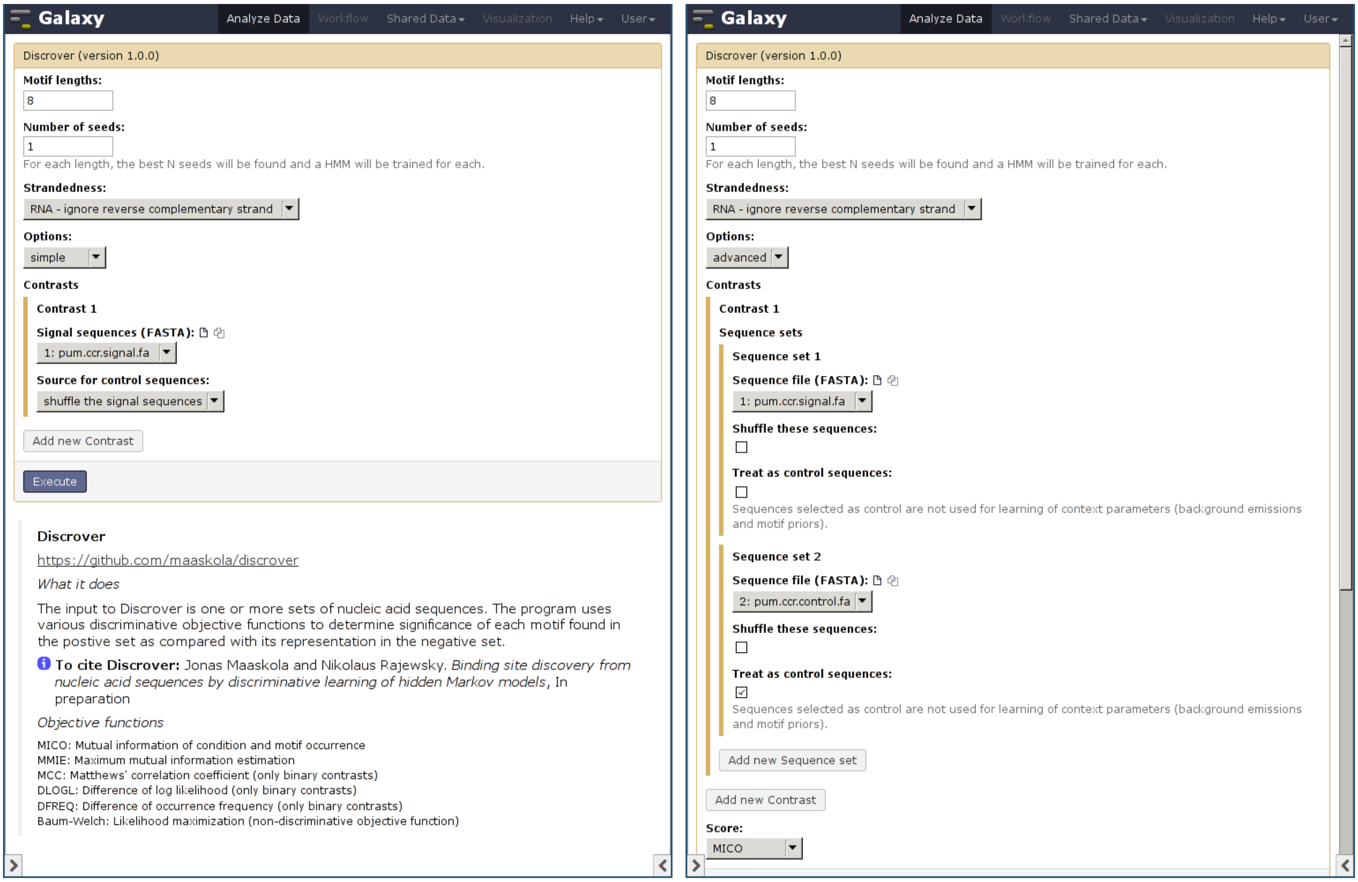

Supplementary figure S8. Screen shots of the **(A)** simple and **(B)** advanced Galaxy (25) web interfaces of Discrover. Both interfaces can be used to perform either RBP motif discovery on the forward strand only, or DBP motif discovery by also considering occurrences on the reverse complementary strand. Motif lengths may be specified as a comma-separated list of lengths or length ranges, e.g. '8', '5-10', or '5,8-10'. Multiple best seeds per length may be used. Using both interfaces, multiple contrasts may be specified for joint analysis. The simple interface is designed for easy specification of binary contrasts, and assumes the first sequence set of each contrast to be the signal sequences, while the second are assumed to be control sequences (control sequences can either be shuffles of the signal sequences, as shown in the screen shot, or they can be another set of sequences uploaded by the user). The advanced interface allows specification of contrasts of more than two conditions, and grants additional control over how the individual conditions of each contrast are to be used.

PERFORMANCE METRICS

Supervised performance metric require the knowledge of the true model used to generate data. As such they might require an exhaustive specification of all true binding sites in a synthetic sequence dataset. The first two supervised performance metrics discussed below, both defined by (26), fall into this category.

Nucleotide level performance metrics

Given a set of implanted and predicted motif coordinates, individual nucleotide positions may be classified as true and false positives, and true and false negatives, see supplementary figure S9A.

Basic nucleotide level statistics The following definitions for basic nucleotide level statistics can be found in (26).

nTP Number of nucleotides part of a site correctly predicted

nTN Number of background nucleotides correctly predicted

nFP Number of background nucleotides predicted to be part of a motif

nFN Number of nucleotides part of a site predicted as background

Nucleotide correlation coefficient Originally defined by (27), the nucleotide correlation coefficient (nCC) is the MCC applied on the nucleotide-level statistics. The nCC is given by

$$\mathrm{nCC} = \frac{nTP \cdot nTN - nFP \cdot nFN}{\sqrt{(nTP+nFN)(nTN+nFP)(nTP+nFP)(nTN+nFN)}}. \qquad \mathbf{(89)}$$

The nCC, like the general MCC, is a value between -1 and $+1$. A coefficient of $+1$ implies perfect prediction, and -1 perfect inverse prediction. A coefficient of 0 implies that the prediction performance is equivalent to that of a random prediction.

For the limit of any of the product terms under the square root in the denominator approaching zero, the limiting value of the MCC is zero.

Site level performance metrics

Basic site level statistics (28) give the following definitions for basic binding site level statistics, see supplementary figure S9B.

sTP Number of real sites that share over 50% of their nucleotides with a predicted site

sFP Number of predicted sites that share less than 50% of their nucleotides with a real site

sFN Number of real sites that share less than 50% of their nucleotides with a predicted site

These definitions are more strict than the site level statistics given by (26), which consider a single overlapping base as sufficient. Using these basic statistics, one may define the following site-level performance metrics. First, the site sensitivity sSn is defined as

$$\mathrm{sSn} = \frac{sTP}{sTP+sFN}. \qquad \mathbf{(90)}$$

Next, the site positive predictive value is defined as

$$\mathrm{sPPV} = \frac{sTP}{sTP+sFP}. \qquad \mathbf{(91)}$$

Sensitivity is also known as recall, while another name for positive predictive value is precision.

Average site performance The average site performance (sAP) is the arithmetic mean of site sensitivity sSn and of site positive predictive value sPPV,

$$\mathrm{sAP} = \frac{\mathrm{sSn}+\mathrm{sPPV}}{2}. \qquad \mathbf{(92)}$$

As both sSn and sPPV represent relative frequencies, they take on values between 0 and 1, where in both cases 0 signifies the worst performance. For the sSn a value of 1 denotes that each true site shares at least 50% of its nucleotides with a predicted site, which is equivalent to the statement that there are no false negative sites. In case of the sPPV a value of 1 denotes that none of the predicted sites is a false positive. Clearly, as the sAP is the average of these two measures, its values are confined to the same range.

Site $\mathbf{sF_1}$ score Another choice of site level statistic is the site level $\mathrm{sF_1}$ score. It is related to the average site performance, as it is the harmonic mean of site sensitivity and site positive predictive value:

$$\mathrm{sF_1} = 2\frac{\mathrm{sSn}\cdot\mathrm{sPPV}}{\mathrm{sSn}+\mathrm{sPPV}}. \qquad \mathbf{(93)}$$

Like the sASP the $\mathrm{sF_1}$ score is symmetrical in the underlying statistics, takes values between 0 and 1, and assumes its maximal value exactly when both underlying statistics achieve their maxima. However, unlike the sASP it is zero whenever either underlying statistic is zero. Whenever sSn is not equal to sPPV, the $\mathrm{sF_1}$ score is less than the sASP, otherwise they are equal.

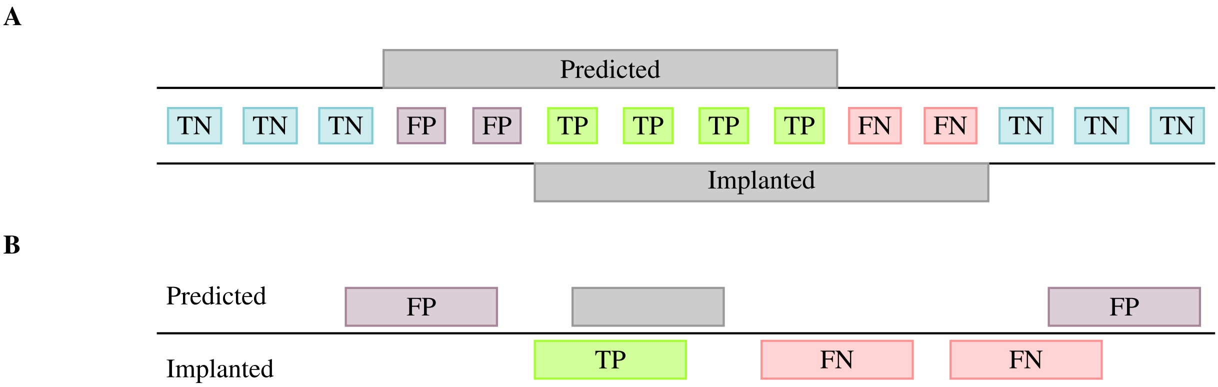

Supplementary figure S9. **(A)** Classification of nucleotide positions as true positives (TP), false positives (FP), false negatives (FN), and true negatives (TN), based on the overlap with implanted and predicted motifs. **(B)** Classification of implanted binding sites as true positives (TP) and false negatives (FN), based on ≥50% overlap with predicted sites. Classification of predicted sites as false positives (FP) based on lack of ≥50% overlap with implanted sites.

Summarization

Another topic worth discussion in the context of performance metrics is that of summarization. It is in principle possible to compute the nCC and sAP independently for each experiment and study their distribution over some variate of interest. However, discussion is eased by applying one of various ways of summarization.

(26) consider three summarization methods. These are ‘average’, ‘normalized’, and ‘combined’.

Average This method summarizes by computing the average value of the performance metric of interest for a variate of interest.

Normalized This method standardizes the performance of each experiment by subtracting the mean performance of all methods applied to this experiment and dividing by the standard deviation of all methods’ performance values. For summarization, these standardized scores are then averaged.

Combined This method unites the underlying statistics of the experiments to yield super-experiment statistics. From these super-experiment statistics the final performance values are computed.

(26) report few qualitative differences among these three methods of summarizing, except for that averaging of nCC and sAP tends to reward methods that make no prediction on many datasets. In light of this, we use the ‘combined’ method for summarization.

SUPPLEMENT FOR EVALUATION OF MOTIF DISCOVERY PERFORMANCE ON SYNTHETIC DATA

Bugs in published motif discovery methods

Our evaluation revealed bugs in two motif finders: CMF and MoAn.

Off-by-one error in CMF CMF failed to report motif occurrences matching the found motif that begin at the first position of a sequence, or end on the last position of a sequence. The authors of CMF were quick to provide a patch for this off-by-one programming mistake.

Faulty random number generation in MoAn The problem we found with MoAn was due to faulty integer random number generation. The routine used by MoAn is shown in supplementary figure S10A. It is supposed to generate integers greater or equal to 0 and less than `max`. First, an integer x between 0 and `RAND_MAX`$= 2^{31} - 1 = 2147483647$ is generated using the system routine `rand()`. `max` is cast to `float` and multiplied with x which is implicitly cast to `float` for this. The product is subsequently divided by `RAND_INT`+1, a value strictly greater than x, thus supposedly yielding a value that is strictly less than `max`. Finally, the resulting floating point number is cast to `int` by truncating the fractional part.

However, this routine generates values that are equal to `max` with a probability of $\frac{64}{2147483647}$ or about 2.98×10^{-8}. The reason for this is that variables of type `float` lack resolution to represent values that are close to, but less than 1. In particular, all division results that fall above the largest representable number less than 1 are rounded to one. The consequence is that instead of yielding numbers between 0 and $n-1$, the routine sometimes returns n. As such numbers are used by MoAn to index arrays, segmentation violations may occur, and MoAn aborts. Although such events are individually relatively rare, MoAn generates a lot (millions) of random numbers per run, and thus the problems may occur with a frequency in the percent range. We fixed this problem by modifying the routine as shown in supplementary figure S10B.

Additional results for motif discovery performance on synthetic data

Supplementary figure S11 provides further motif discovery performance metrics on the three sets of experiments with synthetic data for the methods considered in in figure 2. This includes, in addition to nCC, which is already displayed in figure 2, the motif discovery performance metrics sSn, sPPV, sAP, and sF_1.

Motif discovery performance of additional methods

We evaluated the motif discovery performance of all objective functions implemented in Discrover, as well as further published methods on the three sets of experiments. Supplementary figure S12 shows the nCC of all considered methods, including those of figure 2. These supplementary results show that usage of MCC, MICO, MMIE, and DLOGL as objective function in Discrover all yield comparable results, with DFREQ performing worse.

Additional evaluation of DREME During the review process of this manuscript, and updated version of DREME became available. Our initial evaluation was based on the earlier version that lacked a dedicated single-strand mode suitable for RBP analysis. The results of the initial analysis using the double-stranded mode are labelled “DREME DNA” in supplementary figure S12. The later analysis using the updated version in the single-stranded mode is labelled “DREME RNA”. Motif discovery performance is practically identical between the two analysis modes.

By default, DREME reports motifs until the last reported motif fails to meet the E-value threshold (0.05). The above-mentioned evaluations of DREME use a command line switch to instruct DREME to only provide the first motif. To establish whether this reduced motif discovery performance on our synthetic datasets, we also ran DREME in the default mode—letting it reporting motifs until the E-value threshold was met—and subsequently selecting the best scoring one according to DREME’s objective function. This evaluation is labelled “DREME RNA*” in supplementary figure S12, and did not increase motif discovery performance over “DREME RNA”.

We also sought to establish whether using DREME might be a viable alternative to our own seeding method Plasma. Thus we combined DREME with Discrover, with DREME run in the single-stranded RBP analysis mode to discover one seed, on which subsequently an HMM is seeded and further optimized by Discrover. This approach is labelled “MICO-DREME” in supplementary figure S12. It yielded very nearly the same motif discovery performance as when using our own seeding method Plasma to seed HMMs.

Additional evaluation of MoAn As described in the main manuscript, the default number of 3×10^7 iterations used by MoAn made it infeasible to evaluate performance on the decoy dataset. Supplementary figure S12 presents the partial motif performance results of MoAn with the default number of iterations on the basic and 3’UTR datasets, labelled simply “MoAn”. Labelled as “Moan-3M” is the evaluation of MoAn’s motif discovery performance using only 3×10^6 iterations, that is also included in figure 2. As is visible, motif discovery performance increases when more iterations are done.

Runtime of motif discovery methods

Supplementary figure S16 gives the total runtime of the evaluated motif discovery methods on the three sets of experiments. Note that the vertical axis shows runtime in hours on a logarithmic scale. The evaluations were done on an Intel® Xeon® E5645 CPU running at 2.40GHz with 12 CPU cores.

Discrover and its seeding method Plasma may make use of multi-threading capabilities of modern CPUs. Thus, two time measurements are given for each evaluation, one for single-threaded (labelled “ST”) and one for multi-threaded execution.

Overall, there is considerable variation in the run times of the considered motif discovery methods, with the slowest one taking between 226 and 932 times longer than the fastest one.

The order of the methods’ runtimes is relatively similar across the three sets of experiments. As is visible from the figure, using multiple threads Plasma is faster than any other method. DME is faster than Plasma on the 3’UTR dataset when Plasma uses only one thread. Following Plasma, the next

(A) `#define RAND_INT(max)    (int)    (((float) max) * rand() / (RAND_MAX + 1.0))`
(B) `#define RAND_INT(max)    (int)    (((double) max) * rand() / (RAND_MAX + 1l))`

Supplementary figure S10. Random number generation in MoAn. **(A)** Faulty random number generation routine in MoAn. **(B)** Proposed fix.

Supplementary table T4. Runtime of several motif discovery method on one pair of signal and control sequence sets. We considered one particular motif discovery experiment from the basic dataset with 10 000 signal and 10 000 control sequences each of length 1000 with a motif implantation probability of 1 % at an information content of 14 bit. Times are given as hours:minutes:seconds. The last two columns give average CPU utilization in percent of a single CPU, and wall clock time relative to that of Discrover. Experiments were run on an Intel® Xeon® E5645 CPU running at 2.40GHz with 12 CPU cores. Note: DEME did not finish after more than 74 days, 20 hours. Note: DIPS experienced contention for the used CPU, otherwise wall clock time would be around the same as the CPU time.

Method	Wall clock	CPU time	CPU [%]	Relative wall clock
Discrover	00:01:59	00:08:12	413	1.00
DEME	> 74 days	> 74 days	100	> 54330.59
DIPS	628:27:21	605:10:46	96	19008.43
Dispom	40:09:41	88:09:16	219	1214.73
MoAn-3M	00:45:31	00:45:26	100	22.95
MoAn	08:20:57	08:20:04	100	252.53

fastest methods are DME and "DREME RNA", followed by the slightly slower "DREME DNA".

Only a little bit slower than "DREME DNA" are the various objective function modes of Discrover. Depending on whether multiple, or just a single thread is used, Discrover is faster or slower than MDscan and BioProspector. It is noteworthy that among the objective functions of Discrover, the discriminative ones—with the exception of DFREQ—are faster than the generative one using the Baum-Welch algorithm. Aside from being slower, DFREQ is also showing worse motif discovery performance than the other discriminative objective functions of Discrover (see supplementary figure S12).

Again with the exception of DFREQ (and BW on the decoy datasets), but regardless of whether using multiple threads or not, Discrover is considerably faster than the other published discriminative motif discovery methods CMF, FIRE, DECOD, and MoAn-3M.

Note again, that the default number of 3×10^7 iterations for MoAn would imply a roughly ten-fold higher runtime than that of "MoAn-3M" which uses only 3×10^6 iterations.

The runtime of "MICO-DREME" on the 3'UTR datasets is lower than that of MICO because unlike our Plasma seeding method, DREME for some datasets does not return a motif. Particularly, no motif is reported when DREME does not find

Supplementary table T5. Motif discovery performance. nCC: nucleotide-level Matthews correlation coefficient, %: nCC in percent of recognizability. NA: not available. Numbers are plotted in supplementary figure S12.

	Basic		3'UTR		Decoy	
	nCC	%	nCC	%	nCC	%
Recognizability	0.68	100	0.68	100	0.59	100
BioProspector	0.43	63	0.43	63	0.21	36
CMF	0.40	59	0.45	66	0.27	45
DECOD	0.42	62	0.34	51	0.42	72
DME	0.48	71	0.41	60	0.44	75
DREME DNA	0.56	83	0.59	86	0.52	88
DREME RNA	0.56	83	0.59	86	0.53	90
DREME RNA*	0.56	83	0.59	87	0.53	90
FIRE	0.39	58	0.40	59	0.37	62
MDscan	0.15	23	0.19	28	0.09	15
MoAn-3M	0.60	89	0.62	91	0.50	85
MoAn	0.63	92	0.64	94	NA	NA
Discrover - BW (MICO)	0.67	98	0.51	76	0.55	93
Discrover - BW	0.67	98	0.51	74	0.49	83
Discrover - DFREQ	0.61	89	0.59	87	0.30	51
Discrover - DLOGL	0.66	97	0.66	97	0.58	97
Discrover - MCC	0.66	97	0.66	97	0.56	95
Discrover - MICO-DREME	0.65	96	0.66	97	0.57	96
Discrover - MICO	0.66	97	0.66	97	0.57	96
Discrover - MMIE	0.66	98	0.67	98	0.57	96
Plasma	0.63	93	0.61	90	0.56	95

a motif that meets its significance threshold. Whenever this is the case, no subsequent optimization happens for “MICO-DREME”, thus fewer HMMs are optimized, which explains the reduced runtime. On the other hand, as the figure shows, DREME itself is somewhat slower than Plasma, which is why “MICO-DREME” is slightly slower than “MICO” on the basic and decoy datasets.

Interestingly, our Baum-Welch learning method BW is slower than most of our discriminative ones. This is in spite of the fact that line searching for gradient optimization of the discriminative objectives evaluates the objective function (and simultaneously its gradient) multiple times per iteration, typically three times. Also, within our hybrid learning approach the Baum-Welch algorithm is used to reestimate the probabilities for state transitions and background state emissions (see section Hybrid Learning in main manuscript). The increased runtime of the pure BW approach, then, is due to the higher total number of iterations needed until convergence, as compared to that needed for convergence in hybrid learning for discriminative objectives.

Effect of significance filtering on motif discovery performance

Supplementary figure S17 shows the effect that significance filtering based on MICO has on the motif discovery performance. It considers the effect on the performance of signal-only, generative HMM parameter learning, and on that of MICO-based, discriminative HMM parameter learning. For the signal-only case, seeds are determined in two ways: (I) either by frequency in the signal-data alone (“BW”), or (II) in a discriminative way by evaluating MICO on the signal and control data (“BW (MICO)”). As is evident from the figure, the effect of discarding motifs that are not significantly associated with the signal/control distinction is large in case HMM parameters are trained only using the signal data, i.e. for “BW” and for “BW (MICO)”. On the other hand, when HMM parameters are trained by MICO, then the effect of significance filtering based on MICO, is not very pronounced.

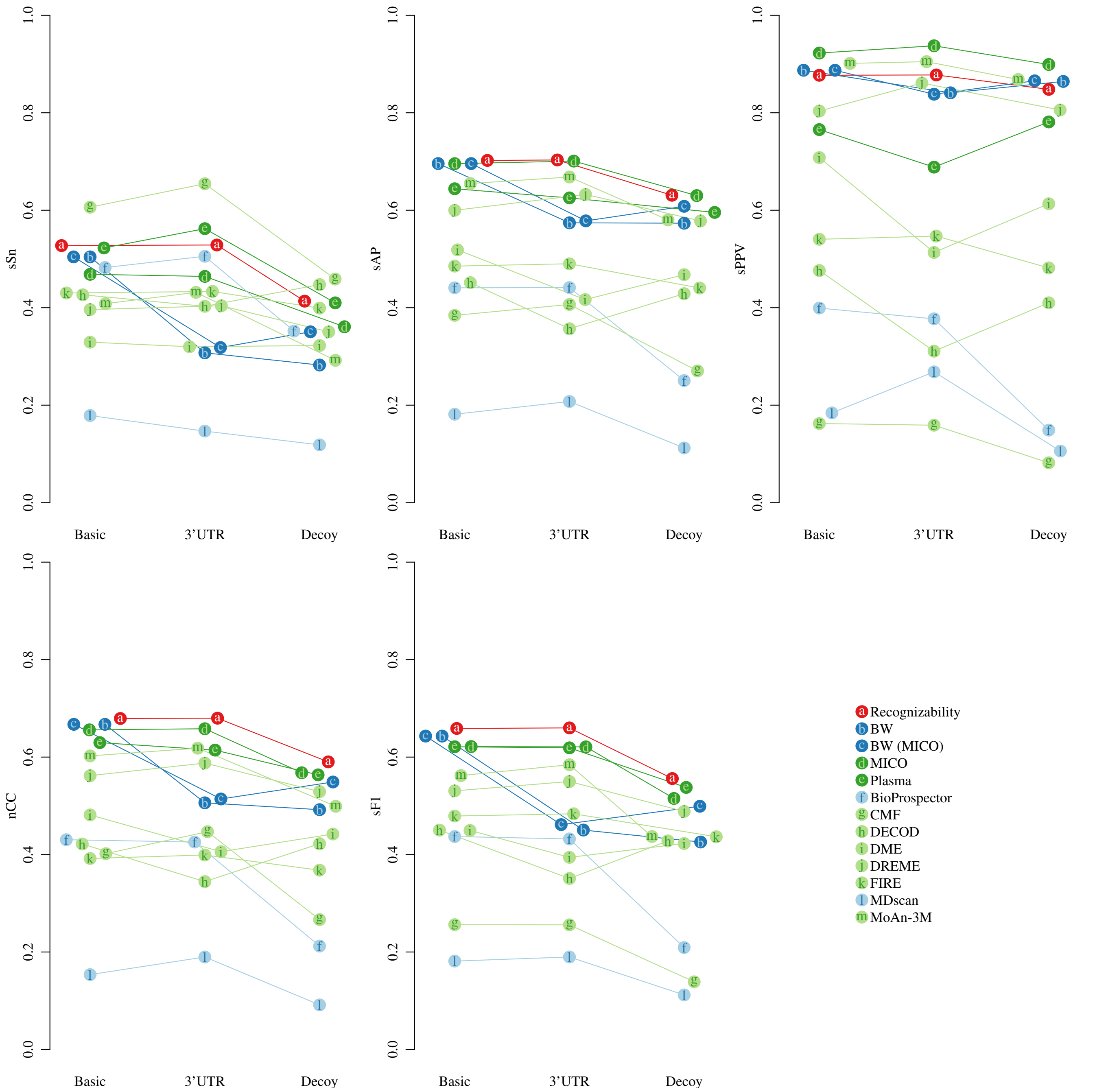

Supplementary figure S11. Summarized motif finding performance of various methods on three synthetic datasets measured by the nucleotide-level Matthews correlation coefficient (nCC), average site performance (sAP), site sensitivity (sSn), and site positive predictive value (sPPV), as well as the sF_1-score. See (**89**)–(**93**) for definition of the metrics. Recognizability (red) serves as reference. Blue denotes signal-only motif learning methods, while green denotes discriminative motif discovery methods. Dark letters and light background denote published motif finding methods, light letters and dark background denote motif finding with objective functions implemented in Discrover. BW: Baum-Welch training of HMMs seeded with the most frequent IUPAC motifs of degeneracy maximally 2, BW (MICO): Baum-Welch training of HMMs seeded with IUPAC motifs maximizing MICO. Plasma: IUPAC RE motif optimization with MICO as objective function.

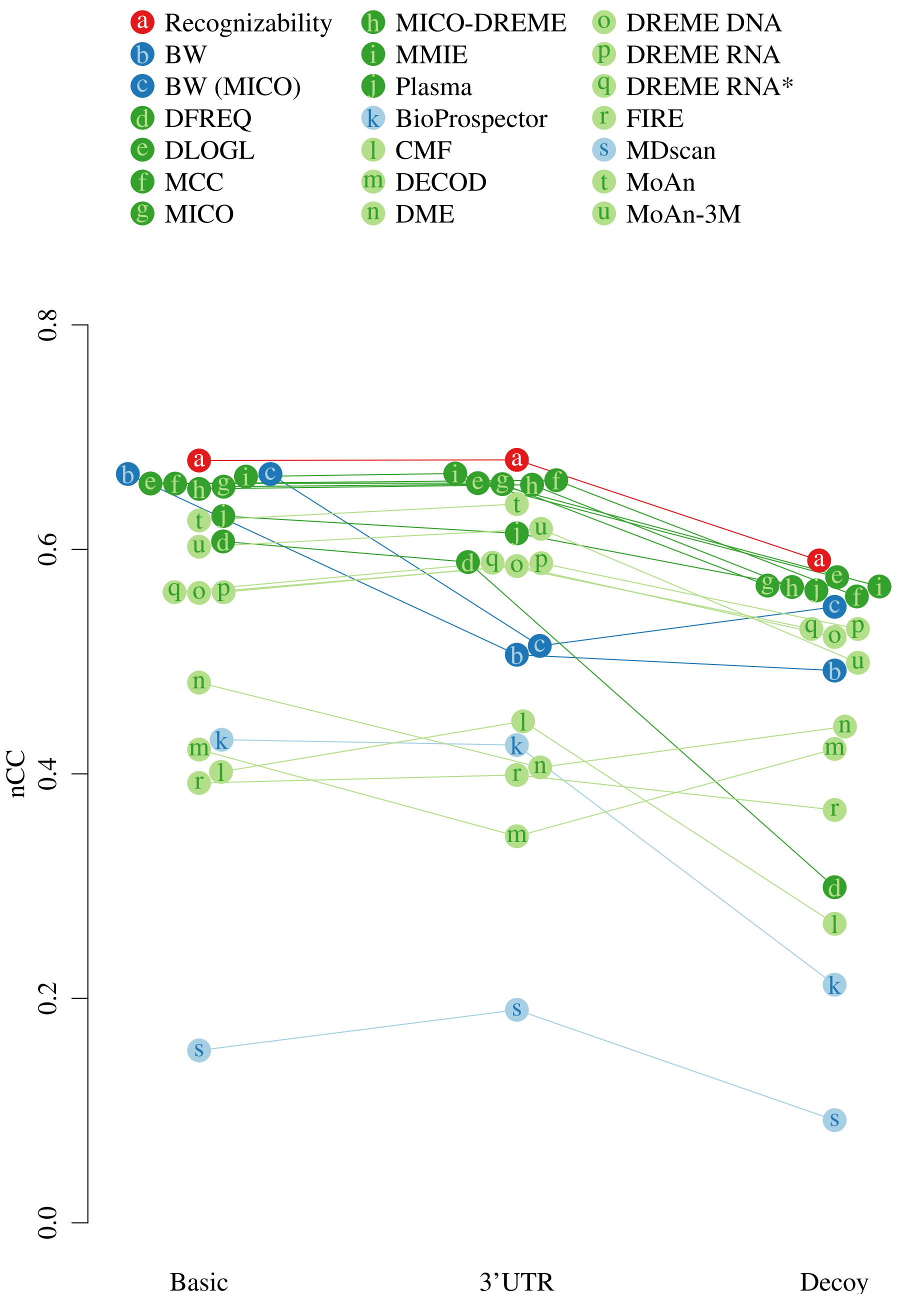

Supplementary figure S12. Summarized motif finding performance of methods on three synthetic datasets measured by the nucleotide-level Matthews correlation coefficient (nCC). Recognizability (red) serves as reference. Blue denotes signal-only motif learning methods, while green denotes discriminative motif discovery methods. Dark letters and light background denote published motif finding methods, light letters and dark background denote motif finding with objective functions implemented in Discrover. BW: Baum-Welch training of HMMs seeded with the most frequent IUPAC motifs of degeneracy maximally 2, BW (MICO): Baum-Welch training of HMMs seeded with IUPAC motifs maximizing MICO. Plasma: IUPAC RE motif optimization with MICO as objective function. MoAn-3M: MoAn with 3×10^6 iterations. MoAn: MoAn with 3×10^7 iterations; note that it was infeasible for us to evaluate the decoy dataset in this case. MICO-DREME: DREME provides seeds, on which HMMs are seeded and further optimized for MICO by Discrover. DREME DNA: DREME in double-stranded motif analysis mode, suitable for DNA-binding protein analysis; providing one seed. DREME RNA: DREME in single-stranded motif analysis mode, suitable for RNA-binding protein analysis; providing one seed. DREME RNA*: DREME in single-stranded motif analysis mode, discovering motifs as long as the E-value threshold is met, of which subsequently the highest scoring one is used for evaluation.

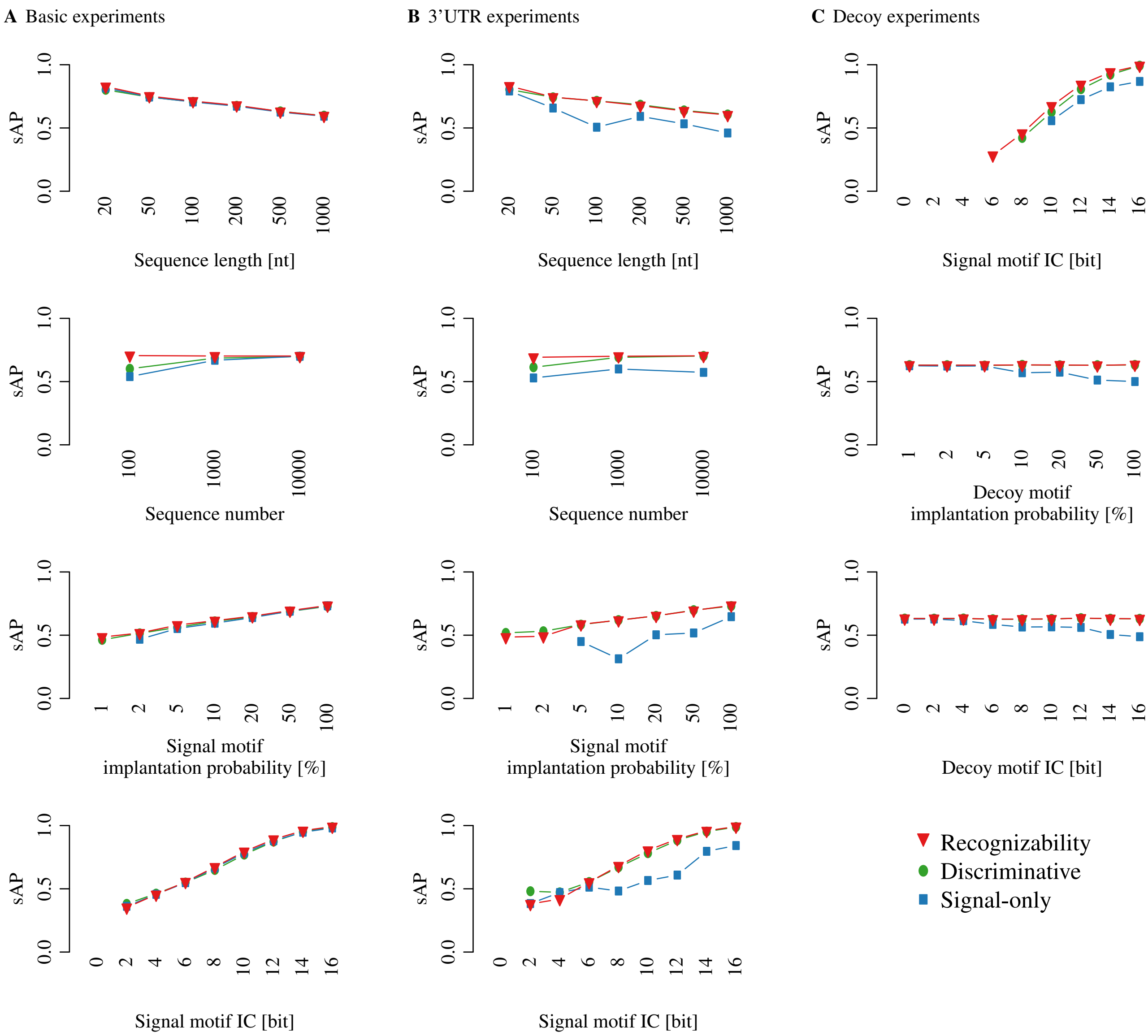

Supplementary figure S13. Motif recognizability and discovery performance measured by average site performance (sAP) on synthetic data in the **(A)** basic, **(B)** 3'UTR, and **(C)** decoy experiments. Note that sAP is not defined when sPPV is not defined (see supplementary figure S15). See legend of figure 3 for further explanations.

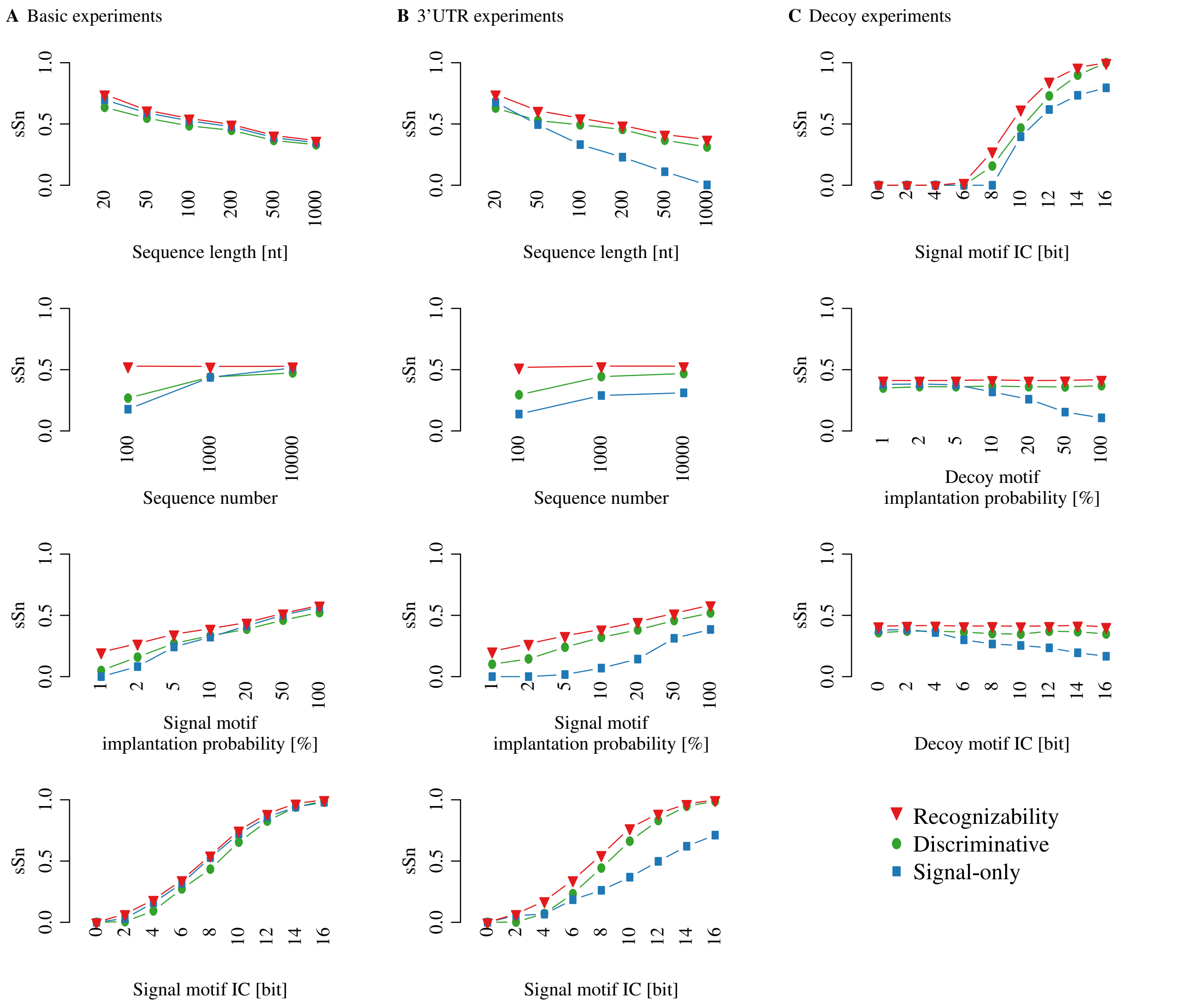

Supplementary figure S14. Motif recognizability and discovery performance measured by site-level sensitivity (sSn) on synthetic data in the **(A)** basic, **(B)** 3'UTR, and **(C)** decoy experiments. See legend of figure 3 for further explanations.

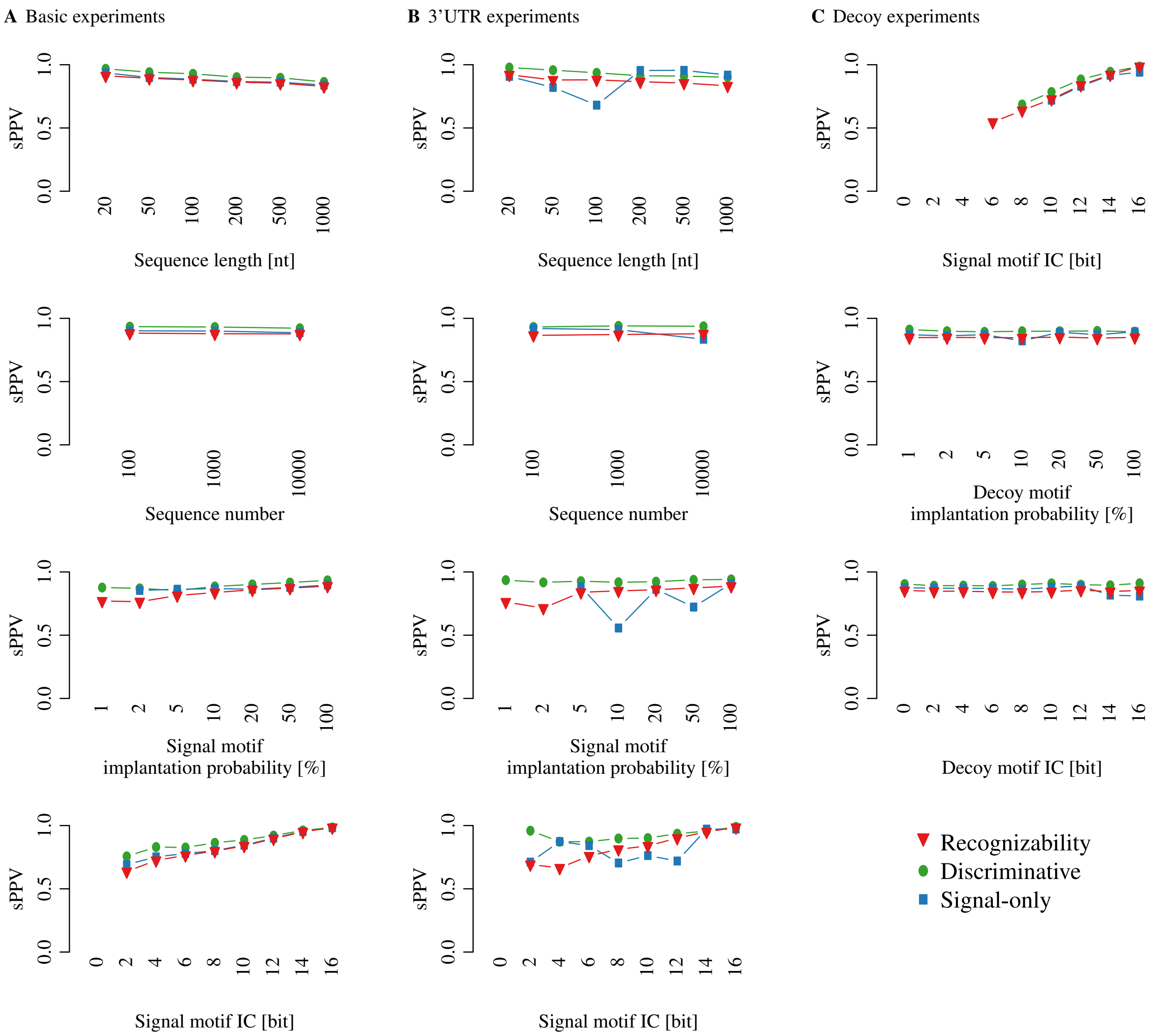

Supplementary figure S15. Motif recognizability and discovery performance measured by site-level positive predictive value (sPPV) on synthetic data in the **(A)** basic, **(B)** 3'UTR, and **(C)** decoy experiments. See legend of figure 3 for further explanations. Note that sPPV is not defined when no motif occurrences are predicted, e.g. for low signal motif IC values in **(C)**.

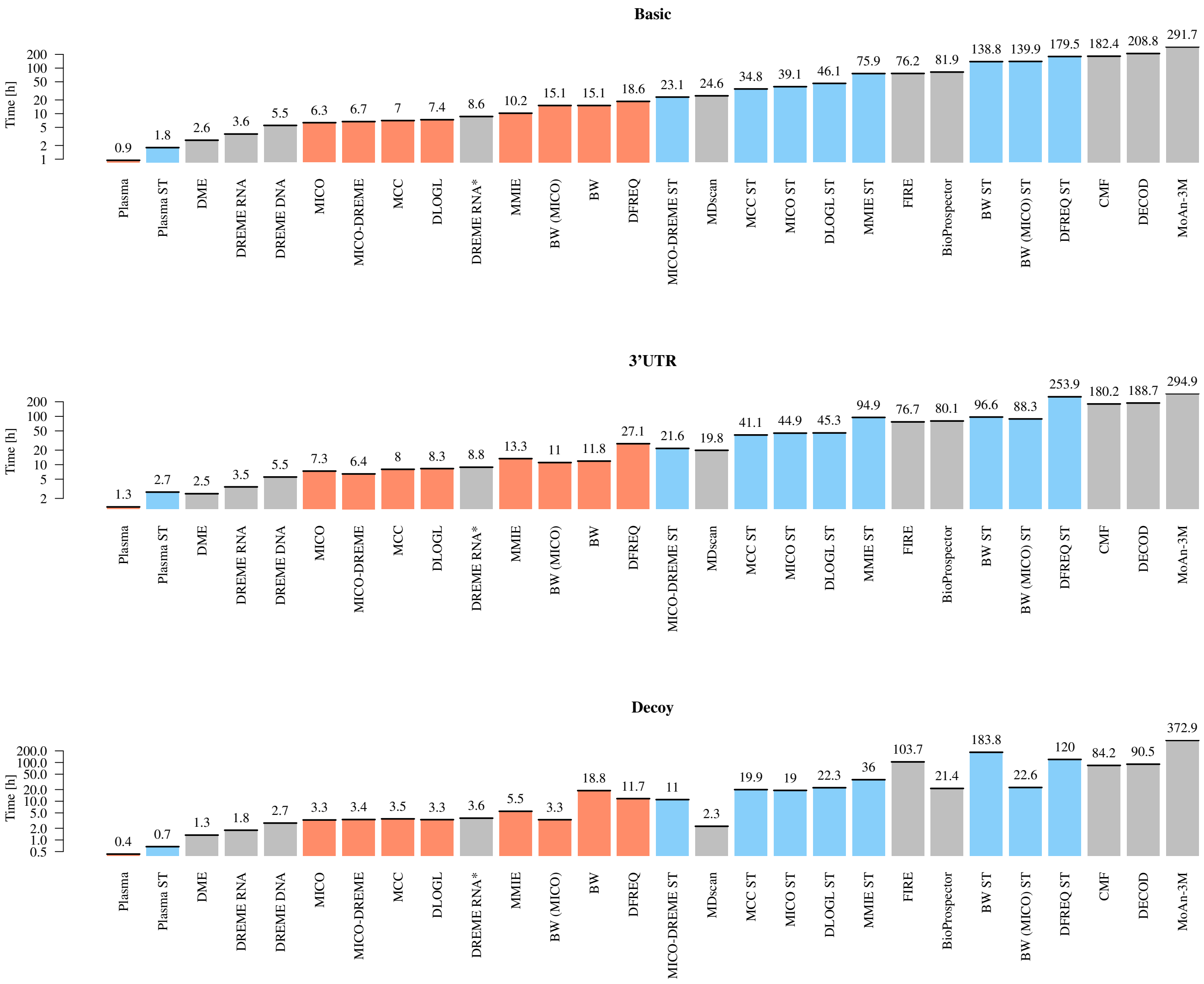

Supplementary figure S16. Runtime of motif discovery methods on the three synthetic datasets using an Intel® Xeon® E5645 CPU running at 2.40GHz with 12 CPU cores. Note that the runtime is given on a logarithmic scale. Grey bars denote published methods. As Discrover can utilize multiple threads, we include two time measurements: orange bars denote multi-threaded runtime (wall clock time), blue bars denote single-threaded runtime (CPU time). Note that the runtime of MoAn with the default number of iterations, which we performed for the basic and 3'UTR experiments is not included, as it was run on a different compute, and thus the runtimes are not comparable.

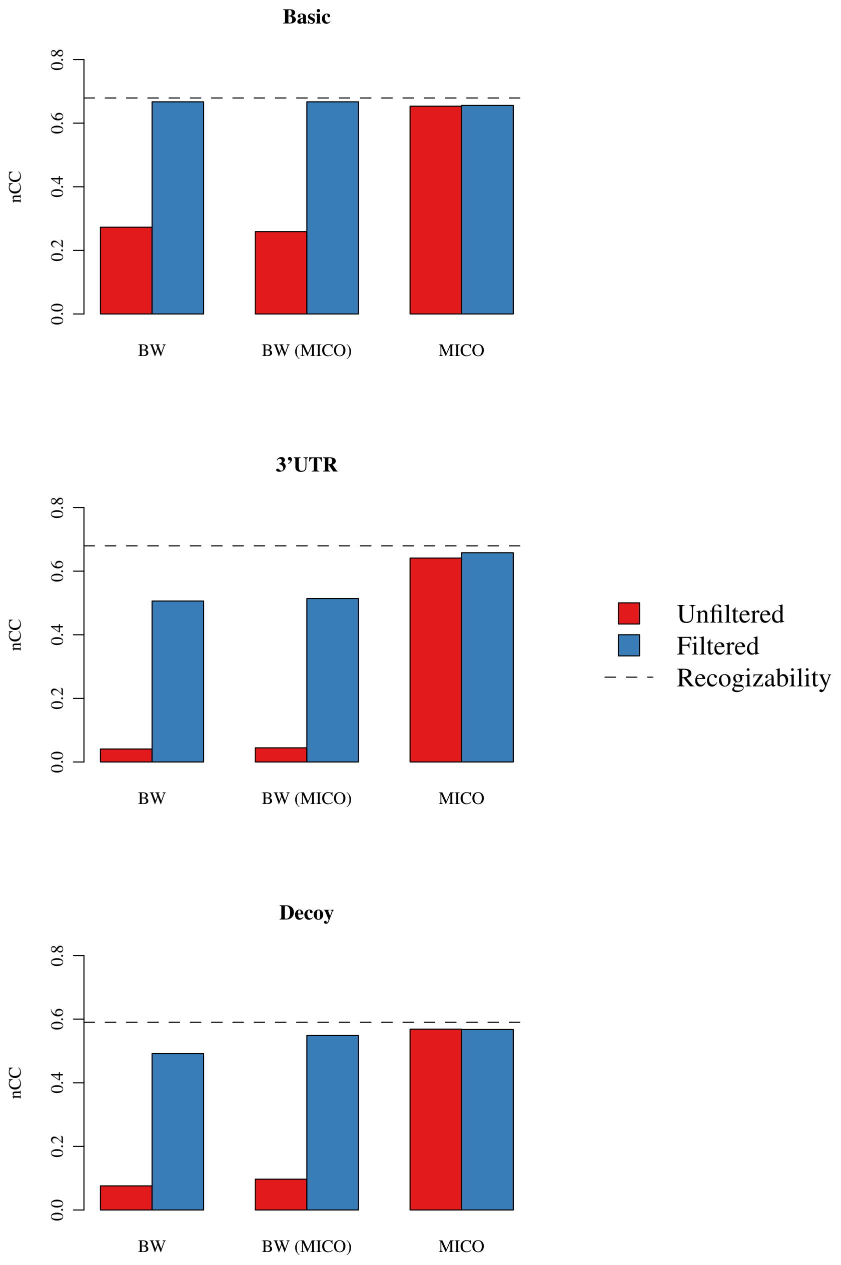

Supplementary figure S17. Effect of significance filtering on motif discovery performance measured by the nucleotide-level Matthews correlation coefficient (nCC). Blue and red bars give motif discovery performance respectively with or without discriminative filtering for significance of association after learning. For reference, the nCC of recognizability is indicated by the dashed line. Significance filtering is done by evaluating MICO on the signal and control example sequences, computing the associated p-value, correcting for multiple testing, and discarding motifs failing the significance threshold. BW: signal-only learning of HMM parameters with the Baum-Welch algorithm, using as seeds the 8mers of degeneracy at most 2 that are most frequent in the signal data. BW (MICO): signal-only learning of HMM parameters with the Baum-Welch algorithm, using discriminative seeds that maximize MICO for IUPAC regular expressions on the signal and control data. MICO: Discriminative learning of HMM parameters by MICO, with discriminative seed determined by optimizing MICO.

ADDITIONAL RESULTS FOR PUF FAMILY RBP

To investigate the disparity of conclusions regarding the second half of the PUM2 motif between our analyses of RIP-chip and PAR-CLIP data, we first investigated the influence of the choice of objective function. We thus repeated the analysis of the PUF RBP family data using MMIE as objective function. We initialized HMMs with seeds of length 7-12 nt determined by MICO, optimized the HMM parameters for MMIE, and selected the HMM with highest MMIE score. The results are tabulated in supplementary table 6B. We found that MMIE identifies longer variants of the MICO motifs which include positions with relatively low information content. This is because for MMIE we lack a comparable significance correction for motif length as is available for MICO. The motifs identified by MMIE frequently have slightly lower information content than the MICO motifs, and occur more frequently than those of MICO. This observation is in line with our findings on the synthetic datasets that MMIE yields higher sSn and lower sPPV than MICO. Also for MMIE we observe a disparity between the RIP-chip and PAR-CLIP analysis results regarding the second half of the motif.

To see what generative signal-only learning might yield, we applied the Baum-Welch algorithm to optimize the IUPAC seed sequence `NNUGUANAUANN` on the full PUF RBP family signal datasets. While this successfully determines models with higher likelihood than those for MICO or MMIE, it did not yield useful motifs (supplementary table 6C). Although we used a seed similar to the motifs discovered by discriminative analysis in all but the Puf1 and Puf2 datasets, the PRE is mostly replaced by unspecific sequence. Only in the cases of Puf3, Puf4, and PUM2 PAR-CLIP data do some traces of the motif remain. However the motifs incorporate so much background characteristic that they occur in more than half of the respective control sequences, which are either random shuffles or all non-target 3'UTRs.

Next, because the covered regions of PAR-CLIP data are much shorter than the 3'UTR sequences used for the RIP-chip data (supplementary figure S18), we performed a dilution analysis of the PUM2 PAR-CLIP data by embedding the real sequences in increasingly larger, synthetically generated sequence context (see paragraph below). We thus analyzed the original PUM2 data (mean length 35.0 nt), as well as variants padded to minimum lengths of 64, 128, 256, 512, and 1024 nt. Then, for the objective functions MICO and MMIE, we optimized parameters of HMMs seeded on `NNUGUANAUANN`, and the results are shown in supplementary figures S19 and S20. We found that with increasing sequence context size the discriminative motif analyses of PAR-CLIP data embedded in random sequence become more alike to the results of our RIP-chip data analyses. The dilution has the effect of yielding higher information content, particularly on the second half of the motif.

As another line of evidence we turned to word count analysis. Scatter plots of frequencies of sequences that have a given word in supplementary figures S21-S24 reveal that the longer sequence sizes of the array data compress the variability of word frequencies between signal and control sequences. In order to separate independent contributions due to central and neighboring words we employed a simple progressive algorithm to determine relevant words (see supplementary figure S25 and paragraph below for details). We found a great variety of independently discriminative, `UGUANNNN` conforming words in the PAR-CLIP data among the 50 most discriminative 8mers, while strong differential enrichment in the array data is limited to `UGUAAAUA`, `UGUAUAUA`, and `UGUACAUA`. The IUPAC motif UGUAHAUA, which comprises these three words is occurring in more than half of the PUM1 and PUM2 array data signal sequences, and in less than 15% of corresponding control sequences (supplementary figure S26). In contrast, for the PAR-CLIP data, this motif is only present in 19.7% and 2.3% of signal and control sequences, respectively.

Given the higher variety of weaker variants that independently contribute to MICO in the PAR-CLIP data, and that by diluting the PAR-CLIP data the results of discriminative motif discovery agree with those of the array data, we surmise that the smear is not observed in the array data due to the large length of 3'UTR sequences precluding discovery of weakly affine variants. It is likely that this shadowing of the weaker variants in the array data is a consequence of our choice of feature, that a sequence is considered a target if it has at least one occurrence of a motif.

Dilution analysis of PUM2 PAR-CLIP data

We performed a dilution analysis of the PUM2 PAR-CLIP data by embedding the real sequences in increasingly larger, synthetically generated sequence context. Specifically, for each signal dataset, we extracted dinucleotide frequencies, and generated for each signal sequence shorter than the desired length flanks of the appropriate size, as well as a control sequence of equal total size. We thus analyzed the original PUM2 data (mean length 35.0 nt), as well as variants embedded to minimum lengths of 64, 128, 256, 512, and 1024 nt. Then, for the objective functions MICO and MMIE, we optimized parameters of HMMs seeded on `NNUGUANAUANN`. The results are shown in supplementary figures S19 and S20.

Word based discriminative analysis

We performed a simple word based analysis on the PUM1 and PUM2 datasets, that we call corenmer analysis.

Algorithm 1 corenmer analysis

Require: word length n, number of words to determine k, data X, objective function f, alphabet $\mathcal{A}$
Ensure: $(w_i)_{i=1,\dots,n}$ the k most relevant n-mers
  for $i = 1 \rightarrow k$ **do**
    $w_i \leftarrow \mathrm{argmax}_{w \in \mathcal{A}^n} f(w, X)$
    $X \leftarrow \mathrm{mask}(X, w_i)$
  end for

The algorithm determines the k most relevant words of length n on the data X according to some objective function f. This is done by progressively identifying the most relevant word, and masking its occurrences in the data, before identifying further words. The objective functions must be based on discrete counts, and we typically employed MICO. As the objective function for a word may change after masking of occurrences of an overlapping word, we refer to the objective function value at which a word is selected as its

Supplementary table T6. Motif discovery results for datasets of PUF RBP family members. Columns N_S and N_C give the number of sequences in the signal and control sequence set, respectively. IC: information content, log-L: log-likelihood, S and C: expected relative frequency of signal and control sequences with at least one occurrence of the motif, log-p: MT-corrected log-p value. **(A)**: MICO is used as objective function in discriminative motif analysis to find seeds of length 7-12 nt, and to optimize HMM parameters. Motifs are selected by MT-corrected p-value. **(B)**: discriminative motif analysis with MMIE. MICO was used to find seeds, and HMM parameters were optimized by MMIE. Motifs are selected by MMIE score. **(C)**: Baum-Welch algorithm is applied to the seed `NNUGUANAUANN`. Note that the analysis of the FBF-1 data in the paper was done by splitting the data into 15 sets, as described in the methods part, while for the analysis results presented here the data was split as indicated in the table. Data sources: [a] (29), [b] (30), [c] (31), [d] (32), [e] (33), [f] (34).

A

Protein	N_S	N_C	Motif	IC [bit]	log-L	S [%]	C [%]	MICO [bit]	log-p
PUF1 [a]	32	5180	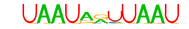	19.4	−6862	40.8	0.7	64.3	0
PUF2 [a]	124	5088	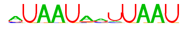	18.9	−28 993	33.9	1.0	150.6	−47.3
PUF3 [a]	68	5144	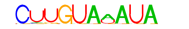	17.8	−10 184	52.1	3.5	103.7	−24.6
PUF4 [a]	184	5028	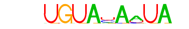	15.2	−32 049	47.8	4.2	207.4	−101.9
PUF5 [a]	156	5056	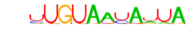	16.9	−32 416	35.7	2.3	146.6	−49.5
FBF-1 [b]	3294	10 096	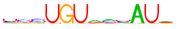	12.5	−970 238	20.9	5.2	462.4	−264.0
Pumilio [c]	834	12 135	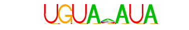	14.5	−780 326	50.7	12.9	448.6	−274.4
PUM1 [d]	836	6320	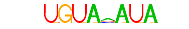	14.5	−2 094 000	61.6	13.1	638.3	−406.1
PUM1 [e]	1401	18 651	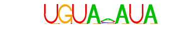	14.5	−3 515 930	49.8	5.3	1375.8	−917.7
PUM2 [e]	565	19 535	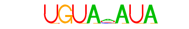	14.4	−1 372 030	56.1	6.2	687.9	−440.5
PUM2 [f]	6916	6916	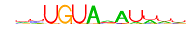	13.5	−327 370	54.1	10.8	2269.7	−1517.7

B

Protein	N_S	N_C	Motif	IC [bit]	log-L	S [%]	C [%]	MICO [bit]	log-p	MMIE
PUF1 [a]	32	5180	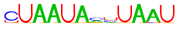	19.3	−6876	38.7	0.8	58.3	0	−145
PUF2 [a]	124	5088	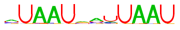	17.3	−29 003	36.2	2.0	130.5	−33.2	−464
PUF3 [a]	68	5144	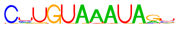	19.0	−10 221	49.3	2.8	104.4	−15.0	−276
PUF4 [a]	184	5028	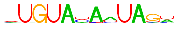	16.8	−32 077	45.6	3.5	208.7	−87.7	−611
PUF5 [a]	156	5056	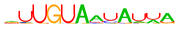	16.2	−32 421	41.4	4.2	139.6	−39.6	−564
FBF-1 [b]	3294	10 096	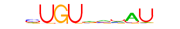	9.8	−970 083	38.9	17.9	415.9	−246.7	−7005
Pumilio [c]	834	12 135	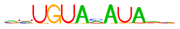	14.8	−780 320	54.7	15.7	441.9	−249.7	−2695
PUM1 [d]	836	6320	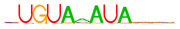	14.3	−2 093 960	65.8	16.4	618.2	−372.2	−1998
PUM1 [e]	1401	18 651	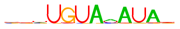	13.4	−3 515 800	59.1	9.0	1386.8	−905.3	−3776
PUM2 [e]	565	19 535	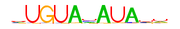	14.2	−1 372 080	58.3	7.9	646.1	−396.5	−1975
PUM2 [f]	6916	6916	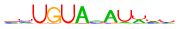	12.9	−326 888	60.5	16.6	2127.8	−1419.3	−7626

C

Protein	N_S	N_C	Motif	IC [bit]	log-L	S [%]	C [%]	MICO [bit]	log-p
PUF1 [a]	32	5180		4.7	−6771	88.9	82.4	0.3	0
PUF2 [a]	124	5088		3.1	−28 733	93.1	89.5	0.9	0
PUF3 [a]	68	5144	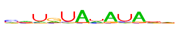	9.0	−10 127	85.3	68.7	6.4	0
PUF4 [a]	184	5028	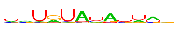	8.8	−31 993	81.3	67.7	11.3	0
PUF5 [a]	156	5056		8.4	−32 377	35.3	38.1	0.3	0
FBF-1 [b]	3294	10 096	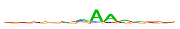	4.3	−963 728	94.0	90.0	36.2	0
Pumilio [c]	834	12 135		2.2	−774 029	99.2	97.8	5.3	0
PUM1 [d]	836	6320		1.2	−2 070 780	99.6	96.8	18.6	0
PUM1 [e]	1401	18 651		2.3	−3 489 370	99.2	96.2	32.2	0
PUM2 [e]	565	19 535		1.2	−1 359 480	95.6	75.0	121.8	−27.2
PUM2 [f]	6916	6916	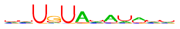	9.9	−324 488	82.0	53.8	934.2	−591.4

residual objective value. The residual objective value is thus an estimate of the independent contribution a word conveys for discriminating two sets of sequences after more important words have been accounted for.

We determined the top 50 words of length 8 on the human PUM1 and PUM2 datasets of (32–34) according to residual MICO. Supplementary figure S25 shows these in the order produced by the algorithm, with the bars indicating how many of the sequences in signal (blue) and control (red) have at least one occurrence of the word. The light parts of the bars indicate which of the sequences have occurrences of any words with higher residual MICO, and thus are potentially already explained by them. It it thus the dark portions of the bars that indicate the novel explanatory contribution of a word when accepting words in decreasing order of residual MICO.

As is visible from that figure, there is greater variety of `UGUANNNN` conforming words in the PAR-CLIP data among the 50 8mers with highest residual MICO, while only `UGUAAAUA`, `UGUAUAUA`, and `UGUACAUA` appear to be strongly differential in the array data.

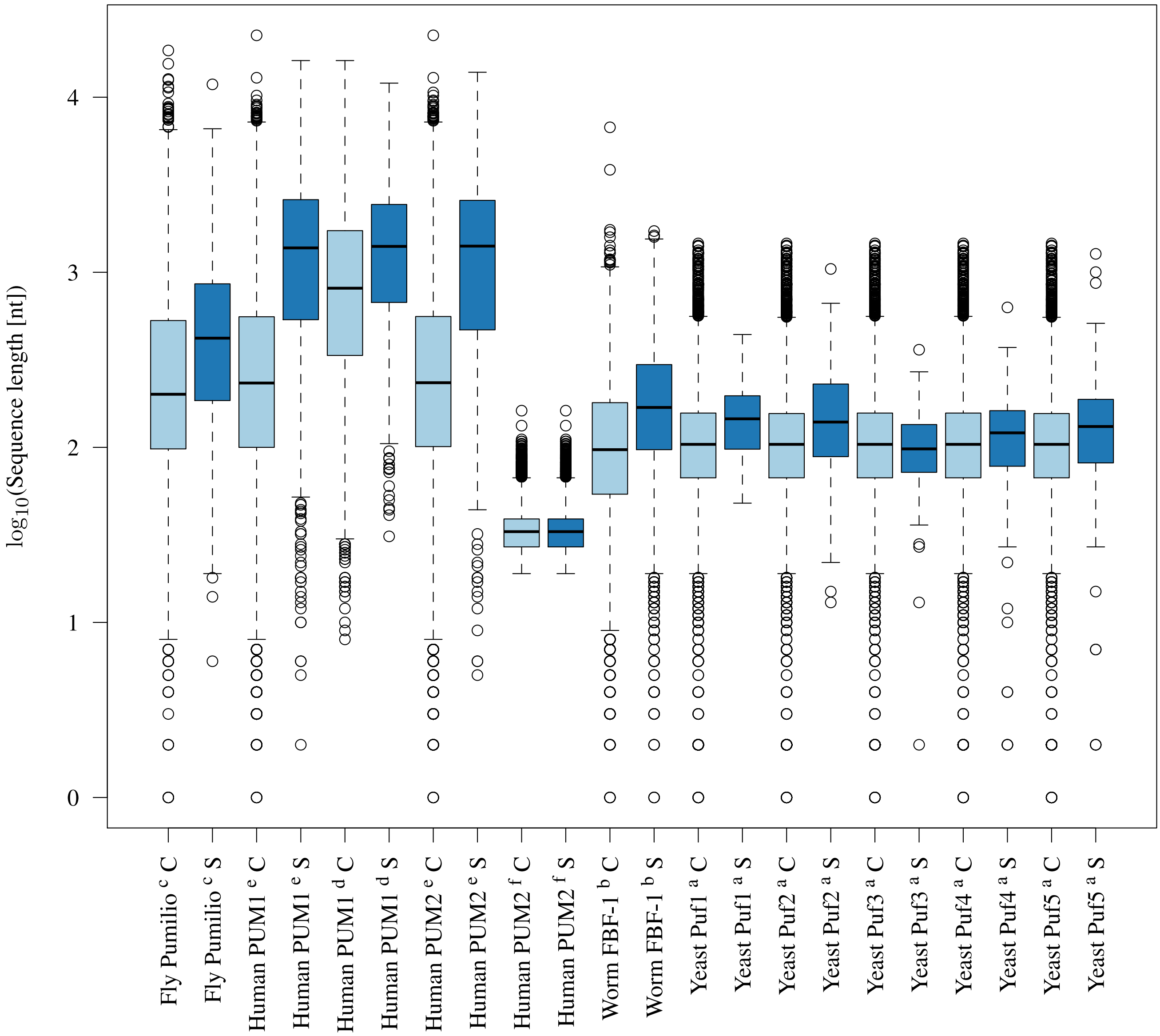

Supplementary figure S18. Boxplot of sequence lengths in the various datasets and their control sets. S: Signal sequences (dark blue). C: Control sequences (light blue). Data sources: [a] (29), [b] (30), [c] (31), [d] (32), [e] (33), [f] (34).

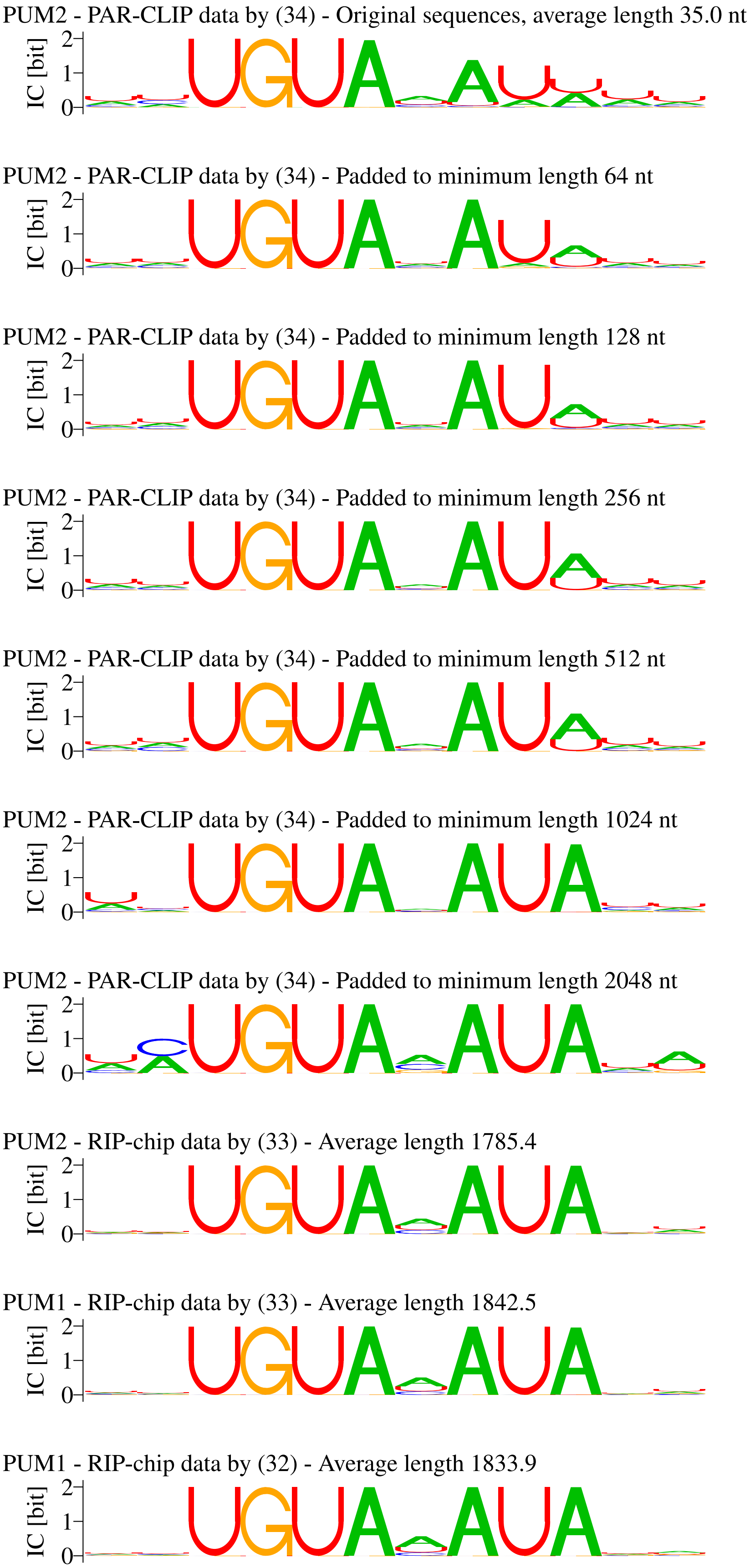

Supplementary figure S19. Dilution analysis of PUM2 PAR-CLIP data of (34) for MICO. Sequences were embedded in increasing amounts of random sequences, varying from top to bottom. HMMs were seeded on the IUPAC word `NNUGUANAUANN` and parameters optimized for MICO.

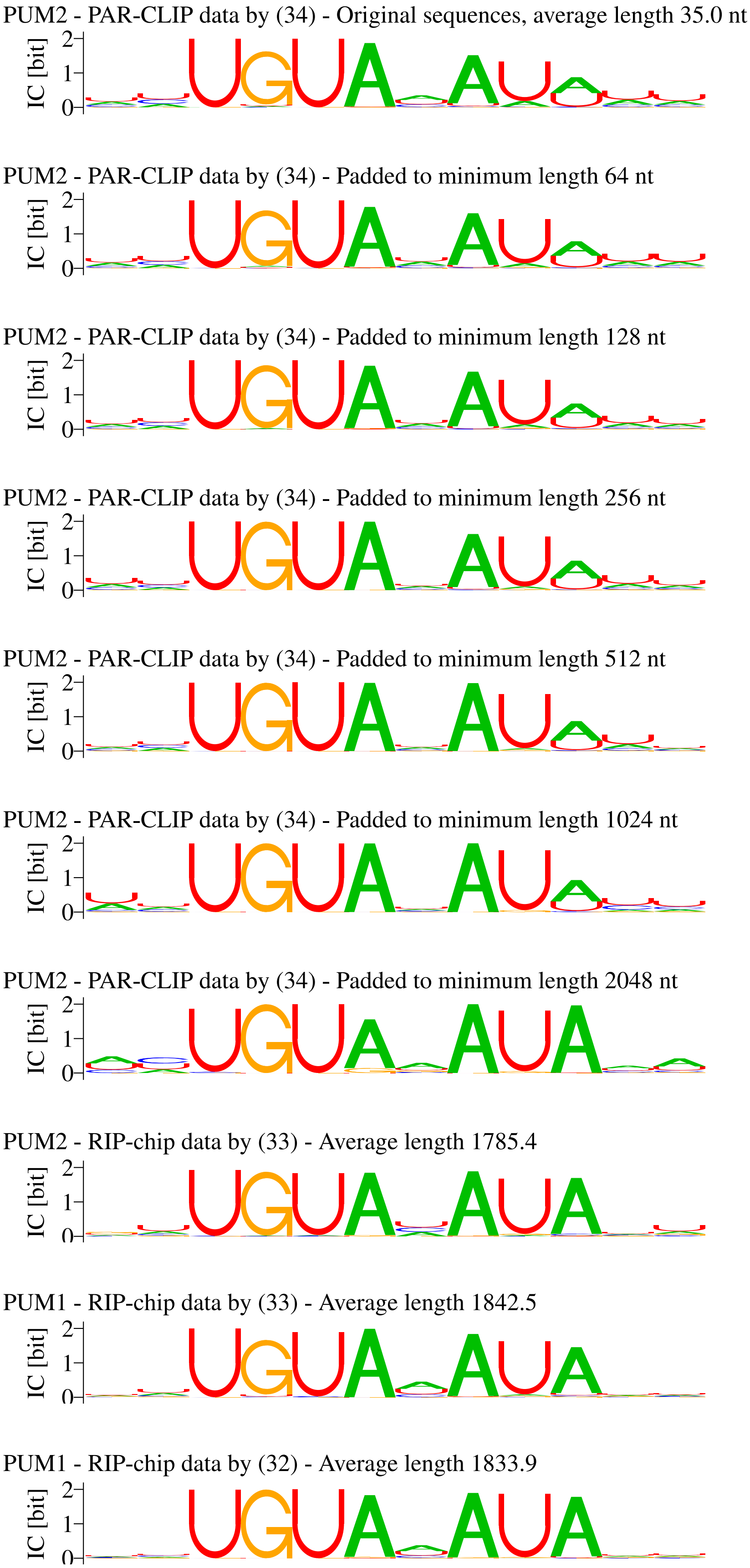

Supplementary figure S20. Dilution analysis of PUM2 PAR-CLIP data of (34) for MMIE. Sequences were embedded in increasing amounts of random sequences, varying from top to bottom. HMMs were seeded on the IUPAC word NNUGUANAUANN and parameters optimized for MMIE.

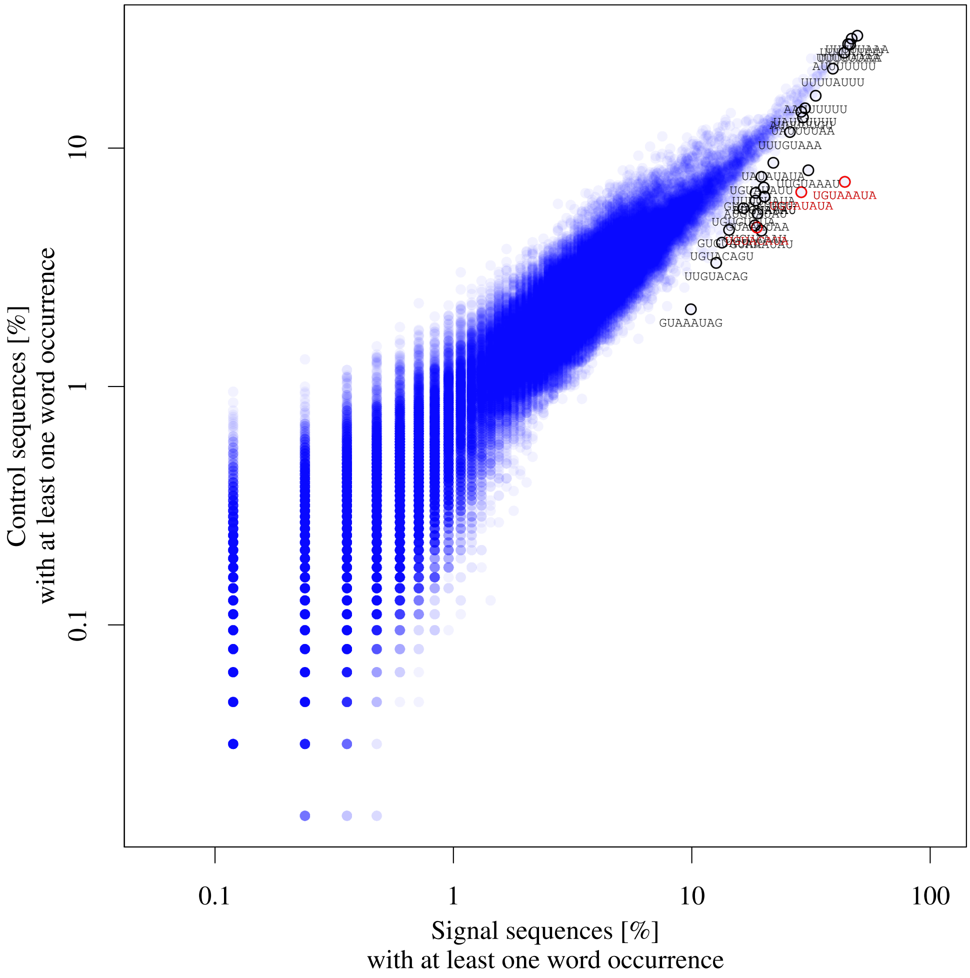

Supplementary figure S21. Scatter plot of percentages of PUM1 (32) signal and control sequences that have a given 8mer. The 30 words with highest marginal MICO are labeled. The top three words according to residual MICO are highlighted in red.

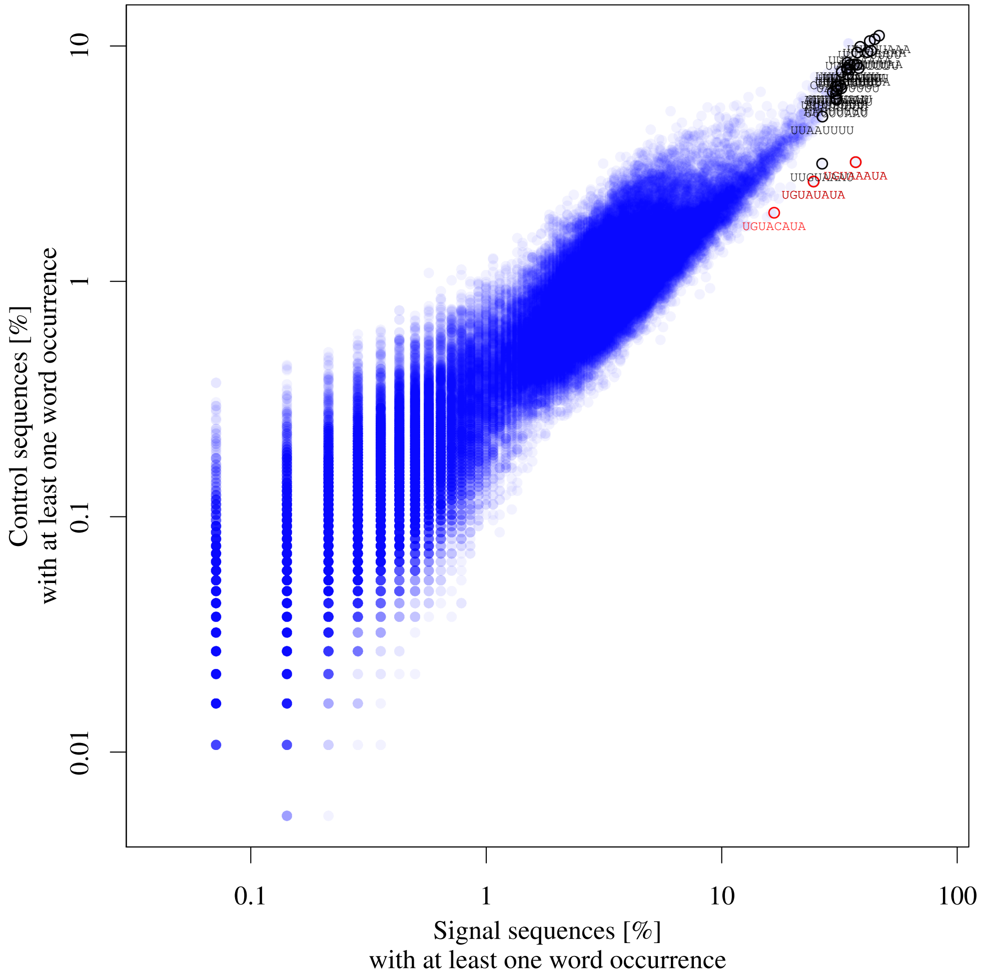

Supplementary figure S22. Scatter plot of percentages of PUM1 (33) signal and control sequences that have a given 8mer. The 30 words with highest marginal MICO are labeled. The top three words according to residual MICO are highlighted in red.

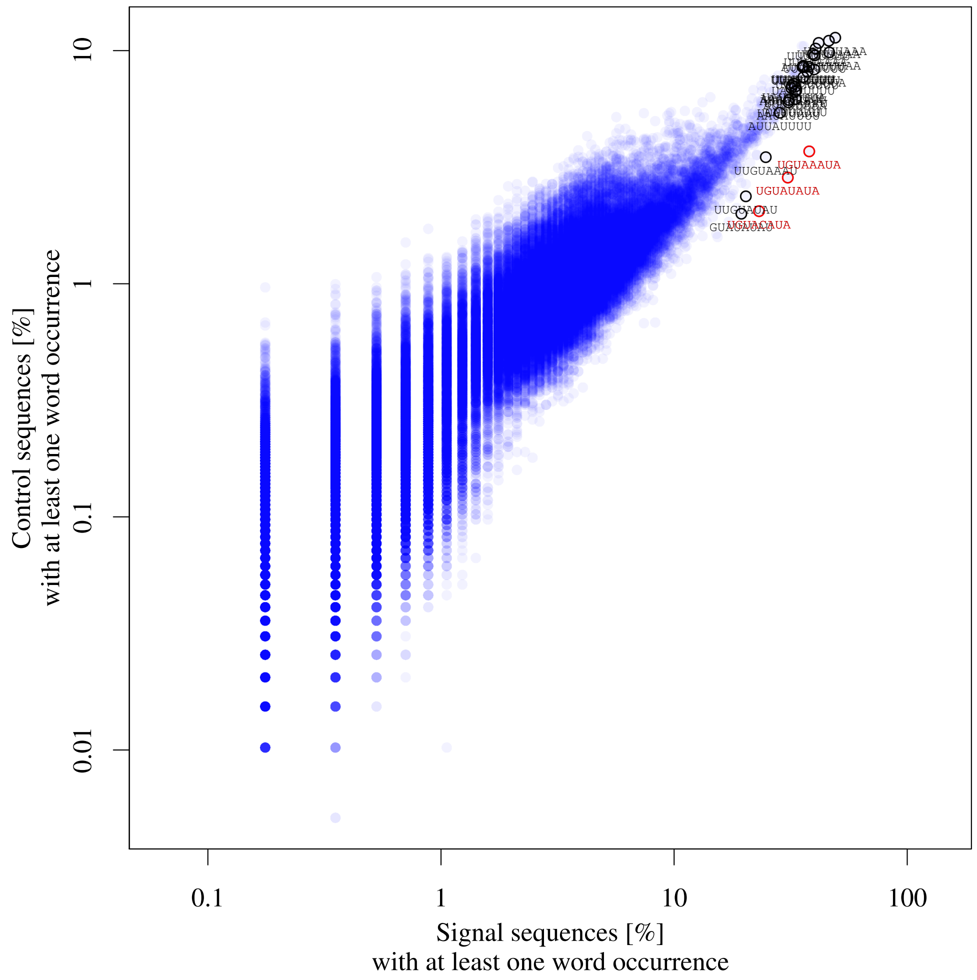

Supplementary figure S23. Scatter plot of percentages of PUM2 (33) signal and control sequences that have a given 8mer. The 30 words with highest marginal MICO are labeled. The top three words according to residual MICO are highlighted in red.

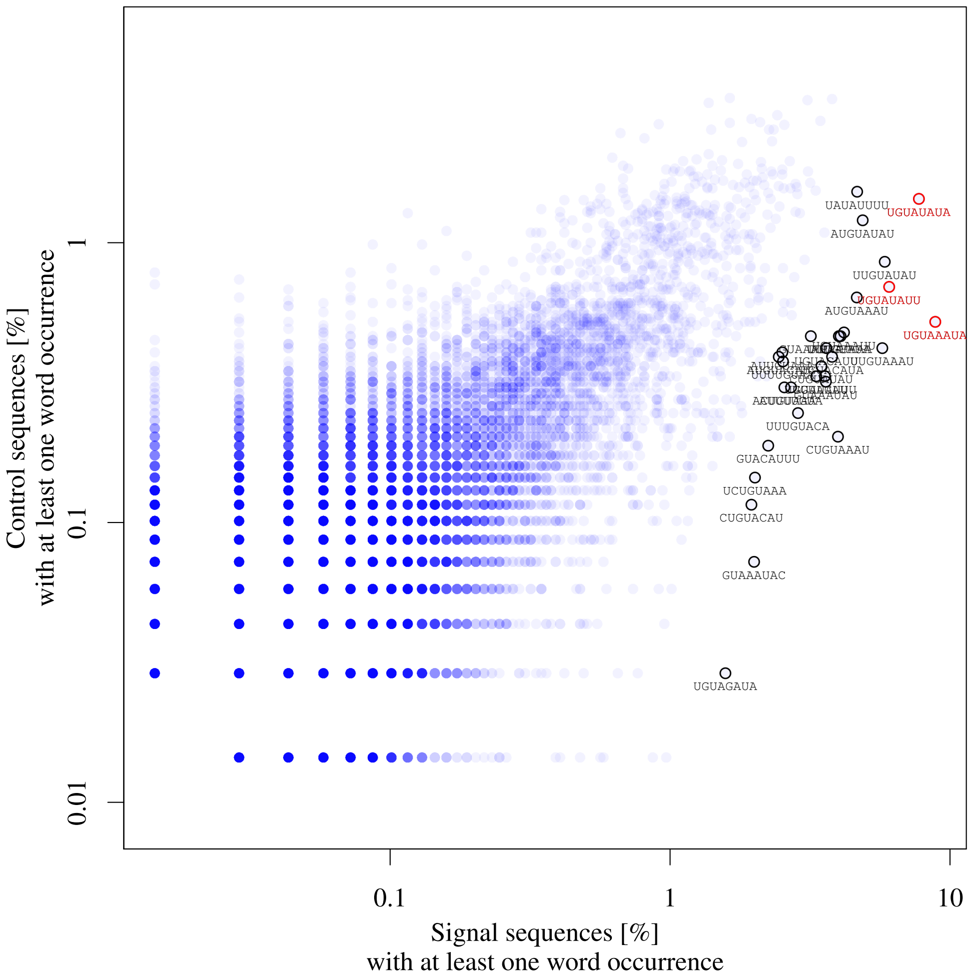

Supplementary figure S24. Scatter plot of percentages of PUM2 (34) signal and control sequences that have a given 8mer. The 30 words with highest marginal MICO are labeled. The top three words according to residual MICO are highlighted in red.

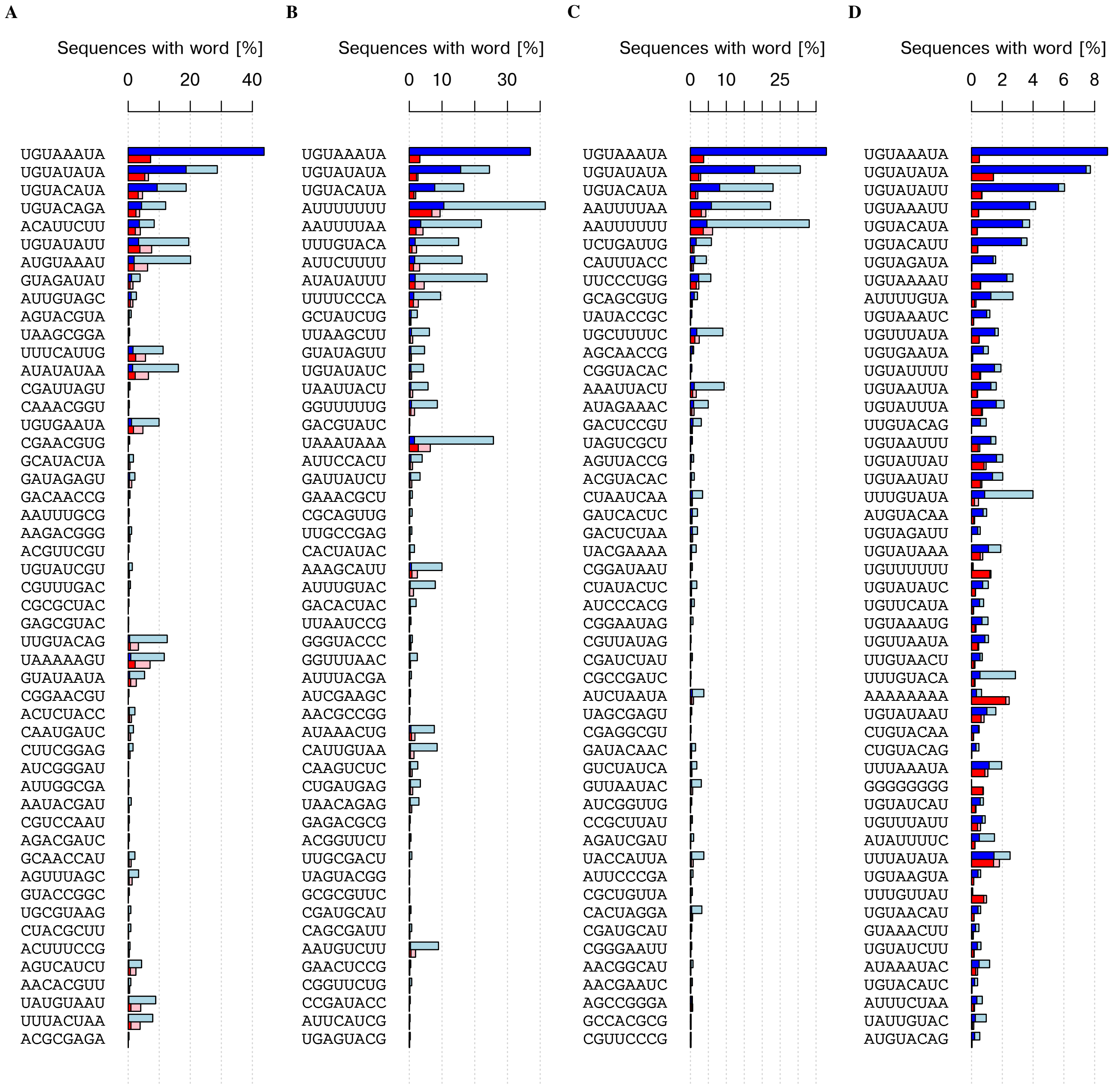

Supplementary figure S25. Discriminative word analysis of human RBP datasets of PUM1 (32) **(A)** and (33) **(B)**, and PUM2 (33) **(C)**, and (34) **(D)**. Top 50 words of length 8 according to residual MICO as determined by algorithm 1. Bars indicate how many of the sequences in signal (blue) and control (red) have at least one occurrence of the word. Light parts of the bars indicate which of the sequences have occurrences of any words with higher MICO, and thus are potentially explained by them. It it thus the dark portions of the bars that indicate the novel explanatory contribution of a word when accepting words in decreasing order of MICO.

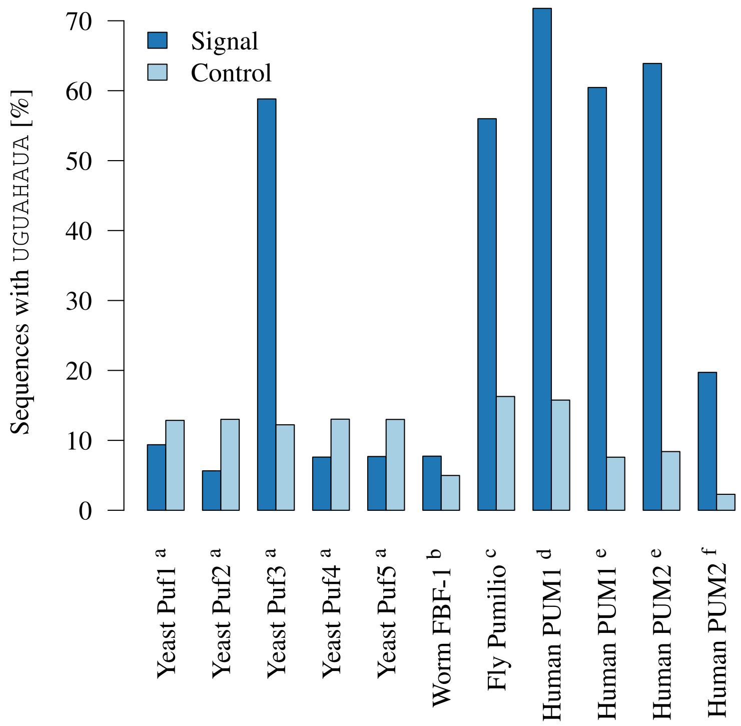

Supplementary figure S26. Number of sequences with at least one occurrence of the IUPAC motif `UGUAHAUA` in the PUF RBP family data. Data sources: [a] (29), [b] (30), [c] (31), [d] (32), [e] (33), [f] (34).

ADDITIONAL RESULTS FOR RBM10 PAR-CLIP DATA

Supplementary table T7. MICO motifs in RBM10 PAR-CLIP data from discriminative analysis versus shuffles. N_1 and N_2: number of signal sequences in dataset 1 and 2, respectively. IC: information content. S and C: expected relative frequency of signal and control sequences with at least one motif occurrence. MICO: mutual information of condition and motif occurrence. log-p: MICO based log-p value, corrected for motif length.

					**Dataset 1**				**Dataset 2**			
**Sequences**	$\mathbf{N_1}$	$\mathbf{N_2}$	**Motif**	**IC** [bit]	**S** [%]	**C** [%]	**MICO** [bit]	**log-p**	**S** [%]	**C** [%]	**MICO** [bit]	**log-p**
Exonic	7469	22 836	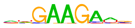	10.4	25.7	11.2	385.8	−225.8	22.2	10.7	816.2	−524.5
			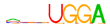	8.6	21.7	11.8	190.9	−100.3	18.2	10.9	359.8	−217.7
			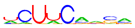	9.6	3.4	1.9	23.7	0.0	4.0	2.1	103.6	−29.5
Intronic	5908	21 764	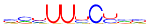	10.2	12.5	6.9	76.8	−5.8	12.6	6.9	289.3	−153.7
			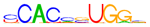	13.3	5.6	1.3	128.5	−41.9	3.5	1.0	222.7	−107.4

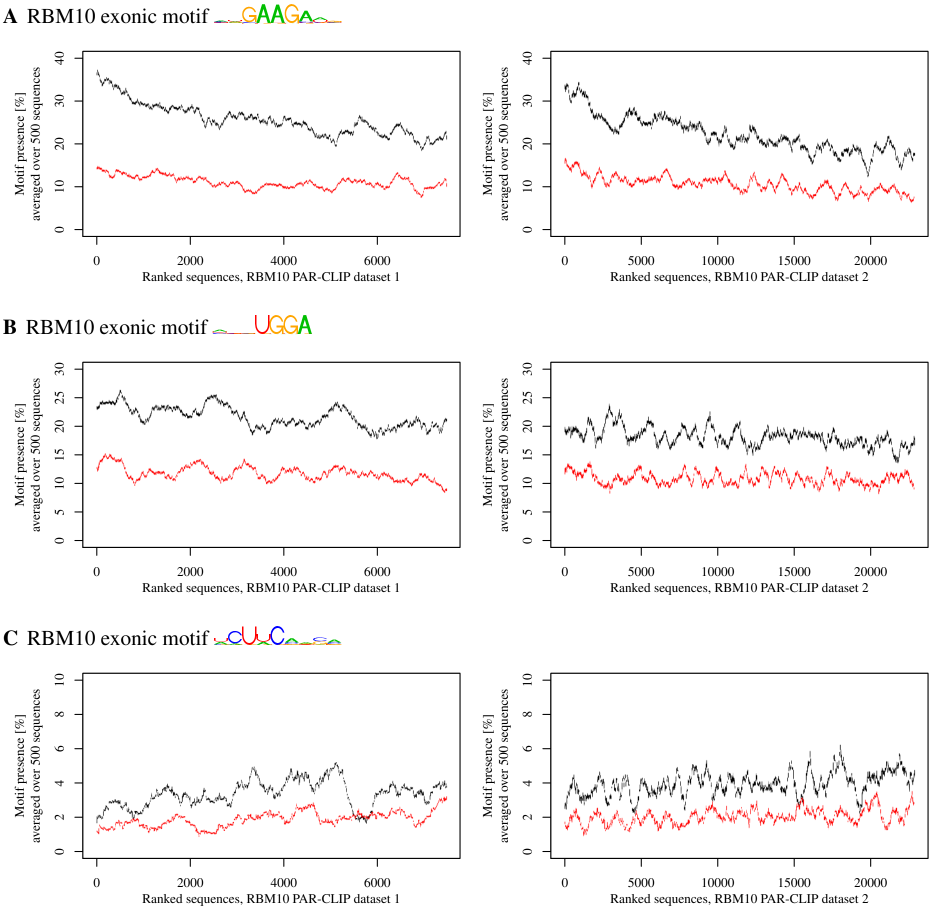

Supplementary figure S27. Occurrences of the exonic RBM10 motifs across the ranked exonic RBM10 sequences. Sequences are ranked by the number of PAR-CLIP conversions.

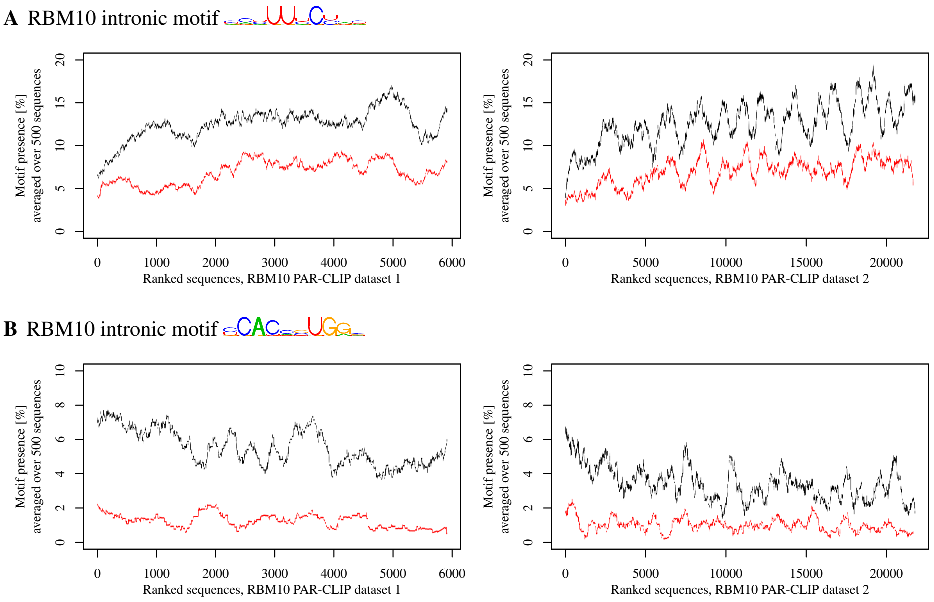

Supplementary figure S28. Occurrences of the intronic RBM10 motifs across the ranked intronic RBM10 sequences. Sequences are ranked by the number of PAR-CLIP conversions.

Supplementary table T8: RBM10 motifs of (35) in exonic RBM10 PAR-CLIP sequences of (36). (35) performed CLIP-Seq to identify RNA-binding sites of RBM10 and defined 9 groups of 5mers as RBM10 target motifs. This table gives the number of exonic sequences in the two PAR-CLIP datasets of (36) that have occurrences of these 5mers. S and C: relative frequency in percent of signal and control sequences with at least one motif occurrence. MICO: mutual information of condition and motif occurrence. log-*p*: MICO-based log-*p* value, corrected for motif length. The bars visualize the log-*p* values; black and red bars respectively correspond to enrichment in the signal or control sequences.

		PAR-CLIP dataset 1				PAR-CLIP dataset 2			
Group	Motif	S [%]	C [%]	MICO [bit]	log-*p*	S [%]	C [%]	MICO [bit]	log-*p*
1	AACUC	3.5	3.7	0.3	0.0	3.7	4.0	2.0	0.0
1	AAGUC	3.5	3.8	0.5	0.0	3.8	3.9	0.2	0.0
1	UACUC	1.9	1.7	0.3	0.0	2.2	1.6	17.9	−7.3
1	AACUG	5.6	5.9	0.4	0.0	5.4	5.9	3.1	0.0
1	UACUG	3.0	2.5	2.2	0.0	3.1	2.5	9.5	−1.2
1	GACUU	5.3	4.4	5.2	0.0	5.0	4.3	8.9	−0.8
1	GACUC	3.4	3.7	0.5	0.0	3.7	3.9	1.2	0.0
1	GACUG	4.8	6.5	14.8	−5.1	4.7	6.3	39.9	−23.0
1	UUCUC	3.4	2.9	1.5	0.0	4.0	3.2	15.5	−5.6
2	ACUCU	3.3	3.4	0.1	0.0	3.4	3.8	5.2	0.0
2	UCUGA	5.2	6.6	9.6	−1.3	5.1	6.6	37.4	−21.2
2	UCUGG	6.4	4.5	17.6	−7.1	5.6	4.4	24.3	−11.9
2	CCUGA	5.6	5.5	0.0	0.0	5.4	6.4	16.4	−6.3
2	ACUCC	2.8	2.7	0.0	0.0	3.2	3.0	1.3	0.0
2	GCUUG	2.8	4.7	27.5	−14.2	2.5	4.1	72.8	−46.0
2	ACUGA	5.4	8.0	29.0	−15.3	5.3	7.3	56.4	−34.6
2	ACUUC	4.4	3.3	8.7	−0.6	5.1	3.2	76.2	−48.4
2	UCUUG	3.0	4.4	13.8	−4.4	3.1	4.4	37.6	−21.4
2	ACUGG	6.3	5.2	6.1	0.0	5.5	5.3	0.6	0.0
2	ACUCA	3.4	4.6	10.1	−1.7	3.9	4.5	8.0	−0.1
2	UCUUC	4.3	3.1	12.0	−3.1	4.6	3.3	39.7	−22.8
2	ACUCG	1.2	1.5	1.9	0.0	1.1	1.3	4.8	0.0
2	ACUGU	2.9	2.9	0.0	0.0	3.3	3.1	1.2	0.0
2	UCUUA	2.4	2.3	0.0	0.0	2.3	2.6	2.1	0.0
2	ACUUG	3.9	4.6	3.8	0.0	3.9	4.7	10.5	−1.9

Supplementary table T8: continued from previous page.

		PAR-CLIP dataset 1				PAR-CLIP dataset 2			
Group	Motif	S [%]	C [%]	MICO [bit]	log-*p*	S [%]	C [%]	MICO [bit]	log-*p*
2	UCUGU	2.8	2.4	2.1	0.0	2.7	3.0	2.7	0.0
2	UGUGA	4.6	6.3	16.6	−6.4	4.9	6.3	34.4	−19.1
3	CUCUG	4.7	4.3	0.8	0.0	4.7	5.0	1.7	0.0
3	UUCUG	5.3	4.1	9.1	−0.9	5.1	4.2	15.3	−5.4
3	GUGUU	2.0	1.8	0.7	0.0	2.3	1.9	6.1	0.0
3	GUCUU	2.2	2.3	0.1	0.0	2.3	2.4	0.8	0.0
3	CUCUC	2.6	2.8	0.5	0.0	3.0	3.4	5.6	0.0
3	CUGUG	4.7	4.6	0.1	0.0	5.1	4.5	6.1	0.0
3	CUUUG	4.7	3.9	3.5	0.0	4.9	4.4	3.9	0.0
3	CUCUU	3.7	3.1	2.5	0.0	3.5	3.6	0.8	0.0
3	CUGUC	1.9	2.4	3.1	0.0	2.5	2.6	0.4	0.0
3	CUGUU	2.8	2.7	0.1	0.0	2.9	3.0	0.1	0.0
3	CUUUC	3.1	3.3	0.3	0.0	3.6	3.2	4.7	0.0
3	GUUUG	2.9	3.3	1.5	0.0	3.1	3.1	0.0	0.0
3	GUCUG	2.6	3.1	2.4	0.0	2.8	3.4	9.0	−0.8
4	CUGAA	10.0	8.7	5.4	0.0	9.3	8.4	7.3	0.0
4	UUGUG	3.4	4.2	4.4	0.0	3.3	4.0	11.6	−2.8
4	UUGAC	2.8	4.5	21.9	−10.2	3.0	4.5	53.2	−32.3
4	CUGAG	4.8	8.4	54.3	−33.1	4.9	7.9	120.0	−79.1
4	UUGUC	1.8	2.4	5.0	0.0	2.0	2.5	11.2	−2.5
4	CUGAC	3.0	4.2	11.5	−2.7	3.3	4.7	45.5	−27.0
4	UUGGA	11.1	7.4	45.7	−27.0	9.2	7.0	56.1	−34.4
4	UUGGG	3.8	4.5	2.9	0.0	3.5	4.2	10.6	−2.0
4	UUGAA	8.3	10.9	21.9	−10.2	8.1	9.4	18.2	−7.6
4	CUGGA	12.5	7.1	89.5	−57.8	11.1	6.9	185.1	−124.4
4	UUGUA	1.7	2.0	1.1	0.0	1.9	2.0	0.7	0.0
4	CUUGA	4.0	7.2	50.4	−30.4	4.0	6.5	100.1	−65.2
4	GUGGA	11.6	6.1	102.4	−66.8	8.8	5.2	163.2	−109.2
5	GAACU	6.8	6.3	1.0	0.0	6.0	5.6	3.1	0.0
5	GAAGG	9.7	9.4	0.2	0.0	8.8	8.1	6.0	0.0
5	GUACU	1.5	1.3	0.8	0.0	1.6	1.3	4.8	0.0
5	GAAGA	32.6	15.5	440.0	−301.6	27.5	14.4	868.2	−598.7
5	CAACU	3.6	4.0	1.7	0.0	4.0	3.9	0.0	0.0
5	GAGCU	5.7	5.1	1.6	0.0	5.0	5.3	1.2	0.0
5	GGACU	5.2	4.7	1.7	0.0	4.8	4.4	4.0	0.0
5	GAAGU	8.3	5.9	23.3	−11.2	8.0	5.6	73.7	−46.7
6	UGGAA	14.6	9.3	72.6	−45.9	13.3	8.8	167.7	−112.3
6	UGUUG	3.5	3.8	0.5	0.0	3.5	3.7	1.1	0.0
6	UGUGC	2.4	3.2	6.0	0.0	2.6	2.9	2.4	0.0
6	UGUAG	2.3	2.0	1.4	0.0	2.0	2.3	2.4	0.0
6	UGGAG	13.5	8.5	68.6	−43.1	11.8	8.0	133.2	−88.3
6	UGUAC	2.0	1.3	8.1	−0.2	1.9	1.3	18.4	−7.7
6	UGAAG	20.2	11.5	153.9	−102.7	18.0	10.4	395.8	−270.8
6	UGUCC	2.2	2.2	0.0	0.0	2.6	2.3	3.2	0.0
6	UGAAC	5.1	6.3	6.8	0.0	5.0	5.6	5.9	0.0
6	UGUUC	2.4	2.0	1.6	0.0	2.7	2.4	2.7	0.0
6	UGGAC	9.7	5.2	77.1	−49.1	7.2	4.6	97.9	−63.6
6	UGUGG	6.7	4.8	17.0	−6.7	6.1	4.5	45.8	−27.1
6	AGAAC	9.2	7.5	10.3	−1.8	8.1	7.3	9.2	−1.0
7	CUUUU	3.7	3.7	0.0	0.0	4.0	3.8	0.6	0.0
7	GAUCU	4.3	4.6	0.6	0.0	3.8	4.7	15.5	−5.6
7	UGUCU	2.4	2.9	3.2	0.0	2.8	2.9	0.7	0.0
7	CCUUU	3.1	2.9	0.4	0.0	3.6	3.2	3.7	0.0
7	GGUCU	1.7	2.3	4.8	0.0	1.8	2.4	15.0	−5.3

Supplementary table T8: continued from previous page.

Group	Motif	PAR-CLIP dataset 1 S [%]	C [%]	MICO [bit]	log-*p*	PAR-CLIP dataset 2 S [%]	C [%]	MICO [bit]	log-*p*
7	CUUCU	4.0	3.3	4.5	0.0	4.2	3.7	7.2	0.0
7	UCUCU	3.1	3.1	0.0	0.0	3.3	3.6	1.8	0.0
7	CUUGU	1.9	2.6	5.0	0.0	2.1	2.8	15.5	−5.6
7	CUUAU	2.7	1.7	11.3	−2.5	2.5	2.1	5.1	0.0
7	UCUUU	3.2	3.5	0.4	0.0	3.7	3.8	0.4	0.0
7	GCUCU	3.4	2.9	2.6	0.0	3.3	3.3	0.1	0.0
7	GCUUU	3.2	2.8	1.3	0.0	3.5	3.0	5.8	0.0
7	CCUCU	3.2	3.0	0.3	0.0	3.4	3.6	0.8	0.0
7	AGUCU	2.8	3.3	3.0	0.0	2.7	3.5	17.9	−7.3
7	UGACU	3.8	5.3	13.6	−4.2	4.2	5.1	15.2	−5.4
8	UUCCU	4.0	3.0	9.4	−1.1	4.4	3.4	24.6	−12.1
8	UGCUU	2.6	3.6	9.2	−1.0	3.1	3.5	5.4	0.0
8	UCCUU	3.3	2.6	4.0	0.0	3.5	3.2	2.4	0.0
8	UCCCU	2.3	2.6	0.8	0.0	2.9	3.0	0.4	0.0
8	UUCUU	3.3	3.5	0.6	0.0	4.1	3.8	1.5	0.0

Supplementary table T9: RBM10 motifs of (35) in intronic RBM10 PAR-CLIP sequences of (36). (35) performed CLIP-Seq to identify RNA-binding sites of RBM10 and defined 9 groups of 5mers as RBM10 target motifs. This table gives the number of intronic sequences in the two PAR-CLIP datasets of (36) that have occurrences of these 5mers. S and C: relative frequency of signal and control sequences with at least one motif occurrence. MICO: mutual information of condition and motif occurrence. log-*p*: MICO-based log-*p* value, corrected for motif length. The bars visualize the log-*p* values; black and red bars respectively correspond to enrichment in the signal or control sequences.

Group	Motif	PAR-CLIP dataset 1 S [%]	C [%]	MICO [bit]	log-*p*	PAR-CLIP dataset 2 S [%]	C [%]	MICO [bit]	log-*p*
1	AACUC	3.8	3.7	0.2	0.0	3.7	3.7	0.1	0.0
1	AAGUC	1.8	1.8	0.0	0.0	2.3	2.6	3.7	0.0
1	UACUC	3.3	3.4	0.1	0.0	3.6	2.8	17.1	−6.8
1	AACUG	3.0	3.7	3.0	0.0	3.3	4.3	20.7	−9.3
1	UACUG	2.5	3.0	2.3	0.0	2.9	3.0	0.0	0.0
1	GACUU	3.4	3.7	0.3	0.0	3.7	3.9	1.0	0.0
1	GACUC	3.6	4.5	3.8	0.0	3.4	4.1	12.8	−3.7
1	GACUG	4.6	6.3	11.2	−2.5	4.4	5.1	8.2	−0.3
1	UUCUC	10.2	7.7	17.1	−6.8	10.7	7.7	85.5	−55.0
2	ACUCU	6.3	6.9	1.3	0.0	5.9	6.2	1.8	0.0
2	UCUGA	5.2	6.3	4.8	0.0	5.3	6.1	9.7	−1.3
2	UCUGG	10.8	10.8	0.0	0.0	8.7	9.0	0.4	0.0
2	CCUGA	6.4	7.4	3.1	0.0	6.2	6.2	0.0	0.0
2	ACUCC	5.3	5.7	0.9	0.0	4.6	5.1	4.6	0.0
2	GCUUG	4.8	5.8	4.2	0.0	3.7	5.2	43.3	−25.4
2	ACUGA	3.5	3.9	1.3	0.0	3.7	4.5	13.0	−3.8
2	ACUUC	4.6	4.5	0.0	0.0	4.9	4.7	1.0	0.0
2	UCUUG	5.2	7.2	15.1	−5.3	5.1	7.3	63.6	−39.6
2	ACUGG	6.8	7.5	1.7	0.0	5.5	6.4	11.0	−2.3
2	ACUCA	3.9	4.9	4.2	0.0	4.3	4.7	3.0	0.0
2	UCUUC	8.5	7.8	1.3	0.0	8.9	7.4	20.9	−9.5
2	ACUCG	1.0	1.0	0.1	0.0	0.9	1.0	0.1	0.0
2	ACUGU	5.0	4.4	1.6	0.0	5.1	4.5	6.6	0.0
2	UCUUA	3.6	3.9	0.8	0.0	4.4	4.7	1.8	0.0
2	ACUUG	3.5	4.6	7.0	0.0	3.7	4.8	21.5	−9.9
2	UCUGU	7.3	6.3	3.1	0.0	7.0	6.2	8.0	−0.1
2	UGUGA	4.4	4.4	0.0	0.0	5.1	4.4	9.0	−0.8

Supplementary table T9: continued from previous page.

Group	Motif	PAR-CLIP dataset 1 S [%]	C [%]	MICO [bit]	log-*p*	PAR-CLIP dataset 2 S [%]	C [%]	MICO [bit]	log-*p*
3	CUCUG	12.3	12.1	0.2	0.0	10.7	10.4	0.6	0.0
3	UUCUG	8.5	8.1	0.5	0.0	8.0	7.5	3.8	0.0
3	GUGUU	3.2	3.1	0.1	0.0	3.7	3.1	7.9	–0.0
3	GUCUU	3.6	4.6	6.0	0.0	4.0	4.8	11.8	–2.9
3	CUCUC	10.0	10.1	0.0	0.0	8.8	8.7	0.1	0.0
3	CUGUG	12.6	9.8	16.8	–6.5	11.1	8.2	74.0	–46.9
3	CUUUG	5.4	5.9	1.2	0.0	5.5	6.5	14.7	–5.0
3	CUCUU	10.3	10.1	0.1	0.0	9.7	9.3	1.9	0.0
3	CUGUC	7.0	6.7	0.3	0.0	6.5	6.3	0.3	0.0
3	CUGUU	5.5	5.5	0.0	0.0	5.7	5.6	0.2	0.0
3	CUUUC	9.2	7.3	9.4	–1.2	9.6	7.2	58.6	–36.1
3	GUUUG	2.8	2.9	0.1	0.0	3.4	3.6	0.7	0.0
3	GUCUG	6.1	6.7	1.3	0.0	5.1	6.0	11.6	–2.8
4	CUGAA	4.5	4.1	0.7	0.0	4.8	4.6	0.6	0.0
4	UUGUG	4.0	4.6	1.4	0.0	4.1	5.0	14.2	–4.7
4	UUGAC	1.7	3.4	27.5	–14.2	2.5	3.8	43.9	–25.8
4	CUGAG	8.1	8.5	0.4	0.0	7.4	7.5	0.2	0.0
4	UUGUC	3.3	4.1	3.8	0.0	3.3	4.5	29.8	–15.9
4	CUGAC	4.5	6.1	10.3	–1.8	4.5	5.1	6.1	0.0
4	UUGGA	4.9	4.7	0.2	0.0	4.6	5.0	2.3	0.0
4	UUGGG	7.7	7.4	0.2	0.0	6.3	6.5	0.7	0.0
4	UUGAA	2.4	3.2	5.0	0.0	3.6	4.6	19.0	–8.1
4	CUGGA	10.1	7.9	12.8	–3.7	8.3	6.8	25.1	–12.5
4	UUGUA	1.4	1.8	1.9	0.0	2.0	2.7	13.7	–4.3
4	CUUGA	3.1	4.9	18.3	–7.6	3.5	5.5	70.5	–44.5
4	GUGGA	6.0	4.6	8.4	–0.4	5.4	4.4	16.9	–6.6
5	GAACU	3.1	2.9	0.2	0.0	2.8	3.2	5.7	0.0
5	GAAGG	4.7	3.8	3.6	0.0	4.7	4.2	4.2	0.0
5	GUACU	1.8	2.0	0.9	0.0	1.7	2.0	3.0	0.0
5	GAAGA	4.9	2.9	22.5	–10.7	5.7	3.7	68.9	–43.4
5	CAACU	2.4	4.5	27.8	–14.4	2.9	4.3	42.9	–25.1
5	GAGCU	5.0	5.4	0.8	0.0	4.8	5.0	0.6	0.0
5	GGACU	5.0	5.1	0.1	0.0	4.2	4.9	7.6	0.0
5	GAAGU	2.8	2.1	4.2	0.0	3.1	2.6	5.8	0.0
6	UGGAA	5.6	3.6	20.5	–9.2	5.9	4.3	38.8	–22.2
6	UGUUG	4.0	4.3	0.7	0.0	3.8	4.6	12.2	–3.2
6	UGUGC	6.3	5.9	0.8	0.0	5.6	5.5	0.2	0.0
6	UGUAG	2.1	1.9	0.4	0.0	2.1	2.3	0.7	0.0
6	UGGAG	8.9	7.5	5.5	0.0	8.2	6.9	17.7	–7.2
6	UGUAC	1.6	2.0	1.6	0.0	2.0	2.2	0.9	0.0
6	UGAAG	4.9	3.6	8.5	–0.5	5.2	4.4	12.2	–3.2
6	UGUCC	6.1	5.5	1.5	0.0	5.6	5.2	3.2	0.0
6	UGAAC	2.5	3.1	2.7	0.0	2.6	3.1	6.1	0.0
6	UGUUC	4.2	3.9	0.5	0.0	4.5	4.6	0.3	0.0
6	UGGAC	6.3	5.1	5.3	0.0	4.7	4.8	0.1	0.0
6	UGUGG	10.6	8.8	8.4	–0.5	8.7	7.2	24.4	–12.0
6	AGAAC	2.7	1.9	5.3	0.0	2.7	2.6	0.1	0.0
7	CUUUU	7.6	7.4	0.1	0.0	9.2	8.4	5.8	0.0
7	GAUCU	2.2	3.4	9.8	–1.5	2.5	3.6	33.7	–18.6
7	UGUCU	5.9	6.1	0.2	0.0	5.8	6.2	1.9	0.0
7	CCUUU	8.1	7.3	2.1	0.0	8.5	7.0	22.4	–10.6
7	GGUCU	5.3	6.3	3.9	0.0	4.6	5.6	15.6	–5.7
7	CUUCU	9.9	10.1	0.0	0.0	9.7	8.8	7.7	0.0
7	UCUCU	10.3	9.7	1.0	0.0	10.4	9.1	17.2	–6.8

Supplementary table T9: continued from previous page.

Group	Motif	PAR-CLIP dataset 1 S [%]	C [%]	MICO [bit]	log-p	PAR-CLIP dataset 2 S [%]	C [%]	MICO [bit]	log-p
7	CUUGU	3.8	5.4	12.3	−3.3	3.9	5.6	51.7	−31.3
7	CUUAU	2.8	3.1	0.7	0.0	3.3	3.7	3.7	0.0
7	UCUUU	8.4	7.8	1.1	0.0	9.6	9.0	3.7	0.0
7	GCUCU	7.9	8.1	0.0	0.0	6.9	7.1	0.7	0.0
7	GCUUU	5.0	4.4	1.9	0.0	5.1	4.6	4.3	0.0
7	CCUCU	12.2	11.5	0.8	0.0	10.0	10.0	0.0	0.0
7	AGUCU	2.8	3.9	7.0	0.0	3.1	4.5	43.6	−25.6
7	UGACU	4.3	5.6	8.3	−0.4	4.6	5.2	5.9	0.0
8	UUCCU	12.4	7.7	51.1	−30.9	11.6	7.8	130.9	−86.6
8	UGCUU	5.1	5.8	2.0	0.0	5.6	5.8	1.3	0.0
8	UCCUU	10.1	8.4	7.1	0.0	9.6	7.9	27.8	−14.4
8	UCCCU	9.5	9.0	0.7	0.0	8.5	7.9	4.1	0.0
8	UUCUU	8.2	7.9	0.3	0.0	9.5	8.5	9.8	−1.5

Supplementary table T10. Summary statistics for enrichment in the PAR-CLIP data of (36) of the 94 CLIP-Seq-based RBM10 motifs reported by (35). See supplementary tables T8 and T9 for details. Absolute and relative numbers of RBM10 motifs that are less or equally frequent ($\leq$), more frequent ($>$), or much more frequent ($\gg$) in the signal sequences compared to shuffled sequences. Motifs are counted as much more frequent if their relative frequency is higher in signal than control and the MICO-based log-p value is less than or equal to −10.

Data set	$S \leq C$	[%]	$S > C$	[%]	$S \gg C$	[%]
Exonic 1	45	47.9	49	52.1	9	9.6
Exonic 2	49	52.1	45	47.9	14	14.9
Intronic 1	49	52.1	45	47.9	2	2.1
Intronic 2	54	57.4	40	42.6	10	10.6

Supplementary table T11. RBM10 motifs of (37) in **(A)** exonic and **(B)** intronic RBM10 PAR-CLIP sequences of (36). (37) defined motifs based on the sequences of 5' splice sites of two exons affected by RBM10 knock-down. This table gives the number of exonic sequences in the two PAR-CLIP datasets of (36) that have occurrences of these motifs. The vertical bar in the motif indicates exon-intron boundary of the two example sequences (note: all occurrences in PAR-CLIP sequences are counted, whether across exon-intron boundaries or not). S and C: relative frequency in percent of signal and control sequences with at least one motif occurrence. MICO: mutual information of condition and motif occurrence. log-p: MICO-based log-p value, corrected for motif length. The bars visualize the log-p values; black and red bars respectively correspond to enrichment in the signal or control sequences.

A Exonic PAR-CLIP sequences

Motif	PAR-CLIP dataset 1 S [%]	C [%]	MICO [bit]	log-p	PAR-CLIP dataset 2 S [%]	C [%]	MICO [bit]	log-p
AG | GUAA	0.6	0.8	2.3	0.0	0.5	0.7	4.0	0.0
GG | GUAAG	0.1	0.1	0.6	0.0	0.1	0.1	3.5	0.0

B Intronic PAR-CLIP sequences

Motif	PAR-CLIP dataset 1 S [%]	C [%]	MICO [bit]	log-p	PAR-CLIP dataset 2 S [%]	C [%]	MICO [bit]	log-p
AG | GUAA	0.5	0.3	4.4	0.0	0.6	0.4	2.3	0.0
GG | GUAAG	0.2	0.1	0.0	0.0	0.1	0.1	0.1	0.0

SUPPLEMENTARY CHIP-SEQ RESULTS

Discriminative motif analysis versus shuffles

We compared mouse ESC TF ChIP-Seq data sets of (38) and (39) individually against shuffles, running Discrover in multiple motif discovery mode. Supplementary table T12 gives detailed results. The table gives the ChIP'd protein and the number of signal sequences (in each case as many shuffled sequences as signal sequences were used as control). For each ChIP'd protein one or motifs were discovered, and for each motif we list the information content, the expected number of occurrences in signal and control, the MICO value, and the MICO-based log p-value. Also, where the discovered motifs have previously been described, we list the factor known to bind the pattern, highlighting those factors that are among the ChIP'd proteins considered here. In case multiple members of a protein family are known to bind to a pattern we list only the family name, as we do e.g. for the Myc pattern.

Note that the number of reported motif occurrences in supplementary table T12 is calculated for the resulting multi-motif model. A consequence of this is that, where multiple motifs are discovered, the counts of occurrence of individual motifs compete for positions in sequences, and—compared to corresponding single motif models—the number of occurrences are underestimated. This is for example the case in data sets for which both Sox2 monomer and Sox2-Oct4 heterodimer patterns are discovered. MICO, as well as the MICO-based log p-value, are calculated from the listed counts.

Analysis of motif occurrence localization

We determined occurrences of the discovered motifs in larger, 501 nt regions around the sequence mid-points. The distributions of occurrences of discovered motifs in signal and control sequences are shown in supplementary table T13. Note that the number of occurrences in supplementary table T12 are expected counts (and are based on 101 nt windows), while the positional distribution plots of supplementary table T13 are based on Viterbi decoded motif occurrences.

Where—aside from other motifs—Discrover finds the previously described cognate motifs, the cognate motifs' occurrences are visibly more concentrated around the sequence midpoints than those of the other motifs.

Contrasting Nanog and Tcf3 sequences against datasets enriched for Sox2-Oct4 heterodimer binding pattern

We analyzed the Nanog and Tcf3 ChIP-Seq datasets of (38) and (39) by individually contrasting their sequence sets against those of Oct4, Sox2, and Tcf3, and those of Nanog, Oct4, and Sox2, respectively. For each contrast the three most discriminative motifs of lengths 5–16 nt were identified by heuristically maximizing MICO over the space of IUPAC regular expressions using Plasma. HMMs were seeded on these IUPAC motifs, as well as one one nucleotide-shifted variants. In total, $3 \times 12 \times 3 = 108$ HMMs were trained for each contrast. The HMMs yielding the best MICO-based log p-value and are enriched in the Nanog datasets, respectively those of Tcf3, are shown in supplementary figure T14.

For the Nanog contrasts, we found in 9/10 cases the previously described Nanog motif. In the case of the Nanog (39) versus Oct4 (38) datasets, the Sox2 motif is more highly enriched in the Nanog (39) sequences versus the Oct4 (38) sequences, than the Nanog motif. In all six Tcf3 contrasts we find the Tcf3 motif.

Comparison of motif discovery results for Oct4 with Discrover, DREME, and FIRE

We compare motif discovery results of Discrover, DREME, and FIRE for the Oct4 data of (38). In addition to the set of shuffled sequences used in the analysis presented in supplementary table T12, we generate two further sets of shuffles of the Oct4 ChIP-Seq sequences as controls for discriminative motif discovery. We apply Discrover, DREME, and FIRE to the three contrasts defined by the Oct4 sequences and the three sets of shuffles. Discrover is run in same manner as explained in the ChIP-Seq section of the methods part of the main text, and DREME and FIRE in their default way for the analysis of DNA-binding proteins. The resulting motifs are displayed in decreasing order of significance (as reported by the methods) in supplementary table T15.

As is visible, Discrover consistently identifies for the three contrasts the same two motifs of the Sox2-Oct4 heterodimer pattern and the Klf motif.

DREME discovers between 18 and 21 motifs. The top DREME motifs (according to the DREME E-value) are identically discovered in the three contrasts. The lower ranking DREME motifs are however not all re-discovered in each contrast, and their significance order is differing. The DREME results include the Klf motif, as well as multiple motifs that are consistent with parts of the Sox2-Oct4 heterodimer pattern. Yet, as DREME is designed for the discovery of short motifs, no single one of them is long enough to explain the entire Sox2-Oct4 heterodimer pattern. Overall, between 7–10 of the motifs discovered by DREME in each of the contrasts are clearly redundantly referring to different parts of this long pattern.

FIRE discovers between 12 and 15 motifs, including motifs that are depleted in the signal sequences. Of the motifs discovered by FIRE 8–10 are enriched in the signal sequences. As for the DREME results, FIRE finds in each contrast the Klf motif, and 3–6 short motifs corresponding to different parts of the Sox2-Oct4 heterodimer pattern.

DREME also consistently identifies two motifs that apparently correspond to the Esrrb and Myc patterns, which Discrover misses in the Oct4 data of (38) but discovers in other datasets analyzed here. FIRE finds the Esrrb motif in 2 of 3 analyses and does not discover the Myc pattern. The remaining motifs of low significance reported by DREME and FIRE are not ESC-related, do not clearly correspond to any known patterns, or are not consistently found in the analysis of the three contrasts.

Analysis runtime As is shown in supplementary table T12, Discrover needs longer than DREME for the analysis of the Oct4 datasets. DREME and FIRE run in 13–17 min on a single CPU, while Discrover requires slightly less than 2 h using eight CPU cores (experiments run on an Intel® Core^{TM} i7-4770K CPU @ 3.50GHz with eight cores). DREME and FIRE are designed for short motifs, and due to their algorithmic design, memory and runtime scaling prohibit (on our computers) discovery of motifs of length up to 16 nt.

DREME discovered motifs of length 5–8 nt, FIRE seeds words of length 7 nt that are then extended on both sides by 1 nt, yielding a total motif length of 9 nt. In contrast, Discrover considered the length range 5–16 nt. When Discrover only considers motifs of the length range 5–8 nt (results not shown), it requires less than 30 min. Thus, Discrover's analysis of longer motif causes most of the runtime increase.

Supplementary table T12: Discriminative motif analysis of mouse ChIP-Seq data. Protein: ChIP'd protein. N: number of signal sequences. Motifs: One or more motifs discovered in the sequences of the ChIP'd protein. Factor: TF (family) known to bind the discovered motif (TOMTOM q-value ≤ 0.05 (40)), bold if one of the ChIP'd proteins. IC: information content. S and C: expected relative frequency of signal and control sequences with at least one motif occurrence. MICO: mutual information of condition and motif occurrence. log-p: MICO based log-p value, corrected for motif length. Data sources: [a] (38), [b] (39).

Protein	N	Motifs	Factor	IC [bit]	S [%]	C [%]	MICO [bit]	log-p
c-Myc [a]	3422	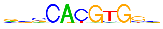	**Myc**	12.5	40.9	8.4	747.8	−467.1
Ctcf [a]	39 609	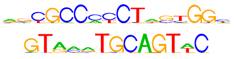	**Ctcf**	18.7	83.4	3.5	43 892.7	−30 350.6
			?	20.3	5.2	0.2	1753.4	−1154.5
E2f1 [a]	20 699	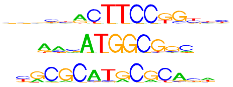	Ets TF family	14.6	5.6	1.4	414.8	−210.9
			Yy1	14.9	3.8	0.6	397.5	−223.9
			Nrf1	17.1	3.1	0.4	360.9	−183.4
Esrrb [a]	21 647	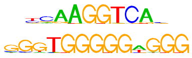	**Esrrb**	14.0	68.3	6.7	14 108.6	−9735.0
			?	21.2	3.5	0.5	406.0	−219.8
Klf4 [a]	10 875	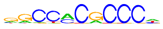	**Klf/Sp1**	14.8	57.5	8.0	4767.3	−3254.4
Nanog [a]	10 343	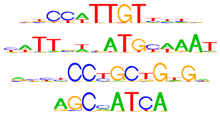	**Sox2**	13.4	29.8	6.8	1410.1	−916.5
			**Sox2-Oct4**	16.6	21.4	2.9	1341.9	−859.2
			Zic	14.8	13.1	2.2	685.0	−408.4
			**Nanog**	13.0	8.0	2.8	202.7	−103.5
Nanog [b]	16 667	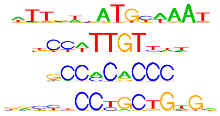	**Sox2-Oct4**	15.5	25.8	4.1	2430.6	−1619.1
			**Sox2**	13.3	24.1	6.6	1487.1	−979.9
			**Klf/Sp1**	14.0	10.1	2.2	699.1	−443.2
			Zic	15.8	6.6	1.2	519.8	−288.8
n-Myc [a]	7182	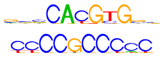	**Myc**	12.1	33.8	7.6	1152.3	−747.7
			**Klf/Sp1**	16.0	10.5	3.1	235.4	−116.3
Oct4 [a]	3761	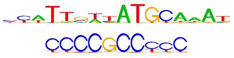	**Sox2-Oct4**	18.1	42.3	2.6	1422.7	−910.3
			**Klf/Sp1**	16.1	7.3	1.6	109.8	−28.8
Oct4 [b]	17 225	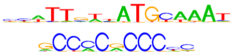	**Sox2-Oct4**	17.4	46.8	3.8	6898.7	−4706.8
			**Klf/Sp1**	15.5	8.4	1.7	635.2	−388.9
Smad1 [a]	1126	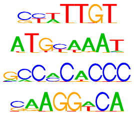	**Sox2**	11.2	31.0	9.6	119.5	−50.7
			**Oct4**	12.2	17.5	4.5	74.3	−14.1
			**Klf/Sp1**	14.2	13.2	2.5	69.7	−5.8
			**Esrrb**	13.3	10.1	1.7	56.3	−1.5
Sox2 [a]	4526	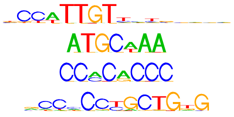	**Sox2**	13.5	64.0	10.3	2177.7	−1434.0
			**Oct4**	13.0	11.3	2.7	194.2	−102.6
			**Klf/Sp1**	14.1	4.7	1.2	74.1	−13.9
			Zic	16.3	6.2	0.9	149.2	−41.3
Sox2 [b]	15 036	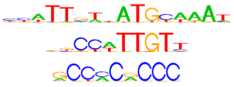	**Sox2-Oct4**	17.5	38.9	3.3	4720.2	−3196.6
			**Sox2**	13.2	30.2	6.7	2132.0	−1432.1
			**Klf/Sp1**	14.1	7.7	1.4	554.1	−342.6
Stat3 [a]	2546	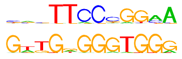	**Stat3**	14.1	40.0	3.7	800.8	−498.9
			?	18.6	6.8	0.8	98.6	−11.0
Tcf3 [b]	6257	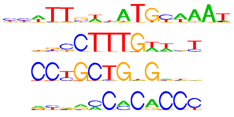	**Sox2-Oct4**	17.2	38.0	3.7	1829.5	−1192.4
			**Tcf3**	14.5	22.5	4.3	702.2	−430.5
			Zic	15.2	5.4	0.8	175.2	−64.4
			**Klf/Sp1**	14.1	5.5	1.1	150.5	−47.2
Tcfcp2l1 [a]	26 910	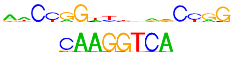	**Tcfcp2l1**	14.6	75.0	10.9	17 829.4	−12 284.1
			**Esrrb**	15.5	5.2	0.8	713.0	−457.9

Supplementary table T12: continued from previous page.

Protein	N	Motifs	Factor	IC [bit]	S [%]	C [%]	MICO [bit]	log-p
Zfx [a]	10 338	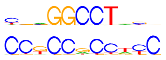	**Zfx**<br>?	11.5<br>15.0	44.4<br>6.8	17.5<br>2.4	1287.5<br>170.3	−841.4<br>−66.0

Supplementary table T13: Positional distribution of occurrences of predicted motifs of supplementary table T12 in windows of up to 250 nt from the peak of the ChIP-Seq regions. Black: occurrences in the signal sequences, red: occurrences in the control sequences. Note that windws of length 101 nt were used for motif discovery and form basis of the statistics listed in supplementary table T12. Data sources: [a] (38), [b] (39).

Protein	Motif 1	Motif 2	Motif 3	Motif 4
c-Myc [a]	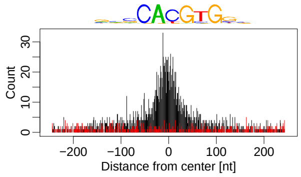			
Ctcf [a]	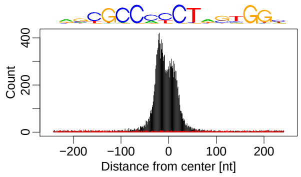	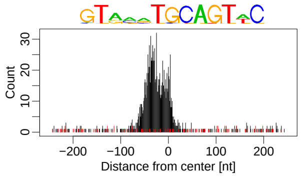		
E2f1 [a]	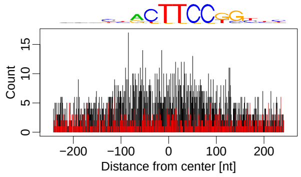	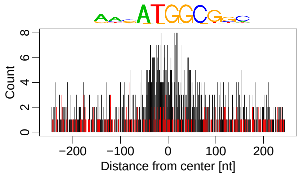	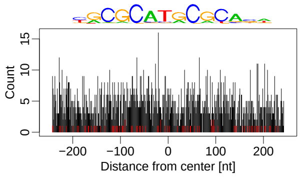	
Esrrb [a]	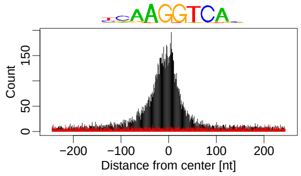	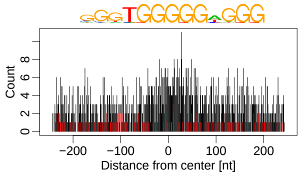		
Klf4 [a]	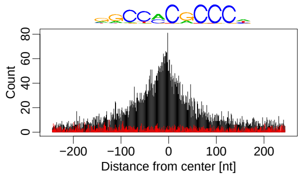			
Nanog [a]	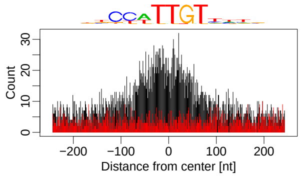	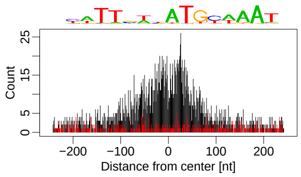	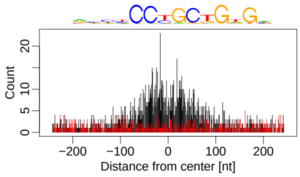	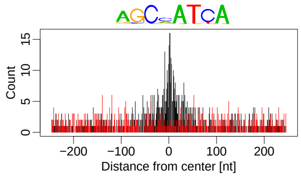
Nanog [b]	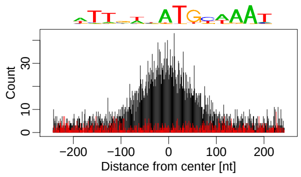	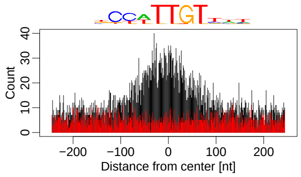	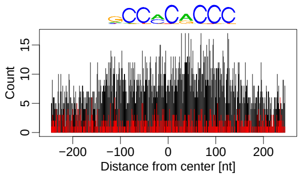	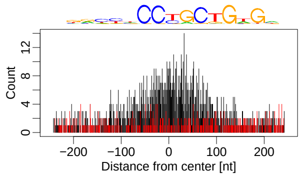

Supplementary table T13: continued from previous page.

Protein	Motif 1	Motif 2	Motif 3	Motif 4
n-Myc [a]	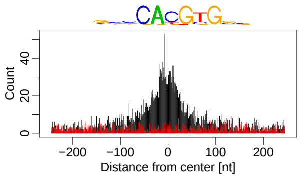	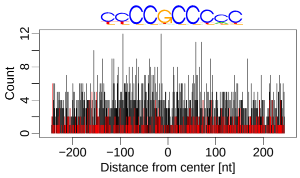		
Oct4 [a]	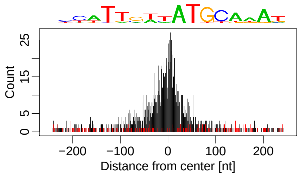	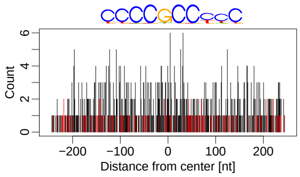		
Oct4 [b]	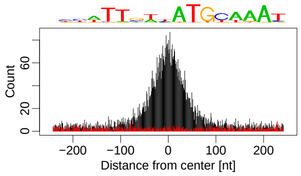	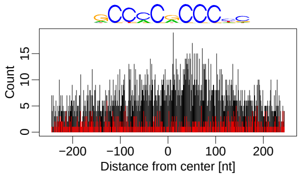		
Smad1 [a]	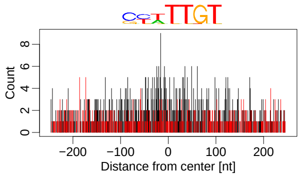	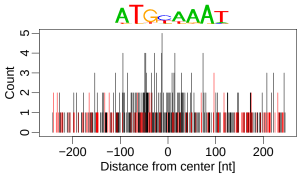	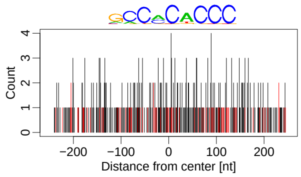	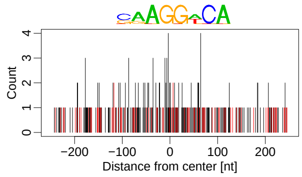
Sox2 [a]	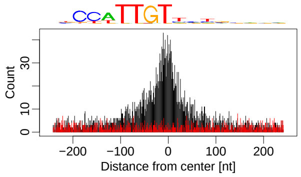	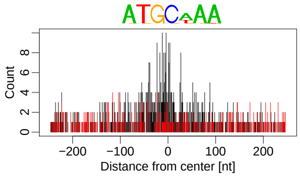	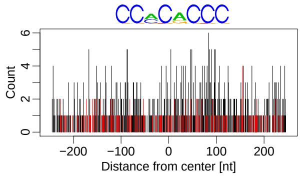	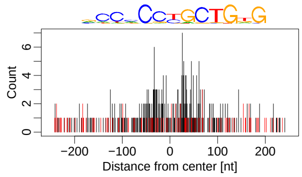
Sox2 [b]	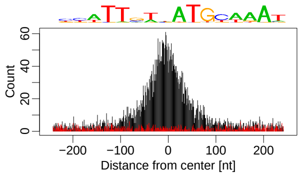	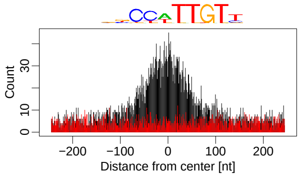	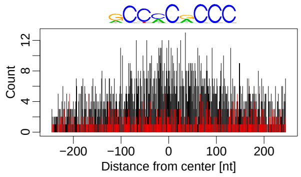	
Stat3 [a]	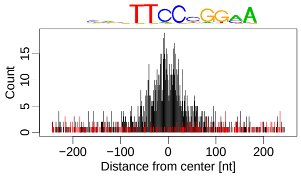	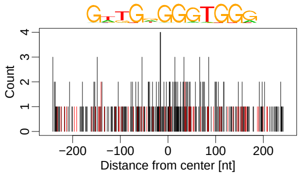		
Tcf3 [b]	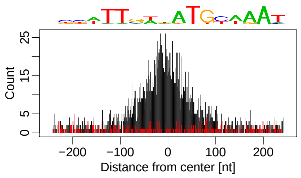	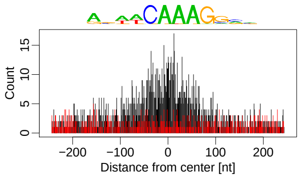	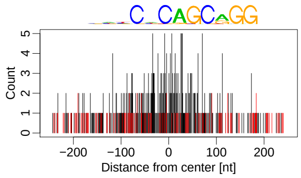	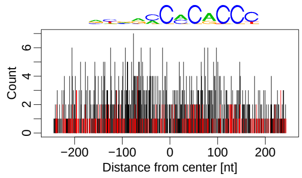
Tcfcp2l1 [a]	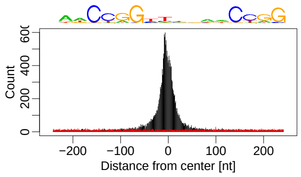	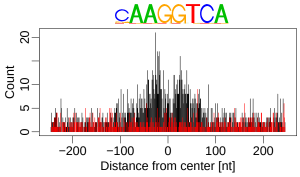		

Supplementary table T13: continued from previous page.

Protein	Motif 1	Motif 2	Motif 3	Motif 4
Zfx [a]	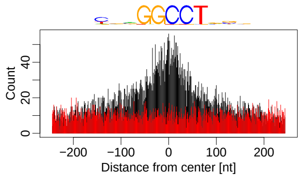	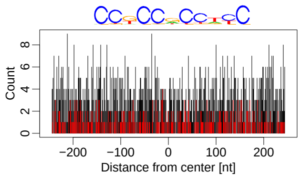		

Supplementary table T14. Inter-dataset comparison reveals motifs discriminating Nanog and Tcf3 data from other ChIP-Seq data. **(A)**,**(B)** comparing Nanog ChIP-Seq sequences against those of Oct4, Sox2, and Tcf3. (**C**): comparing Tcf3 ChIP-Seq sequences against those of Nanog, Oct4, and Sox2. Data sources: [A] (38), [B] (39).

A Nanog [A], N=10 343

vs. Protein	N	Motifs	Factor	IC [bit]	S [%]	C [%]	MICO [bit]	log-*p*
Oct4 [A]	3761	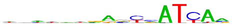	Nanog	10.1	30.8	10.7	478.7	−255.3
Oct4 [B]	17 225	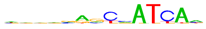	Nanog	10.8	23.4	8.5	827.4	−507.3
Sox2 [A]	4526	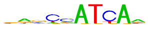	Nanog	10.1	23.9	11.0	256.7	−131.1
Sox2 [B]	15 036	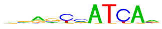	Nanog	10.5	22.6	9.7	568.5	−342.6
Tcf3 [B]	6257	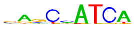	Nanog	10.5	19.9	8.6	290.0	−159.2

B Nanog [B], N=16 667

vs. Protein	N	Motifs	Factor	IC [bit]	S [%]	C [%]	MICO [bit]	log-*p*
Oct4 [A]	3761	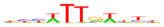	Sox2	8.3	53.0	31.9	402.9	−227.7
Oct4 [B]	17 225	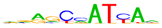	Nanog	11.0	14.0	6.5	374.7	−208.0
Sox2 [A]	4526	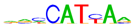	Nanog	10.9	10.3	5.1	92.4	−21.7
Sox2 [B]	15 036	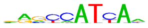	Nanog	11.3	11.1	5.7	217.5	−103.8
Tcf3 [B]	6257	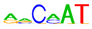	Nanog	8.5	25.4	19.8	57.8	−12.5

C Tcf3 [B], N=6257

vs. Protein	N	Motifs	Factor	IC [bit]	S [%]	C [%]	MICO [bit]	log-*p*
Nanog [A]	10 343	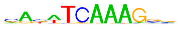	Tcf3	14.7	26.0	6.4	883.9	−556.4
Nanog [B]	16 667	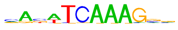	Tcf3	14.9	24.6	6.6	924.4	−584.6
Oct4 [A]	3761	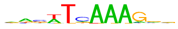	Tcf3	12.0	38.4	11.2	681.7	−416.2
Oct4 [B]	17 225	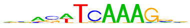	Tcf3	13.8	30.1	8.8	1078.2	−686.2
Sox2 [A]	4526	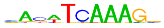	Tcf3	13.8	29.7	7.5	636.5	−389.8
Sox2 [B]	15 036	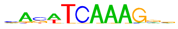	Tcf3	14.7	24.6	5.6	1042.9	−666.8

Supplementary table T15. Comparison of discriminative motif discovery with **(A)** Discrover, **(B)** DREME, and **(C)** FIRE on Oct4 data of (38) against three sets of shuffled sequences. Motifs are presented in decreasing order of significance as reported by the method. Factor: factor binding the motif; factor identification based on TOMTOM searches (with q-value < 0.05), with manual judgement in ambiguous cases, and in cases (denoted '?') where TOMTOM did not identify matches. Enrich: sample in which the FIRE motif is enriched; +: signal sequences, -: control sequences. DREME and FIRE discover IUPAC regular expression motifs, shown in these tables. Note, that for DREME and FIRE PWMs could be built from the statistics of words matching the discovered regular expressions. The final rows list wall clock and CPU time (hours:minutes:seconds).

A Discover

	Shuffles 1		Shuffles 2		Shuffles 3	
**Rank**	**Motif**	**Factor**	**Motif**	**Factor**	**Motif**	**Factor**
1	TTATGCAA	Sox2-Oct4	TTATGCAA	Sox2-Oct4	TTATGCAA	Sox2-Oct4
2	CCCCGCCC	Klf/Sp1	CCCCGCCC	Klf/Sp1	CCCCGCCC	Klf/Sp1
Wall	01:54:12		01:54:41		01:57:28	
CPU	11:38:04		11:32:09		11:30:48	

B DREME

	Shuffles 1		Shuffles 2		Shuffles 3	
**Rank**	**Motif**	**Factor**	**Motif**	**Factor**	**Motif**	**Factor**
1	ATGAA	Oct4	ATGAA	Oct4	ATGAA	Oct4
2	TTGT AT	Sox2-Oct4	TTGTAT	Sox2-Oct4	TGATA	Oct4
3	TGATA	Oct4	TGATA	Oct4	CTTGT	Sox2
4	CCC CCC	Klf/Sp1	CTTGT	Sox2	CCC CCC	Klf/Sp1
5	CTTGT	Sox2	CCC CCC	Klf/Sp1	TTCC	Ets TF family
6	TTCC	Ets TF family	CTTCC	Ets TF family?	TTGTAT	Sox2-Oct4
7	ATGCGCA	Oct4?	TGCGCA	Oct4?	ATGCGCA	Oct4?
8	CAAGGTA	Esrrb	AAGGTCA	Esrrb	CAAGGTA	Esrrb
9	ATCAGAT	Oct4?	C GGA	?	ATGAGAT	Oct4?
10	CACCC	Klf/Sp1	GG GGGA	?	AAAAG	?
11	GGGA	?	ATCAGAT	Oct4?	ATGGAAT	Oct4?
12	CTTGT	Sox2?	CACCC	Klf/Sp1	CCGCC	Sp/Egr
13	ATTAAA	Oct4?	CTTGT	Sox2	TGAAT	Sox8
14	CTCCG	?	CGGA	?	CTGG	?
15	TTGAAAT	Oct4?	TTGAAAT	Oct4?	CACGTG	Myc
16	CACGTG	Myc	GAA GGA	?	CCTGTG	Zic
17	CTG CCTC	?	ATTAAAT	Oct4?	GCAATTA	Oct4
18	CCTGGGG	?	CACGC	?	CTCCG	?
19	AGTGGCG	Yy1	CACGTG	Myc		
20			C GTTTCC	?		
21			ATGGAAT	Oct4?		
Wall	00:16:36		00:16:50		00:15:43	
CPU	00:16:36		00:16:50		00:15:43	

C FIRE

	Shuffles 1			Shuffles 2			Shuffles 3		
**Rank**	**Motif**	**Factor**	**Enrich**	**Motif**	**Factor**	**Enrich**	**Motif**	**Factor**	**Enrich**
1	AT AT	Oct4?	+	ATG A	Oct4?	+	AT AT	Oct4?	+
2	CC CC	Klf/Sp1	+	TTG	?	-	TTTGT	Sox2?	+
3	AT	?	-	C CC	Klf/Sp1	+	AT	?	-
4	TTTGT	Sox2?	+	AT CA A	Oct4?	+	C CCC	Klf/Sp1	+
5	A AA	?	-	TCA	?	-	TTCC	Ets TF family	+
6	T G CA	?	-	TTCC	Ets TF family	+	TCA	?	-
7	A G	?	-	A GCA	Nr4A2	+	TT G	?	-
8	TCC	Ets TF family?	+	TGCG A A	Oct4?	+	AT C CA	Oct4?	+
9	TGCG A	Oct4?	+	TTATT A	?	+	GATAA	?	+
10	GTTG	?	-	C C T	?	-	T GCA	?	-
11	ATATGC	Sox2-Oct4?	+	T GG	?	-	AAGGTCA	Esrrb	+
12	A A A	?	+	GCA	?	-	G A G	?	+
13	CAAGGT	Esrrb	+	TTTGTT	Sox2?	+	CACA AG	?	+
14				A TTT	?	-			
15				TGAGA T	Oct4?	+			
16				TTGTAAA	Oct4?	+			
Wall	00:13:02			00:14:54			00:14:03		
CPU	00:13:02			00:14:54			00:14:03		

SUPPLEMENTARY REFERENCES

1. Matthews,B.W. (1975) Comparison of the predicted and observed secondary structure of T4 phage lysozyme. *Biochim Biophys Acta*, **405**, 442–451.
2. Fisher,R.A. (1922) On the interpretation of χ^2 from contingency tables, and the calculation of P. *Journal of the Royal Statistical Society*, **85**, 87–94.
3. Mehta,C.R. and Patel,N.R. (1983) A Network Algorithm for Performing Fisher's Exact Test in $r \times c$ Contingency Tables. *Journal of the American Statistical Association*, **78**, pp. 427–434.
4. Pearson,K. (1900) On the criterion that a given system of deviations from the probable in the case of a correlated system of variables is such that it can be reasonably supposed to have arisen from random sampling. *Philosophical Magazine, Series 5*, **50**, 157–175.
5. Shannon,C.E. (1948) A mathematical theory of Communication. *The Bell System Technical Journal*, **27**, 379–423, 623–656.
6. Cover,T.M. and Thomas,J.A. (1991, 2006) *Elements of Information Theory*. Wiley-Interscience.
7. MacKay,D.J. (2003) *Information Theory, Inference, and Learning Algorithms*. Cambridge University Press.
8. Lindgren,B. (1993 and 1998) *Statistical Theory*. Chapman & Hall, 4 edition.
9. Sokal,R.R. and Rohlf,F.J. (1969) *Biometry: Principles and Practice of Statistics in Biological Research*. W.H.Freeman & Co Ltd.
10. Wilks,S.S. (1938) The Large-Sample Distribution of the Likelihood Ratio for Testing Composite Hypotheses. *Ann. Math. Statist.*, **9**, 60–62.
11. Koller,D. and Friedman,N. (2009) *Probabilistic Graphical Models, Principles and Techniques*. MIT Press.
12. Elemento,O., Slonim,N. and Tavazoie,S. (2007) A universal framework for regulatory element discovery across all genomes and data types. *Mol Cell*, **28**, 337–350.
13. Simpson,E.H. (1951) The Interpretation of Interaction in Contingency Tables. *Journal of the Royal Statistical Society. Series B (Methodological)*, **13**, 238–241.
14. Rabiner,L.R. (1989) A tutorial on hidden Markov models and selected applications in speech recognition. *Proceedings of the IEEE*, **77**, 257–286.
15. Rabiner,L. and Juang,B.H. (1993) *Fundamentals of Speech Recognition*. Prentice-Hall.
16. Durbin,R., Eddy,S.R., Krogh,A. and Mitchison,G. (1998) *Biological Sequence Analysis: Probabilistic Models of Proteins and Nucleic Acids*. Cambridge University Press.
17. Cappé,O., Moulines,E. and Rydén,T. (2010) *Inference in Hidden Markov Models*. Springer.
18. Bishop,C.M. (2006) *Pattern Recognition and Machine Learning*. Springer.
19. Baum,L.E., Petrie,T., Soules,G. and Weiss,N. (1970) A Maximization Technique Occurring in the Statistical Analysis of Probabilistic Functions of Markov Chains. *The Annals of Mathematical Statistics*, **41**, 164–171.
20. Moré,J.J. and Thuente,D.J. (1994) Line search algorithms with guaranteed sufficient decrease. *ACM Trans. Math. Softw.*, **20**, 286–307.
21. Baum,L.E. (1972) An inequality and associated maximization technique in statistical estimation for probabilistic functions of markov processes. *Inequalities*, **3**, 1–8.
22. Krogh,A. (1994) Hidden Markov models for labeled sequences. In *Proc. 12th IAPR Int. Pattern Recognition Vol. 2 - Conf. B: Computer Vision & Image Processing. Conf.* volume 2, pp. 140–144.
23. Mao,X. and Hu,G. (2001) Estimation of HMM parameters based on gradients. *Journal of Electronics (China)*, **18**, 277–280.
24. (2013) The On-Line Encyclopedia of Integer Sequences, Sequence A000670, `http://oeis.org/A000670`.
25. Goecks,J., Nekrutenko,A., Taylor,J. and ,G.T. (2010) Galaxy: a comprehensive approach for supporting accessible, reproducible, and transparent computational research in the life sciences. *Genome Biol*, **11**, R86.
26. Tompa,M., Li,N., Bailey,T.L., Church,G.M., Moor,B.D., Eskin,E., Favorov,A.V., Frith,M.C., Fu,Y., Kent,W.J. *et al.* (2005) Assessing computational tools for the discovery of transcription factor binding sites. *Nat Biotechnol*, **23**, 137–144.
27. Burset,M. and Guigó,R. (1996) Evaluation of gene structure prediction programs. *Genomics*, **34**, 353–367.
28. Valen,E., Sandelin,A., Winther,O. and Krogh,A. (2009) Discovery of regulatory elements is improved by a discriminatory approach. *PLoS Comput Biol*, **5**, e1000562.
29. Gerber,A.P., Herschlag,D. and Brown,P.O. (2004) Extensive association of functionally and cytotopically related mRNAs with Puf family RNA-binding proteins in yeast. *PLoS Biol*, **2**, E79.
30. Kershner,A.M. and Kimble,J. (2010) Genome-wide analysis of mRNA targets for Caenorhabditis elegans FBF, a conserved stem cell regulator. *Proc Natl Acad Sci U S A*, **107**, 3936–3941.
31. Gerber,A.P., Luschnig,S., Krasnow,M.A., Brown,P.O. and Herschlag,D. (2006) Genome-wide identification of mRNAs associated with the translational regulator PUMILIO in Drosophila melanogaster. *Proc Natl Acad Sci U S A*, **103**, 4487–4492.
32. Morris,A.R., Mukherjee,N. and Keene,J.D. (2008) Ribonomic analysis of human Pum1 reveals cis-trans conservation across species despite evolution of diverse mRNA target sets. *Mol Cell Biol*, **28**, 4093–4103.
33. Galgano,A., Forrer,M., Jaskiewicz,L., Kanitz,A., Zavolan,M. and Gerber,A.P. (2008) Comparative analysis of mRNA targets for human PUF-family proteins suggests extensive interaction with the miRNA regulatory system. *PLoS One*, **3**, e3164.
34. Hafner,M., Landthaler,M., Burger,L., Khorshid,M., Hausser,J., Berninger,P., Rothballer,A., Ascano,M., Jungkamp,A.C., Munschauer,M. *et al.* (2010) Transcriptome-wide identification of RNA-binding protein and microRNA target sites by PAR-CLIP. *Cell*, **141**, 129–141.
35. Bechara,E.G., SebestyÃľn,E., Bernardis,I., Eyras,E. and ValcÃąrcel,J. (2013) RBM5, 6, and 10 differentially regulate NUMB alternative splicing to control cancer cell proliferation. *Mol Cell*, **52**, 720–733.
36. Wang,Y., Gogol-Döring,A., Hu,H., Fröhler,S., Ma,Y., Jens,M., Maaskola,J., Murakawa,Y., Quedenau,C., Landthaler,M. *et al.* (2013) Integrative analysis revealed the molecular mechanism underlying RBM10-mediated splicing regulation. *EMBO Mol Med*, **5**, 1431–1442.
37. Inoue,A., Yamamoto,N., Kimura,M., Nishio,K., Yamane,H. and Nakajima,K. (2014) RBM10 regulates alternative splicing. *FEBS Lett*, **588**, 942–947.
38. Chen,X., Xu,H., Yuan,P., Fang,F., Huss,M., Vega,V.B., Wong,E., Orlov,Y.L., Zhang,W., Jiang,J. *et al.* (2008) Integration of external signaling pathways with the core transcriptional network in embryonic stem cells. *Cell*, **133**, 1106–1117.
39. Marson,A., Levine,S.S., Cole,M.F., Frampton,G.M., Brambrink,T., Johnstone,S., Guenther,M.G., Johnston,W.K., Wernig,M., Newman,J. *et al.* (2008) Connecting microRNA genes to the core transcriptional regulatory circuitry of embryonic stem cells. *Cell*, **134**, 521–533.
40. Gupta,S., Stamatoyannopoulos,J.A., Bailey,T.L. and Noble,W.S. (2007) Quantifying similarity between motifs. *Genome Biol*, **8**, R24.
